# Elucidating the molecular mechanisms of essential oils' insecticidal action using a novel cheminformatics protocol

**DOI:** 10.1038/s41598-023-29981-3

**Published:** 2023-03-21

**Authors:** Eduardo José Azevedo Corrêa, Frederico Chaves Carvalho, Júlia Assunção de Castro Oliveira, Suzan Kelly Vilela Bertolucci, Marcus Tullius Scotti, Carlos Henrique Silveira, Fabiana Costa Guedes, Júlio Onésio Ferreira Melo, Raquel Cardoso de Melo-Minardi, Leonardo Henrique França de Lima

**Affiliations:** 1 Multicenter Program in Postgraduate in Biochemistry and Molecular Biology, Federal University of São João del-Rei, Campus Divinópolis, Divinópolis, MG Brazil; 2 Minas Gerais Agricultural Research Company (EPAMIG), Pitangui, MG Brazil; 3 Department of Computer Science, Institute of Exact Sciences-ICEx, Federal University of Minas Gerais, Campus Belo Horizonte, Belo Horizonte, MG Brazil; 4 Laboratory of Phytochemistry and Medicinal Plants, Department of Agriculture, Federal University of Lavras, Lavras, MG Brazil; 5 Chemistry Department, Exact and Nature Sciences Center, Federal University of Paraiba, Campus I, João Pessoa, PB Brazil; 6 Technological Sciences Institute, Federal University of Itajubá, Itabira, MG Brazil; 7 Department of Exact and Biological Sciences, Federal University of São João Del-Rei, Sete Lagoas Campus, Sete Lagoas, MG Brazil

**Keywords:** Biochemistry, Virtual drug screening, Cheminformatics

## Abstract

Essential oils (EOs) are a promising source for novel environmentally safe insecticides. However, the structural diversity of their compounds poses challenges to accurately elucidate their biological mechanisms of action. We present a new chemoinformatics methodology aimed at predicting the impact of essential oil (EO) compounds on the molecular targets of commercial insecticides. Our approach merges virtual screening, chemoinformatics, and machine learning to identify custom signatures and reference molecule clusters. By assigning a molecule to a cluster, we can determine its most likely interaction targets. Our findings reveal that the main targets of EOs are juvenile hormone-specific proteins (JHBP and MET) and octopamine receptor agonists (OctpRago). Three of the twenty clusters show strong similarities to the juvenile hormone, steroids, and biogenic amines. For instance, the methodology successfully identified E-Nerolidol, for which literature points indications of disrupting insect metamorphosis and neurochemistry, as a potential insecticide in these pathways. We validated the predictions through experimental bioassays, observing symptoms in blowflies that were consistent with the computational results. This new approach sheds a higher light on the ways of action of EO compounds in nature and biotechnology. It also opens new possibilities for understanding how molecules can interfere with biological systems and has broad implications for areas such as drug design.

## Introduction

Essential oils (EOs) are plant secondary metabolites physiologically important for functions like signaling, pollinator attraction, allelopathy, adaptation to abiotic stresses, and defense against herbivores and pathogens^1^. Their applications in agriculture have been the object of research for several decades, with uses as antifungal, antibacterial, weed control products, and natural eco-friendly insecticides^2^. The use of EOs in pest control has two advantages: first, their low toxicity on mammals, birds, and fish, which translates into safer products; and second, the great structural diversity of their components, making them a potential source of multi-target bioactive molecules^2^.

Current commercially available pesticides and EOs are known to target several specific proteins in insect metabolism. Some of the most common targets for insecticides are of neurochemical/neurohormonal nature: the acetylcholinesterase enzyme (AChE)^3^, neurotransmission regulators such as the γ-aminobutyric acid receptor (GABAaR)^4^, and the Octopamine receptor (OctpR)^4^. Other studied targets for commercial insecticides and EOs are Insect Growth Regulators (IGRs), which affect the timing of molting during insect metamorphosis, particularly the Methoprene-tolerant receptor (MET), being this the physiological receptor of the juvenile hormone (JH); the Ecdysone hormone receptor (EcNR)^5,6^. Besides MET, the JH pathway is also targeted indirectly by enzymes involved in its synthesis and degradation, as well as crucial transporters for this molecule. This last case highlights the juvenile hormone binding protein (JHBP), whose action is reported to be paramount in the adult’s reproductive cycle^7^.

Investigating how the many components of EOs interact with these molecular targets may be the key to understanding why some exhibit insecticide and larvicide properties. Besides the large body of experimental evidence suggesting these EO's properties, more needs to be clarified or better understood about their biochemical mechanisms of action^8,9^. In this task, computational tools are a helpful method for shedding light on how natural compounds act in fly biochemistry. Some computational approaches have successfully demonstrated how some EOs compounds act as inhibitors of mosquito Odorant-Binding proteins^10^. Among these methods, virtual screening, cheminformatics tools, and pharmacophoric methods have been powerful techniques for predicting experimental results^11^. An attractive way to compare a wide diversity of natural molecules is to use metrics of similarities and distance metrics to compare molecular fingerprints^12^. And in this way, the Tanimoto coefficient, which measures the relation of molecule similarities divided by the number of all features between two compounds^13^, is a similarity metric widely used in searching for chemical similarity^12,14^. Molecular descriptors are applied to computational chemistry and effectively establish a fingerprint and properties for molecular structure by calculating Euclidean distance measures between two vectors in cartesian space^15,16^. This metric has been used in establishing chemical molecular similarities^12^. In this work, we use these two metrics to establish molecular relationships among all top hits of docked ligands that interfere with blowfly metabolism. We also predict how essential oils extracted from plants can modify metabolic processes essential for insect survival.

To shed light on molecular mechanisms based on EO's effectiveness as insecticides, we developed an end-to-end protocol that combines chemo/bioinformatics and computational biology approaches. This study aims to establish a computational protocol based on molecular similarity metrics for natural compounds to predict the biochemical interference of EOs in blowfly metabolism. In a case study, this research looks for a blowfly-caused problem named myiasis disease caused especially by flies of the Calliphoridae family^17^. To validate the protocol, it conducts a case study applying *Baccharis dracunculifolia*’s EO on *Chrysomya albiceps* blowfly.

## Results and discussion

### Molecular target selection

To evaluate the insecticidal potential of essential oil compounds, we conducted a brief literature survey to identify key proteins of insect metabolism that can be targeted by pesticides or essential oils compounds, retrieving a total of 1406 scientific papers. Insect neurochemical, neurohormonal, reproduction, and metamorphosis effects were among the most cited terms. To verify how often these terms are associated with common biocide action, we subdivided papers into groups by their biological action in insects (#agitation, #death, #ecdysis … #reproduction, #wing, see all categories in Fig. S1A). Through the plot analysis of Fig. S1A, we had a basis for concluding that it would be more comprehensive if we used targets action-related to neurochemical, neurohormonal, related metamorphosis, or reproduction physiology.

Looking inside these papers, we can see that some ligands and certain insect targets are quite frequent, such as acetylcholine (via acetylcholinesterase), juvenile hormone (via Methoprene-tolerant receptor, 20-hydroxyecdysone (via Ecdysone receptor) and other. Therefore, we decided to make a quantitative analysis (Fig. S1B) searching for the number of papers where these ligands were cited considering the set of insect physiological actions previously established: neurophysiology, metamorphosis, reproduction, and ligands acting on both neurophysiology and metamorphosis (here we first did the intersection of articles from the set of neural symptoms with the set metamorphic symptoms, in other words, papers that had found neural AND metamorphic symptoms sets). Figure S1B plot shows how frequently, in percentage, each receptor-ligand pair was cited among the set of papers related to the insect physiological sets previously defined.

Our final selection criteria were how well characterized the protein's binding site was and if true ligands were reported in the literature.

Based on these criteria, we selected six potential targets for the virtual screening process: the acetylcholinesterase enzyme (AChE), the Methoprene-tolerant receptor (MET), the ecdysone hormone receptor (EcNR), the juvenile hormone binding protein (JHBP), the gamma-aminobutyric acid receptor (GABAaR), and the Octopamine receptor (OctpR) these last two proteins were considered with their possible agonist and antagonist binding conformations^4,18,19^.

The most common target for organophosphate insecticides is the acetylcholinesterase enzyme (AChE)^3,20^, which degrades the neurotransmitter acetylcholine. This enzyme is a cholinergic enzyme present in neural synapses, its inhibition causes acetylcholine accumulation inside the synaptic cleft resulting in fatal nervous system overstimulation. Consequently, this is widely targeted for common insecticides^3,20^.

Insect growth regulators (IGRs) are a class of insecticides known to target nuclear receptors involved in the timing of molting during insect metamorphosis, particularly the Methoprene-tolerant receptor (MET) and the ecdysone hormone receptor (EcNR)^5,6,21^. This pair of ligand-responsive receptors of transcription factor plays a key role in controlling larval development and metamorphosis. By inhibiting them, insecticides disrupt the insect molting and metamorphosis transitions to adult form^6,22^.

Juvenile hormones (JHs) are important insect hormones that retain the larva in the juvenile state. Once the insect reaches its pupae and adult stages, the juvenile hormone binding protein (JHBP) is produced to act as a carriage protein for JHs, protecting it from degradation as it travels through the hemolymph^7^. This protein is essential in ovary maturation and the normal reproductive behavior of adult insect males. Substances such as IGRs can target and interfere with this protein, disrupting the metamorphic signaling and the adult insect’s reproductive performance^7,23,24^. The presence of EcHs or JHs analogous substances on EOs suggests that these compounds have the potential to mimic the natural hormones and naturally control plague infestation^4,9^.

Furthermore, neurotransmission regulators such as the gamma-aminobutyric acid receptor (GABAaR)^4,25^ and the Octopamine receptor (OctpR)^4,21^ are also commonly targeted for insecticides. Ionotropic γ-aminobutyric acid receptors (GABAaR) act in insect neurotransmission in both central and peripheral neural systems. This channel membrane receptor is involved in the neuron hyperpolarization process that promotes muscle relaxation and other functions^4,9^. Insecticides like Abamectin act as agonists, causing lethal nervous system inhibition^9^. Whereas insecticides like dieldrin and fipronil act as antagonists of the GABAaR, causing a fatal overexcitation of the insect nervous system^5^. There are limited data on the EOs' effects on these targets, but it is reported that monoterpenes can increase GABAaR-mediated chloride uptake and allosteric modulation in insects^4^.

The insect octopamine receptor (OctpR) is a G-protein coupled receptor (GPCR) class, which is a canonical target for the prospection of insecticides^9,26^. Octopamine (Octp) is the natural agonist, and its antagonists, like Epinastine (Epin), act as a neurotransmitter, neurohormones, and neuromodulators^4^. In insects’ physiology, this Octopaminergic system modulates muscle activity by directly acting on muscle fibers, changing the basal tonus^27^. The sensory system increases the activity of olfactory neurons, attractive stimuli odor, pheromones and nestmate recognition, auditory frequency tuning, and mating behavior^26^. Recent reports also show that OctpR participates in metamorphosis both through the inhibition of the Juvenile hormone (JH) biosynthesis in insect Corpus Allatum (CA) or by enhancing ecdysone synthesis at insect prothoracic glands (see below)^28^. EOs and other phytochemical compounds are reported to interact with insect OctpR, mainly as agonists (OctpR/ago)^4^.

Investigating how the many components of essential oils interact with these molecular targets may be the key to understanding why some EOs exhibit insecticide and larvicide properties.

### Homology modelling of the targets

Once we selected the molecular targets to use in the virtual screening phase of our protocol, we used homology modeling software (UCSF Chimera, Modeller, Phyre2, and Swiss-Model) to build the corresponding models for the *Chrysomya albiceps* species. After the models were created, we assessed their quality with the following model quality-checking tools: MolProbity^29^, SAVESv6.0 SAVES v6.0 server (https://saves.mbi.ucla.edu/), and PROCHECK^30^. Only the models that passed the quality check were used in the virtual screening to ensure the acceptable quality of the models. The assessment results are presented in Table S2 in supplementary materials, which summarizes the quality parameters generated by each platform with reference values and a brief explanation of how the parameters were calculated. All Models have RMSD lower than 1 considering their templates. the majority of models produced have more than 90% of residues in favored Ramachandran regions and less than 1.5% outliers residues.

We generally observe a good alignment between model and template sequences when analyzing each molecular target's protein binding regions. Figures S2, S4, S6, S8, S10, and S12 in supplementary materials highlight, with a colored box, each of the ligand-binding protein residues for each protein target based on its original template reference publication and its corresponding amino acid residues for the protein model. This is considered proper conservation in amino acid residues involved in the ligand interaction pocket.

This could be better observed in ligand–protein pocket images presented in Figs. S3, S5, S7, S9, S11, and S13, which show the protein model and template pocket. Representing in the sticks and labeled are the ligand-binding protein residues, according to the original template reference publication, for each protein target. In lines, the amino acids residues 8-angstrom farm from crystallographic ligands.

### Cluster archetypical signatures as predictors for the binding potential to different targets

**Figure 1:**
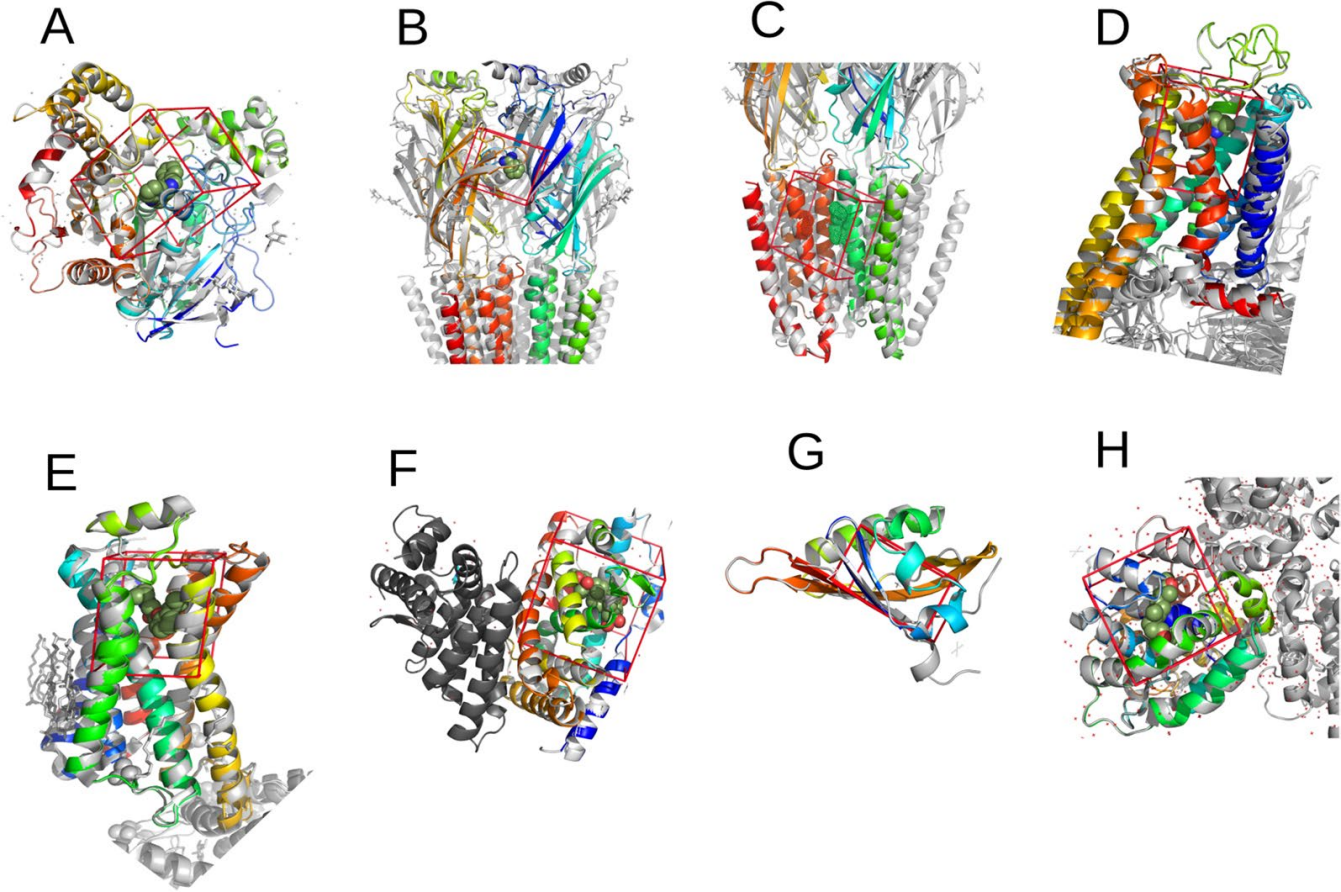
Protein blowfly targets modeled. (**A**) Acetylcholinesterase enzyme, (**B**) γ-aminobutyric acid class a receptor agonist, (**C**) γ-aminobutyric acid class a receptor antagonist, (**D**) octopamine receptor agonist; (**E**) octopamine receptor antagonist, (**F**) ecdysone nuclear receptor, (**G**) methoprene-tolerant juvenile hormone receptor, (**H**) hormone carrier (juvenile odorant family hormone binding protein).

**Figure 2:**
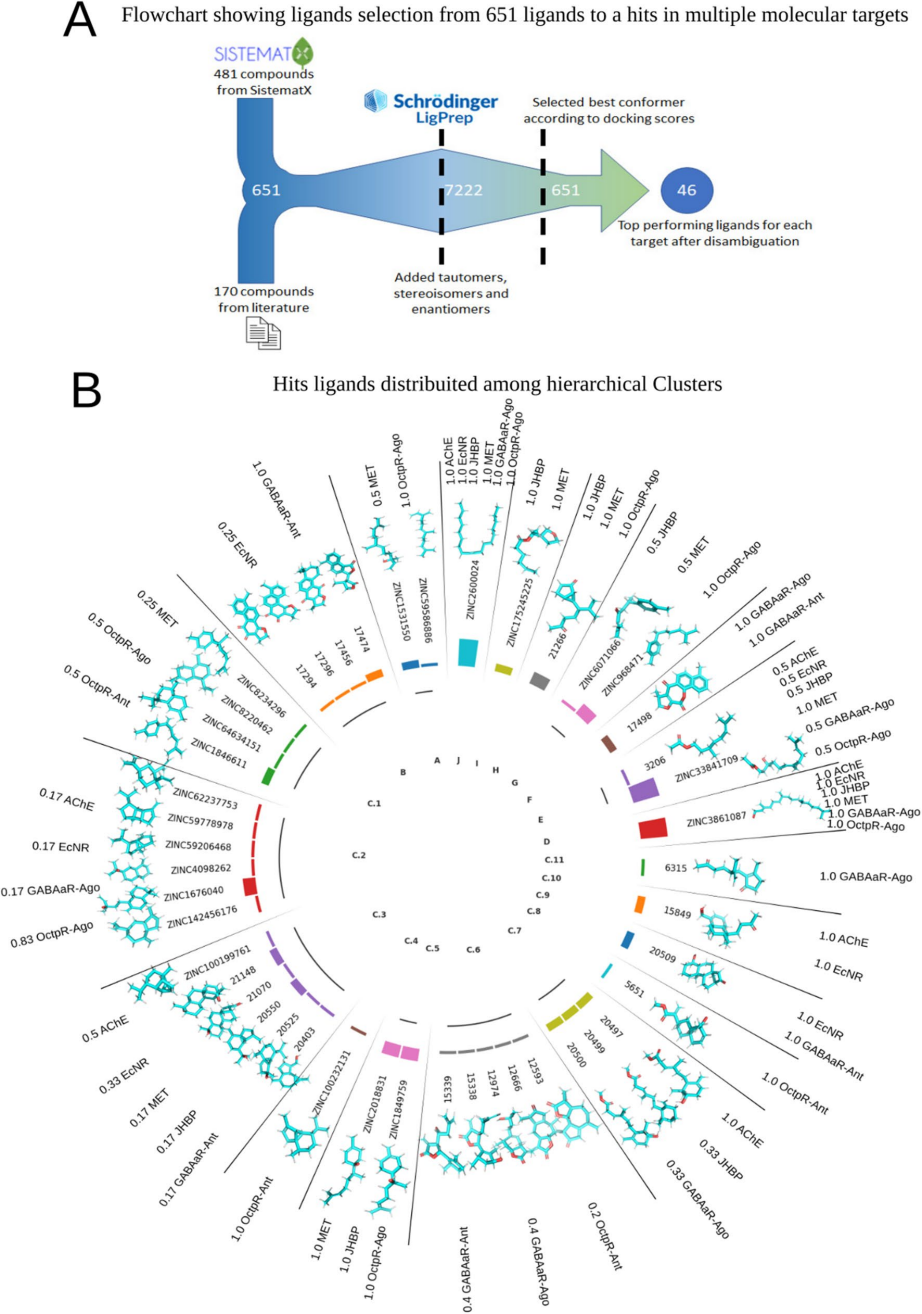
(**A**) Flowchart showing the virtual screening of dataset ligands until achieve the 46 Top hits ligands. (**B**) Distribution of hits ligands for blowfly targets among hierarchical clusters, the bar height represents the number of targets high scored by the ligand. The chemical structure is shown, and numbers represent the probability of a blowfly target being affected by the hierarchical cluster.

After all molecular docking experiments had been performed, the 1% top-scored ligands from all docked ligands for each of the eight blowfly biochemical protein targets were determined, resulting in a subset containing 46 top hits ligands (Fig. 1). Then the Tanimoto coefficient and hierarchical clustering were used to establish clusters of molecular similarity based on the Tanimoto similarity metric (1-Tanimoto Coefficient). The established molecular cluster is presented in Fig. 2 and Fig. S14. In Fig. 2, the bar heights represent the number of protein targets affected by each ligand. Almost all ligands have a unique target protein, but clusters D, E, and J are formed by undiscriminating ligands that bind to six blowfly targets. Clusters are composed, in the majority, of Sesquiterpenes, being of aliphatic (Clusters A, G, H) or alicyclic nature (Clusters C1, C2, C3, C6, H).

The different clusters determined have well-established chemical signatures. These signatures are detailed in Table S7. Cluster A are sesquiterpenes compounds formed by an open chain with three isoprene units. The sesquiterpenes are reported as a repellent and insecticidal agent that can be used in pest control^31^. Their skeleton resembles the chemical topologies of the Juvenile Hormones, the natural ligand of the JH receptor. EO compounds like E-Nerolidol (Cluster A) are reported for insect repellency, larval mortality, and ovicidal activity^32^. A recent study applying E-Nerolidol on Egyptian cotton leaf worms indicates that this sesquiterpene can produce strong larvae toxicity, reducing larval weight gain, prolonging the pupal and larval duration, a dose-dependent pupation inhibition and pupae malformation^33^.

Clusters B, C2, C9, C10, and D are diterpenoid compounds with four rings of carbons in Clusters B and C9. Keto lactone is represented in Cluster F. The Clusters C1, C5, C7, G, and I show a chemical signature of compounds with one ring of carbon coupled with an aliphatic chain (Fig. S14).

**Figure 3:**
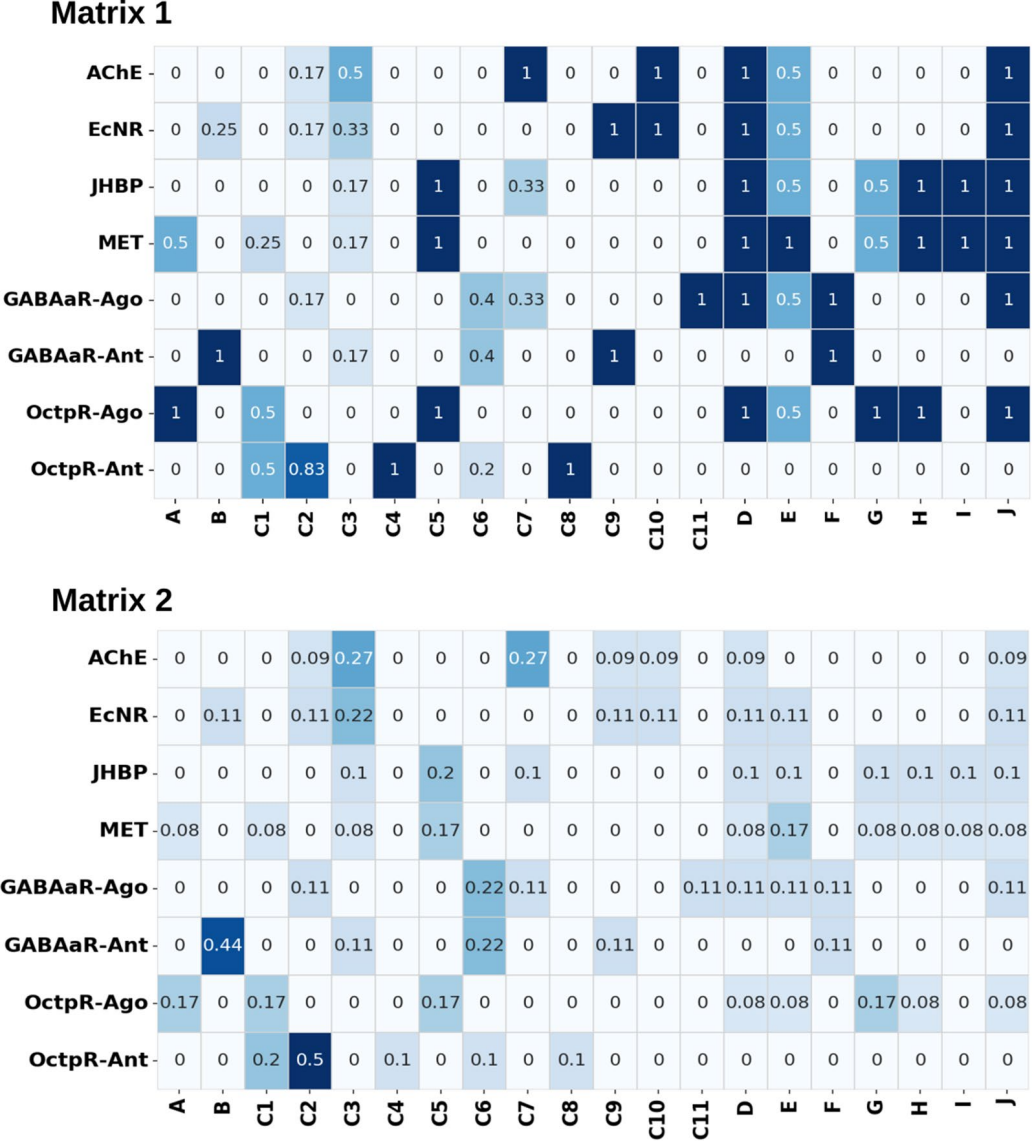
Matrix 1 shows the fraction of hits of ligands belonging to the cluster with high docking scores for the protein target; Matrix 2 otherwise presents the fraction of ligands that affect the protein target which belongs to each cluster.

Diterpenes from cluster B with Phytosteroid architecture (presenting a carbonic skeleton like Phenanthrene with 3 C6 rings connected). In this way, they are structurally similar to the Ecdysone Insect Hormone structure. As shown in Matrix 1 in Fig. 3, these Diterpenoid clusters (C9 and D) have a significant signature to the Ecdysone receptor. Literature relates several abnormalities associated with premature metamorphosis (an ecdysone agonist action) on the larval treated with diterpenoids isolated from EOs capable of assuming Phytosteroid-like topologies^34^.

Sesquiterpene Lactones are represented in Cluster C6. They all have structural variations but with three oxygen in the chemical structure. Sesquiterpene Lactones are relatively well-documented insecticidal and antifeeding activities for insects^35^.

Cluster G presents skeleton topologies like biogenic amines like dopamine and octopamine (without nitrogen atoms and with relatively smaller aliphatic side chains than these amines). Cluster G and biogenic amines show benzene radicals in the *para* position and an aliphatic chain marking position 1 in the benzenic ring. Octopamine and compounds in Cluster G are more topologically related because of the aliphatic lateral chain branching on the first carbon. Dissimilarities between the terpenoids and the biogenic amines are the oxygenation pattern, the length of the lateral chain, and the presence of nitrogen in biogenic amines.

As it could be inferred from the computational analysis, compounds with similarities with cluster G are probability agonist signatures to the Octopamine receptor (see Matrix 1 in Fig. 3). Sesquiterpenes from cluster G with an organic skeleton topologically similar to biogenic amines such as octopamine. Besides, there is no amine group in its skeleton. Therefore, the higher fitting of compounds from this group to the OctpR at the agonist conformation is a consonant issue.

High-chain aliphatic compounds like phytol and 9-Octadecyne of cluster J showed promiscuity for targets (Fig. 2 and S14). This is probably because its chains are easily entangled in multiple configurations and associated with different protein binding sites. Phytol strikes the insect nervous system and causes neurotoxic effects through several mechanisms by inhibiting AChE activity^36–39^, agonist effects like benzodiazepines, producing sedative and anxiolytic activities the GABAa receptor^40,41^, and agonist of octopamine receptors^42^.

It is reported that phytol alters the normal pattern of insect growth and development patterns. A prolonged larval duration when treated with retinol and phytol, a prolonged larval duration indicates larval tendencies to retain their larval stage^43^. However, the biochemical mechanisms by which these effects happen remain unclear.

### Predicting of EOs’ action on the blowfly protein target

The first matrix, Matrix 1, is shown in Fig. 3 and represents the number of compounds belonging to each hierarchical cluster high-scored in virtual screening to bind to each blowfly protein target. In other words, it represents the probability of the hierarchical cluster compounds being highly scored for each target.

The second matrix, matrix 2 in Fig. 3, represents the fraction of the compound high scored in virtual screening for each blowfly protein target that belongs to each hierarchical cluster. In other words, this represents the specificity degree of each protein target for the chemical clusters determined. Considering the values obtained in these results and shown in matrix 2. Antagonists of the GABA receptor (0.44 in cluster B) and octopamine receptor (0.5 for cluster C2) are supposed to be most restrictive to chemical features of compounds present in EOs’.

Matrix BDSTFL is the Molecular Similarity matrix of Degrees of Belonging calculated based on the Tanimoto coefficient determined by Fuzzy Logic determined by the degrees of belonging to the clusters obtained by hierarchical clustering. Read the methods section to see about calculations. And Matrix EPNMCS contains normalized Euclidean proximity of EOs compounds from the 0.1% hits archetypical ligands to each blowfly protein target. This matrix was named the Euclidean Proximity Normalized Matrix to Cheminformatics Space vectors and is calculated as described in the methods section.

**Figure 4:**
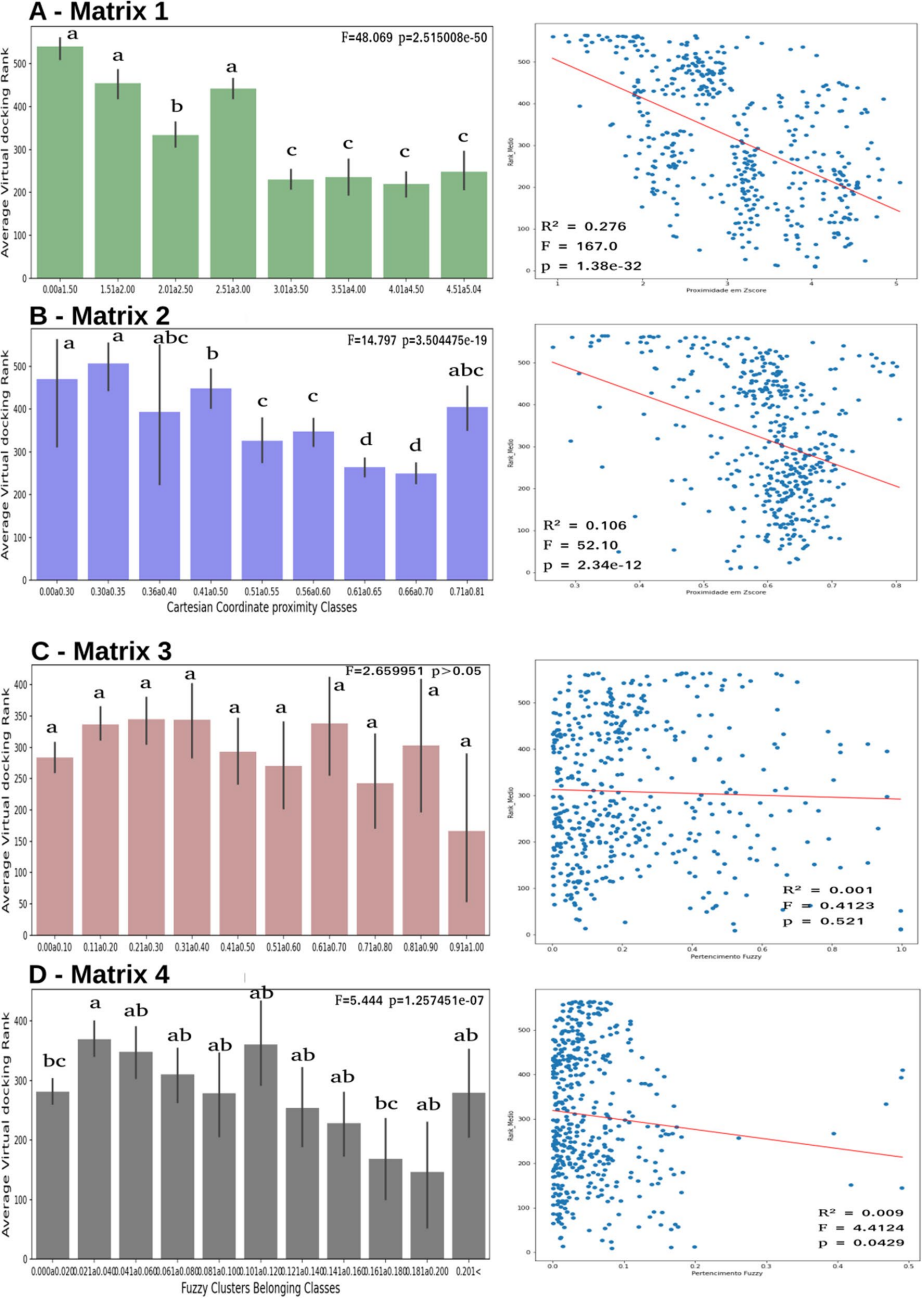
(**A**) Correlation between average docking score rank and Matrix 1: matrix one multiplied matrix EPNMCS; (**B**) Matrix 2: matrix two multiplied matrix EPNMCS; (**C**) Matrix 3: matrix one multiplied matrix BDSTFL; (**D**) Matrix 4: matrix two multiplied matrix BDSTFL.

A pairwise correlation considering the average of docking score rank and, respectively, the multiplication of Matrix 1 and Matrix 2 (Fig. 3) multiplied by matrix EPNMCS and matrix BDSTFL was performed to validate the molecular similarity metrics approach. The correlations are shown in Fig. 4; letters A and B show the correlation considering the similarities calculated considering the Euclidean metric based on molecular descriptors.

Moreover, letters C and D are pairwise correlations considering the topological Matrix based on Tanimoto similarity. The strongest correlation between docking scores and distances obtained from Euclidean metrics can be observed using molecular descriptors, r^2^ = 0.276 and F = 48.069. Therefore, based on these achievements, we consider for further analysis only the Matrix of target versus EOs compounds generated by multiplication matrix of probability to affect each target (matrix 1, in Fig. 3) and Euclidean proximity of each EO compound to hierarchical cluster based on the z-scored molecular descriptor (matrix EPNMCS).

To determine and visualize the behavior of rank points in y ordinate along the measurement parameter for chemical similarity measured by fuzzy logic or QSAR descriptors in abscissa x, we split the abscissa values into categorical classes of 0.1 bins and proceed with an Analysis of Variance (ANOVA) with post hoc mean comparison by Tukey test. Following this procedure, we can construct the Bar plots shown in Fig. 4.

It can be observed in the bar plots shown in Fig. 4 that, statistically, the use of BDSTFL did not return predictable information of rank score in docking of plants EOs. On the other side, if it is considered the EPNMCS metrics, especially when the EPNMCS matrix is multiplied by the matrix of the probability of the hierarchical cluster affecting each target (Fig. 4A—Matrix 1). It is possible to observe the separation into two discrete categories: low Euclidean proximity and low docking scores (indicated by the letters a and b), and proximities values higher than 3.01 z-scores featuring high docking scores (letter c).

**Figure 5:**
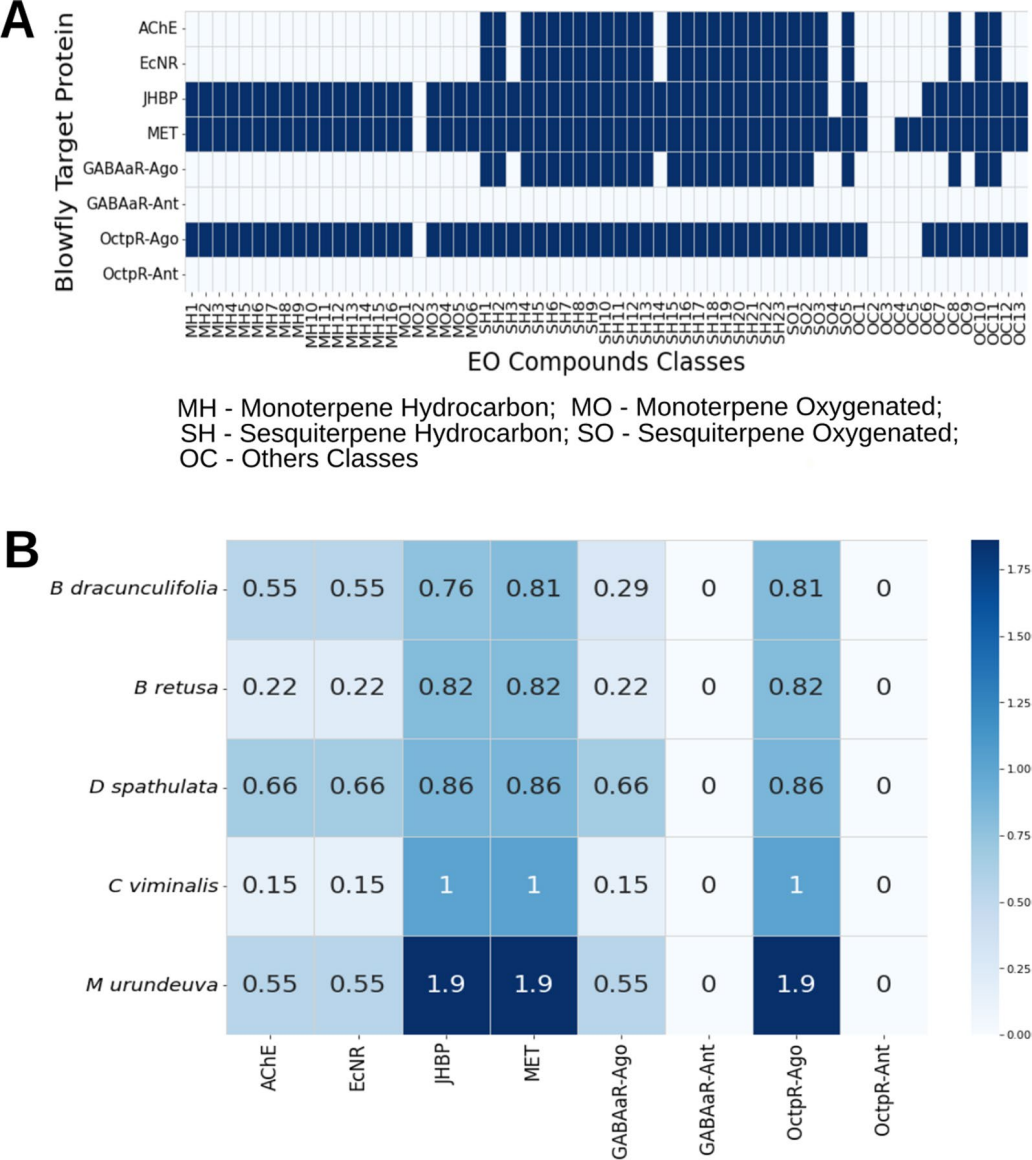
Matrices of the likelihood of plant essential oils compounds affect the blowfly’s protein target low pale-blue and or high deep blue. ID numbers refer to EO compounds in the chemical chromatographic profile of plant species shown in Table S8 and published studies^35,36^.

This statistical information allowed us to build a likelihood Matrix (Fig. 5A), considering values higher than 3.01 have a high probable signature to the protein target, which is a donated value of 1.00. Otherwise, proximity classes with values lower than 3.00 are labeled with zero.

Using this likelihood matrix, we initially selected five plant species to predict the action of plant EOs on the blowfly molecular targets. Three of the species belong to the Asteraceae family (*Baccharis dracunculifolia*, *Baccharis retusa* DC., and *Disynaphia spathutata* (Hook. & Arn.) R.M. King & H. Rob)^44^. Furthermore, the other two species are *Myracrodruon urundeuva* Allem. (Anacardiaceae) and *Callistemon viminalis* (Sol. ex Gaertn.) G. Don (Myrtaceae) was already being worked on by our research group^45,46^.

The prediction of actions was calculated by multiplying the likeliness matrix (Fig. 5A) and the EOs compounds fractions determined from the Profile of Gas Chromatography (Table S8 and published studies^45,46^), resulting in prediction scores in Fig. 5B, which is the degree in z-scores of Euclidean proximity of species EOs concerning each molecular blowfly target. High values in Fig. 5B greater the degree of influence of EOs on blowfly molecular targets.

It is possible to observe that compounds in EOs have high chemical signatures as agonists for the Octopaminergic system, MET Receptor, and JHBP protein. These computational results (Fig. 5B) suggest that plant species' EO act mainly as an agonist of the Octopaminergic system and as a juvenile mimetic hormone with a signature to Methoprene Tolerant bHLH–PAS receptor (MET) and Juvenile Hormone Binding Protein (JHBP). It is possible to see that the mainly predicted influence is the OctpR agonist over the PAS Methoprene Tolerant Receptor (MET). The strongest predicted agonist action for OctpR was obtained for *Myracrodruon urundeuva* EO (1.9 see Fig. 5B). We can observe from the likelihood matrix (Fig. 5A) that monoterpenes and sesquiterpenes have a high probability of affecting these three protein targets. Monoterpenes and sesquiterpenes are always present in EOs. Therefore, it is possible to notice that all plant EOs in nature is expected to exert an action in MET, JHBP, and OctpR in an agonist way. As mentioned above, mono- and sesquiterpenes have repellent and insecticidal properties^4,31^. Sesquiterpenes like E-Nerolidol cause repellency, larval mortality, reducing larval weight gain, prolonging the pupal and larval duration, dose-dependent pupation inhibition and pupae malformation, and ovicidal activity^32,33^. Monoterpenoids like D-limonene, menthol, citronellal, and linalool have been demonstrated to possess insecticidal activity and are currently used commercially as pesticides or repellents^47,48^. It allows us a generalization. It can be said that essential oils have significant insecticidal action because of their content of a variety of mono- and sesquiterpenes that can potentially interact with important molecular targets of the insect, in this case, MET, JHBP, and OctpR.

The specific chirality of EOs compounds can determine stereochemistry dependence for insecticidal activity^49^. However, Cluster's chemical signatures of high-scored compounds to biochemical blowfly targets are robust enough to allow us to calculate metric similarities and compare EOs compound chromatograms from different species.

### Case study: EO applied on myiasis-producing blowfly

Aiming a case study to provide the first experimental validations of the signatures predicted by our methodology, we carried insect dose–response assays for the EO extracted from *B. dracunculifolia* leaves (whose chromatographic profile was submitted to our methodology, resulting in the profile predicted in Figure Bm *top* line). These bioassays, in turn, were carried against the blowfly *Chrysomya albiceps* (Calliphoridae)^50^. *C. albiceps* is a blowfly commonly found in poultry house production and other Calliphoridae flies with health relevance. The application of EO from *B. dracunculifolia* was chosen based on bioassay results already published^51^, high species frequency in all-natural areas^44^, and sufficient EO production per gram of fresh leaves (~ 1 mL per 150 g).

**Figure 6:**
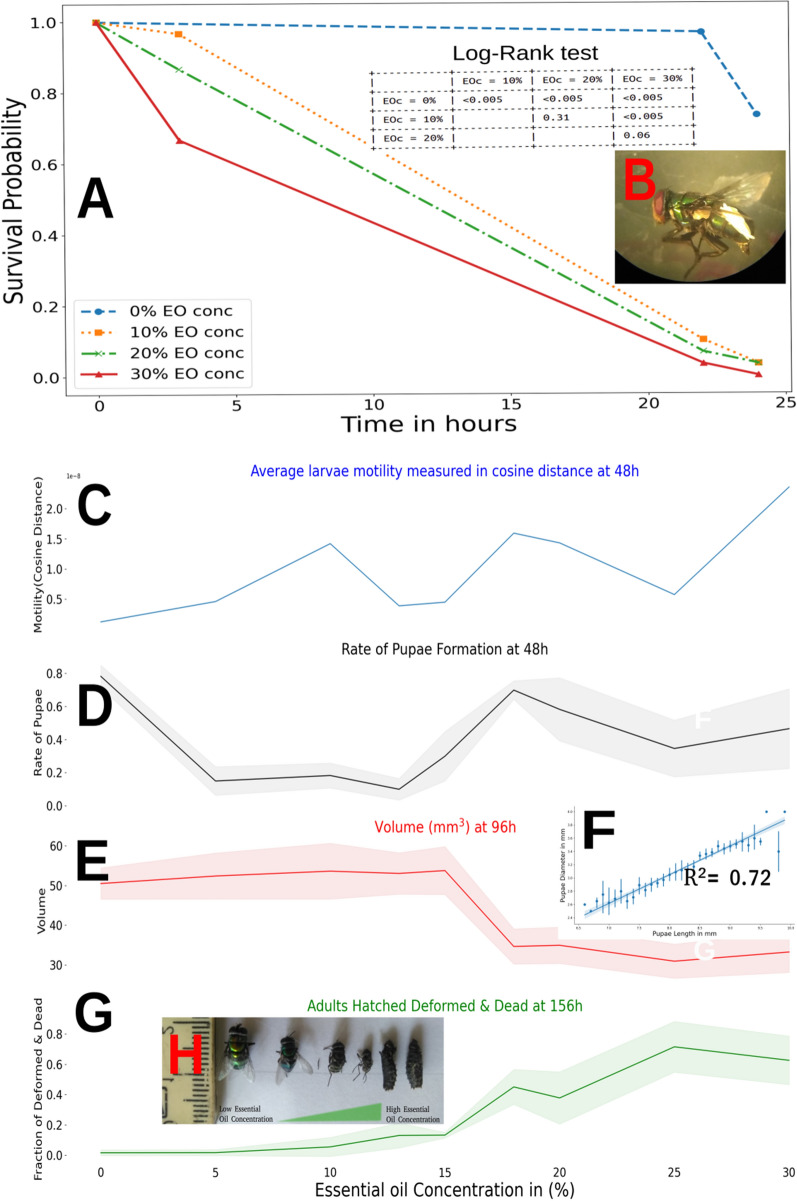
Fumigant activity bioassay showing: (**A**) Kaplan–Meier survival curves and probability log-rank test; (**B**) oviposition by blowflies submitted EO after 22 h of bioassay; (**C**) larval motility measured in Cosine distance over different EO concentration applied; (**D**) pupae rate formation after 48 h after bioassay start; (**E**) pupae volume at 96 h bioassay; (**F**) regression among pupae length and diameter; (**G**) fraction of adults hatched deformed and dead at 156 h of bioassay; (**H**) deformed adults hatched.

In fumigant bioassay on adult blowfly *C. albiceps*, the Kaplan–Meier survival curves, presented in Fig. 6A, and the log-Rank test points out a significatively enhanced kill of adult flies after the application of doses higher than 10%. The 30% of *B. dracunculifolia* EO application significantly has the highest adult blowfly killer action.

A few minutes in contact with EO, it is possible to see a high-frequency vibratory flapping wing (Observational data) and, after 22 h, high oviposition of immature eggs (Fig. 6B). Li et al. (2020) report octopamine-induced oviposition via both α and β octopaminergic receptors in *Plutella xylostella* (Linnaeus, 1758)^52^. Flight muscles are innervated by neurons containing the Octp receptor, and a published report states that octopamine is involved in insects' fast-flight muscle excitation^53^. The Octopaminergic system establishes the octopamine-induced rhythmic motor pattern. It has been demonstrated that applying the Octp antagonist deafferents flight system on locusts (*Locusta migratoria*, Linnaeus, 1758) leads to a wingbeat frequency decrease^26^. These first visual observations of premature oviposition and high frequency of flapping wings with *B. dracunculifolia* assays are, hence, in consonance with the in silico predicted high potential for Octopaminergic agonism in Fig. 5.

**Figure 7:**
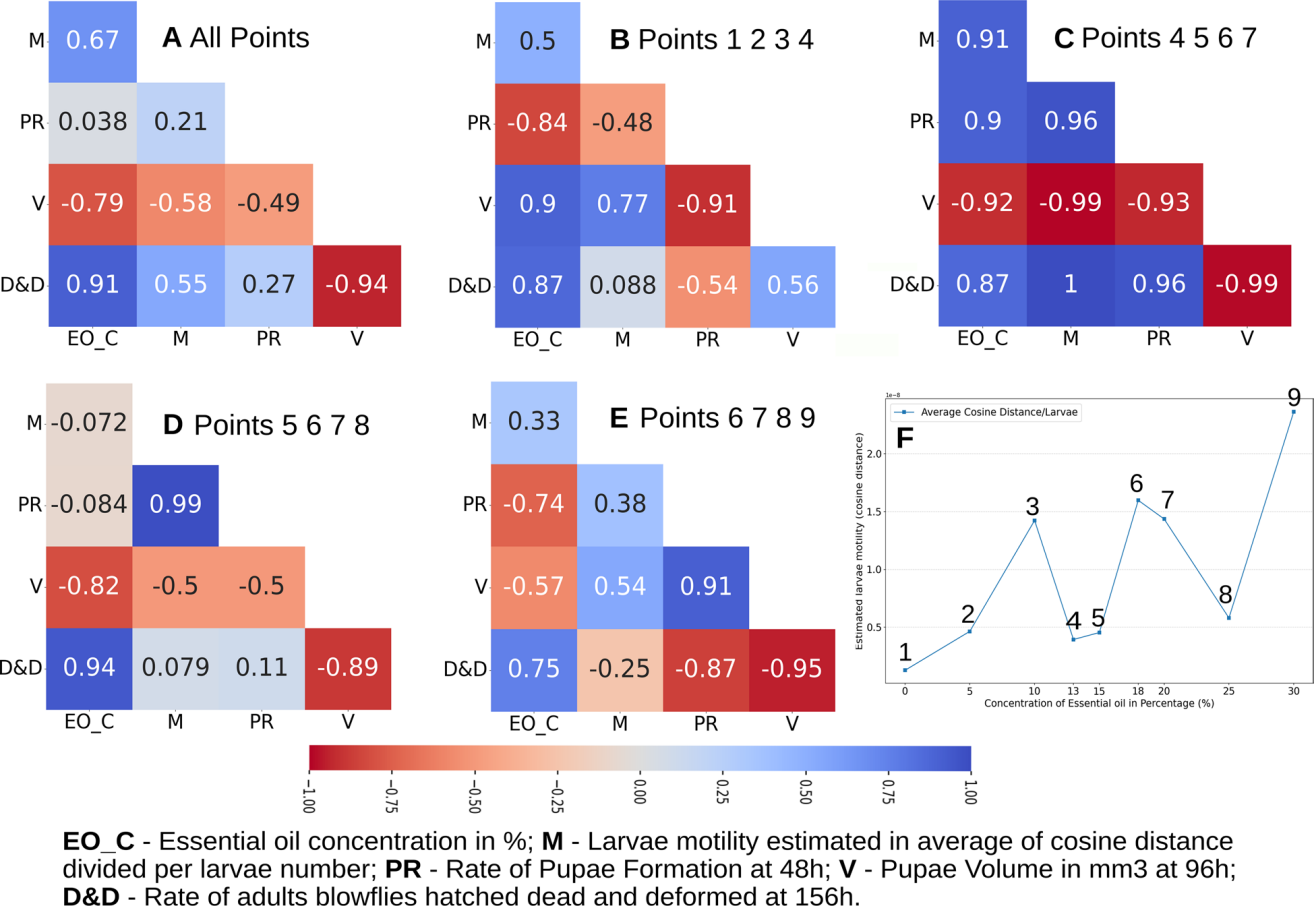
Correlations among larvae motility, rate of pupae formation, pupae volume (mm^3^), and the fraction of adult flies hatched deformed and dead considering the points among (**A**) EO concentrations 0 to 30%; (**B**) EO concentrations 0 to 13%; (**C**) EO concentrations 13 to 20%; (**D**) EO concentrations 15 to 25%; (**E**) EO concentrations 18 to 30%; (**F**) numbering points of larvae motility curve to be correlated with bioassay data.

In Toxicity Bioassay, Larvae motility measured by the frame-by-frame cosine distance video technique, as described in the Methods section, shows a dose-dependent behavior (Videos in V1) presenting a cyclic or periodic response of larvae motility to EO concentration applied. It is possible to see three motility peaks with positive feedback reinforcing loops (Figs. 6C and 7F). Cyclic biochemical responses to octopamine levels in insects play a critical role in the circadian control of olfactory transduction. Pheromone detection thresholds daytime-dependently mediate signals in olfactory learning, memory, circadian rhythms of sleep and activity, juvenile hormone biosynthesis, and the response characteristics of optic flow processing neurons in the fly's visual system^28,54,55^. Therefore, the insect octopaminergic system plays an important role in the circadian or periodic biochemical insect. And in this regard, the ability of EOs compounds to elicit an agonist response by the OctpR receptor could elicit a cyclic response in larvae motility, as observed in Figs. 6C and 7F.

Octopamine (Octp) is the invertebrate norepinephrine-analog, and its physiological effects are related to the "fighting or flight" response. The application of octopamine creates a dose-dependent twitch increase in Australian cricket^56^ and contraction amplitude in various arthropod species^27^. Predicted by in silico analysis (Fig. 5B) and observed in bioassay (Figs. 6C and 7F), the application of EOs on *C. albiceps* larvae shows concentration-dependent like as that observed by Thompson et al. (1990) studying the agonism in the Octopaminergic system of cockroach.

The CA is a high-innervated gland in the insect central nervous system (CNS)^57^. It is largely recognized that CA and JH biosynthesized has a crucial role in insect Metamorphosis, sexual maturation, and reproductive physiology^58^. Octopamine (Octp) modulates many peripheral and sensory organs and, in the nervous system, exerts an inhibition effect on juvenile hormone biosynthesis in a sinusoidal dose-dependent reversible manner^28^. This phenomenon is well documented in cricket and cockroaches, and this biochemical mechanism seems to work on flies^59^.

Larvae motility is controlled by the Octopaminergic system^26,27,56^, and we can see that EO concentration stimuli an agonist of OctpR in a cyclic pattern like that observed by Thompson et al. (1990) in inhibition of JH biosynthesis. When low EO concentration is applied to blowflies, it stimulates the Octopaminergic system. It increases larvae motility at 10% (point 3 Fig. 7F), which is reversed between 13 and 15% (points 4 and 5 Fig. 7F). An enhanced motility loop starts again at 13% EO concentration, which increases to a peak concentration of 18% (points 4 to 6 in Fig. 7F), and the last high motility peak reaches 30% (point 9 in Fig. 7F). Therefore, we can conclude that the cyclic pattern of larvae motility observed in the bioassay is a consequence of an Octopaminergic system response like that observed by Thompsons et al. (1990) when applied to different agonist concentrations, in our case, the EO compounds.

Applying *B. dracunculifolia* EO on the last instar larva causes a sharp decrease in the pupal formation rate (Fig. 6D). The insect metamorphic process is controlled mainly by Ecdysteroid hormones that trigger the insect to molt and Juvenile Hormone, which maintains the youth larvae feature until the right moment for pupae formation.

The EO *B. dracunculifolia* has a strong cheminformatics signature against the MET receptor, decoding a biological signal to delay pupae formation between 5 and 13%. Concentrations above 15% restore the pupal formation rate, like the control group. However, the pupae formed in this treatment have decreased volume (Fig. 6E) and increased the number of adults deformed (Fig. 6G). A decrease in the length and diameter of pupae is only observed at the same time as the second peak in larvae motility (point 5 in motility plot Fig. 7F). When a high concentration is applied, a proportional increase of adults hatched with deformities is observed (Figs. 6G,H).

Based on the computational and Bioassays results, it is possible to infer that *B. dracunculifolia* EO exerts an agonism effect on the octopaminergic system, evidenced by the cyclic in larvae motility over different EO concentrations. Agonism to Octp leads to JH biosynthesis suppression by larvae CA, which breaks the downregulation promoted by the Krüppel-homolog 1 (Kr-h1) transcription factor, which would provoke a change in the timing of the larval metamorphosis, causing malformation and adultoid death at hatching^60^.

Low levels of JH trigger the biochemical process that leads to the pupae phase, as observed by the increase in pupae rate formation, increase in adults hatched dead and deformed, and decrease in pupae volume at concentrations between 13 and 20%.

Adult deformities on blowflies *Cochliomyia macellaria* (Fabrício, 1775) at hatching were also reported by Chaaban et al. (2018) after applying crude EO's *Baccharis dracunculifolia*. Deformities observed by these authors include small size, malformation, poor development, deformed wings and legs, and dried pupae^51^ and reported by Khater et al. (2013) applying crude EOs in *Lucilia cuprina* (Wiedemann, 1830)^61^. Cockroaches' nymphs treated with juvenile hormone mimic compounds died with intact old cuticles, showing an inability to complete the molt. A small percentage hatched, not fully leaving the old cuticle, and eventually died^60^. Molting disruptions were observed after applying excessive amounts of Juvenile hormone or its analogs in *B. germanica,* displaying several abnormal characteristics, including twisted wings and improper sensory organs^60^.

The metamorphosis is the passage of larvae to adult form. From the hormonal point of view, the Juvenile Hormone (JH) produced in insect CA plays an essential role in many aspects of insect physiology. In larvae life, the JH action acts throughout the receptor PAS Methoprene-tolerant (MET) that heterodimerizes with another bHLH-PAS protein Taiman (Tai) and activates the transcriptional factors Krüppel-homolog 1 (Kr-h1), maintaining the larval form^59^. Aliphatic sesquiterpenes present in EO compounds are JH-mimetic which can bind in Methoprene-Tolerant receptor (MET), as denoted in computational prediction Fig. 5B, which can be responsible for the delay in pupal formation rate between 5 and 13% concentration.

The JH in metamorphosis maintains the immature condition, and the progress to pupae-adult insect form will occur in its absence at the end of larval life. Allowing the larvae to molt to pupal form and JH still absent in most pupal life allows the formation of an adult at the next molting^60^. Deficiency in JH promotes precocious metamorphosis and pupal lethality by suppressing the zinc-finger TF Krüppel-homolog 1 (Kr-h1), which acts as the anti-metamorphosis factor^59^. The incorrect JH levels and timing will deregulate the larval-pupal-adult formation. Kr-h1 suppression in the late instar stage induces precocious metamorphosis and adultoid formation, like EOs application^62^.

Pearson correlation of Larvae Motility (M), Rate of Pupae Formation (PR), Pupae Volume (V) and Number of adults Dead and Deformed at hatch (D&D) and EO concentration (EO_C) is presented in Fig. 7. We can observe a positive correlation between D&D and EO_C. In other words, the increase in EO concentration will result in more adults deformed and dead. And a negative correlation between V and EO_C or an increase in EO concentration will result in a decrease in pupae volume (Fig. 7A–E).

Changes in M are biologically attributed to stimulation of the insect octopamine system^26,27,56^, considering the average Pearson correlation of Laval motility with EO_C of 0.67, with V of -0.58, and with D&D of 0.55. We can infer that the OctpR receptor stimulation by increasing the concentration of essential oil is directly related to the reduction of pupal volume and the appearance of deformed and dead adults at the end of Calliphoridae flies’ metamorphosis.

It is interesting to note that at low EO concentrations, it is possible to observe a marked negative correlation (-0.84) between the pupation rate (PR) and EO concentrations of 0 to 13% at the first peak of larval motility (Fig. 7B,F) and that changes drastically change to a positive correlation (0.9) at EO concentrations of 13 to 20 (Fig. 7C,F) corresponding to the second peak of larval motility. But, on average, it shows almost no correlation with EO_C. We can understand that *B. dracunculifolia*’s EO possesses compounds with strong chemical signatures that function as MET receptor agonists, which promote a delay in PR, and compounds with strong signatures as OctpR agonists inhibiting the biosynthesis of the JH. Therefore, the *B. dracunculifolia* EO seems to work as a balanced system, at low oil concentrations, especially by the high composition of a sesquiterpene with a signature for the juvenile hormone, like E-nerolidol, an agonist MET receptor action stands out. And it will result in a PR delay observed between concentrations of 5 to 13% of EO (Fig. 6D). But suppose we start to increase the EO_C application enormously. In this case, it will increase compounds with an agonist signature to the OcptR and induce a dramatic drop in the concentration of JH, increasing the pupation rate (PR) observed in Fig. 6D.

When we slice the motility plot in the increasing phase of the second peak (points 4–5–6–7 in Fig. 7F), which correspond to concentrations between 13 and 20%, the larval motility at these points strongly correlates with pupae rate formation (0.96 Fig. 7C), perfectly fits with a fraction of adults hatched dead and deformed (1.00 Fig. 7C) and is inversely correlated with pupae volume (−0.99 Fig. 7C). It is hard to believe these strong relationships are due to mere chance.

The computational protocol used here helps us to understand the biological phenomena besides the EO's action in insect metamorphosis. The agonist effect of compounds present in EOs can suppress the biosynthesis of JH by CA through the Octopamine receptor, which will increase pupation rate and decrease pupae volume and mainly to abnormalities in adult formation, leading to adults dead at hatch. If it is applied low concentration of *B. dracunculifolia* (5% to 13%) and considering a high concentration of E-nerolidol, an aliphatic sesquiterpene with chemical similarities with JH, in this plant species. We can report an agonist effect on the MET receptor, predicted by the computational protocol, leading to a larvae prolongation life form period, observed in Larval Toxicity Bioassay (Figs. 6 and 7).

## Conclusion and outlooks

In this study, we propose an in silico protocol combining virtual screening, cheminformatics analysis and different metrics of chemical similarity to predict the most probable targets for typical essential oil (EO) components as the chemical activity relationship orchestrating these actions. Firstly, aiming templates for natural insecticide activity, we have used target models from blowflies, an important livestock pest in Brazil and the world. These same proteins are, however, widespread and conserved along a set of other organisms with agricultural and/or medical importance. Besides that, the same methodology can be extended for a virtually unlimited set of other different targets, transcending the relevance of this work to different potential biotechnological applications.

We established chemical archetypes of “strong insecticide targeting” in EO constituents from this mixed computational approach. Some of these archetypical clusters significantly call attention to the chemical signature like canonical natural insecticides (principally clusters A, B and G, respectively composed of juvenile hormone-like, Steroids-like and substances with organic skeletons close to biogenic amine structures). Besides that, natural compounds with known insecticide activity are found between them, such as E-Nerolidol (Cluster A), reported as an insect repellent, larval biocide, and ovicide.

**Figure 8:**
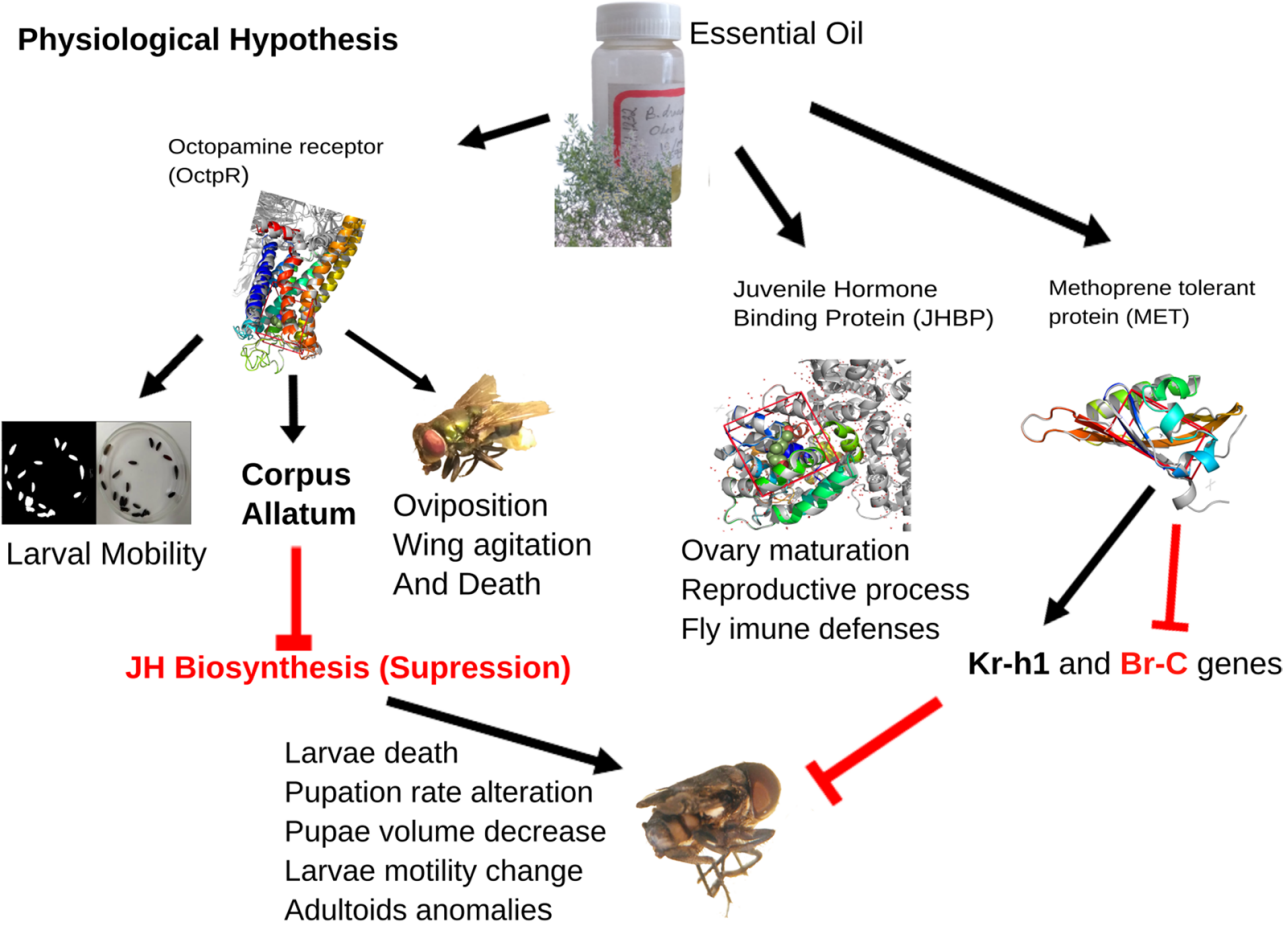
The physiological hypothesis of essential oils' action on blowfly metabolism. Diagram based on the computational and experimental results. Black arrow point induction and red line suppression.

Trying different similarity metrics from these archetypes for compounds profiled from different EOs, we found that a Euclidean distance-based metric on the cheminformatics space has shown a significant relationship with virtual screening scores using properly calibrated score functions for each target. Using this approach, our protocol could point to different compounds with higher insecticide potential for targets studied here on a set of different EOs. Besides that, the global potential for each EO, as a whole, against each target could be equally estimated based on its chromatographic composition, with an interesting prevalence of signatures for juvenile hormone targeting (MET and JHBP) and octopamine receptor agonism in all the EOs here studied. Experimental bioassays with the EO from leaves of *Baccharis dracunculifolia* against *Chrysomia albiceps* adults and larvae demonstrate mobility, oviposition and anti-metamorphic symptoms consonant with these respective signatures, the same being also corroborated by other literature-reported results. E-nerolidol, in the *B. dracunculifolia* case, seems to be the major compound related to this action. A physiological hypothesis linking the major targets pointed by our computational tool to the symptoms observed on the bioassay is present in Fig. 8.

We hope the computational methodology described here and the results presented in our case studies will be valuable in shedding a higher light on the nebulous structure–activity relationship between EO composition and their biological actions. We also hope the same methodology can be used in future prospection studies or even improvement of natural compounds aiming for bioactivity ends. Details of our method calibration and the EO compound database are available in Table S11 at the end of the supplementary material.

## Methods

### Molecular target selection and preparation

To understand how essential oils can impact insect metabolism, we surveyed the literature looking for key proteins of insect metabolism that: (1) are targeted by current commercial insecticides or essential oils, (2) are involved in neurochemical pathways, or insect metamorphosis and (3) have well characterized binding sites. After identifying these proteins of interest, we verified that none had crystallographic structures from the *C. albiceps* species, which was selected as our test subject. Thus, we used homology modeling to create structures for these proteins. First, we screened the *C. albiceps* genome to identify the amino acids or gene sequences corresponding to the selected targets of interest. These sequences were used to search the BLASTp^63^ web server for similar proteins with known structures that could be used as templates for comparative modeling of the *C. albiceps’* target proteins 3D conformations. The templates were selected according to their resolution, sequence identity and coverage. We also prioritized sequences from organisms as closely related as possible to the organism of interest and sequences with known agonist and antagonist structures.

For each molecular target, at least three three-dimensional protein models were created using the following software: Chimera^64^, Modeller 9.25^65^, Swiss-Model^66^, and Phyre2^67^. Due to limitations of the Phyre2 software when generating agonist and antagonist poses, the EcNR, GABAaR and the OctpR were not modeled using this web-service platform. The final models were selected based on quality criteria provided by each modeling tool and web service quality assessment MolProbity^29^, SAVESv6.0 SAVES v6.0 server (https://saves.mbi.ucla.edu/), and PROCHECK^30^.

Proteins target models were generated until achieving a model with (1) a low value of RMSD relative to the template; (2) Catalytic or binding amino acid residues were well positioned as described in references; (3) a Low number of outliers and a high percentage of residues in most favorable Ramachandran plot areas; (4) Best indicators quality generated by the respective modeling software. The model's final quality is presented in Tables S3–S6.

By producing multiple modeling-molecular protein models using different homology-modeling platforms, we can create an experimental design similar to the Randomized Complete Block Design (RCBD), where each model is considered an experimental block, each ligand like a treatment, and each docked pose as a repetition. In further analyses, we will consider the arithmetic mean of the highest docking values and the three highest docking values obtained from docking experiments in each blowfly protein target.

### Ligands dataset preparation

As part of the virtual screening protocol, we performed a series of docking simulations using AutoDock Vina 1.1.2^68^ (Vina) and MGLTools^69^ to evaluate how likely the compounds were commonly found in EOs to bind to the selected blowfly targets. The ligand library contains 481 compounds derived from the SISTEMAT eXtended (SistematX) DataBase^70^ after filtering out compounds in plant EOs. We also added 170 compounds from gas chromatography of five common plant species of interest to the dataset, totaling 651 ligands. These species of interest are *B. dracunculifolia*, *B. retusa*, *M. urundeuva*, *C. viminalis,* and *D. spathulata*.

We eliminated identicalness in the Dataset by calculating the Tanimoto similarity coefficient using the RDKit Python Library^71^. Those with a similarity score of 1.0 were discarded from the Dataset. We created the 3D structure of each remaining compound from their SMILEs using Schrodinger’s Lig Prep^72^, considering a pH of 6.4 ± 0.5 and all possible tautomers and stereoisomers. Thus, our final ligand Dataset has 7222 3D-MOL2 structures.

### Scoring function selection

The scoring functions have an inherent bias that potentially causes a retrieve of false positive ligands during the virtual screening^73^. To reduce this inherent bias, the docking score was estimated with five different functions: Vina^68^, Convex-PL^74^, Ad4_scoring^75^, Dkoes_scoring^75^, and Vinardo^76^. Combining two or more of these individual scoring functions into a consensus score can be more effective in retrieving active molecules and reducing bias by using only score functions known to retrieve positive ligands at the start of the docking process^77^. These five score functions are applied to the docking of the same ligand pose generated by the Autodock Vina. Seeking to reproduce the protein–ligand interaction when establishing a ranking of ligands scores, we first consider the scoring average using only the best pose and, a second time using the average scores from the top three docking poses.

The Vina or Autodock Vina score function is an empirical and knowledge-based scoring function based on the X-Score function^68^. Convex-PL is a knowledge-based score function^74^. The last three scoring functions are Smina, a fork of Autodock Vina that can be customized to improve performance using high-performance energy minimization^75^. Ad4_scoring is a semiempirical Autodock 4 AMBER force field function that uses the Coulomb potential, Lennard–Jones potential, desolvation, and potential conformational entropy related to the number of rotational bonds^69^. The Dkoes_scoring function is the custom function whose parameter function terms and the methodology to develop it is described by Koes et al. (2013)^75^. Vinardo scoring function has a modified combination of weights parameters to determine atomic radii and a lower number of parameters to describe simplified atomics interactions compared to Vina^68^.

For each molecular target, it is determined the score functions which efficiently recover true ligands. We analyzed the behavior of their receiver operating characteristics curves (ROC curve) and the area under the ROC curve (AUC), as well as the enrichment factors curve (EFs curve) calculated by the formula below. It is maximum (EFmax)^78,79^. Firstly, we identified the true ligands by surveying the literature for bioassays using the target protein expressed in a cell or in vitro*,* considering the agonist or antagonist conformations^78^. Ligands with reported IC_50_, EC_50_, K_i_, or K_d_ metrics were selected as true ligands (Table S10) and then generated decoys using the DUD-E web service^80^. The validation set of ligands was submitted to docking simulations using Vina and rescored according to all the additional scoring functions, resulting in five different rankings. Finally, we used these rankings to build the ROC and EF curves for every scoring function in Figs. S13– S20^79^.

$$EF=\frac{N \, true \, positive \, at \, docking \, timeline \, \frac{point}{N } \, total \, ligands \, docked \, at \, docking \, timeline \, point}{N \, total \, true \, positive \frac{database}{N } \, total \, of \, ligands \, database}$$

### Docking score function selection

A good scoring function is expected to rank all the best ligands before the decoys when performing docking simulations. In this ideal situation, the ROC curve would rapidly reach 100% of True positives, resulting in an AUC value of approximately 1, and the EFs curve would peak as soon as the point corresponding to the percentage of the database that corresponds to the good ligands, as shown in the equation below. We established that proper scoring functions would have an AUC value of 0.5 or greater and reach EFMax no later than 5% of the base (1.609 in the log scale shown in Figs. S15–S22). We evaluated these curves in two ways: (1) using the best average score of each ligand across all models for a given molecular target and (2) using the average of the top three scores of each ligand across all models of a target. We then selected the approach that gave the best combination of AUC and EFMax.

At least one score function was chosen for each protein target (highlighted in yellow in Figs. S15–S22). For AChE, the mean of the top three scores is selected using the functions Vina, Convex-PL, and Vinardo (AUC = 0.753, 0.798, and 0.708; and EFmax = 24.4057, 24.4057 and 4.0677, respectively). EcNR was evaluated using all score functions (AUC: Vina = 0.475, Convex-PL = 0.636, Ad4_scoring = 0.604, Dkoes_scoring = 0.604, Vinardo = 0.587; EFmax: 25.15, 40.24, 15.1, 15.1 and 37.72, respectively). For JHBP we selected Convex-PL (AUC = 507, EFmax = 3.72) and Vinardo (AUC = 0.888, EFmax = 6.92). For MET, we considered the mean of the top three scores using Convex-PL (AUC = 0.728, EFmax = 6.22) and Ad4_scoring (AUC = 0.862, EFmax = 16.60). GABAaR in agonist and antagonist conformation has used the mean of the top three scores. In agonists we selected Ad4_scoring (AUC = 0.335, EFmax of 3.71) and Dkoes_scoring (AUC = 0.324, EFmax = 1.79). As for the antagonist conformation, the only score function chosen was Vina (AUC = 0.88 and EFmax = 5.61). The OctpR agonist has used the mean of the three highest score values, and the Vina (AUC = 0.565, EFmax = 12.10), Ad4_scoring (AUC = 0.519, EFmax 18.15), Dkoes_scoring (AUC = 0.497, EFmax = 5.18), Vinardo (AUC = 0.629, EFmax = 12.10) functions. In antagonist OctpR, we used only the best average score and the functions Dkoes_scoring (AUC = 0.776, EFmax = 12.40) and Vinardo (AUC = 0.353, EFmax = 34.08).

### Docking results post-processing

We adopted a clustering-based consensus approach to consider the scores given by the different functions and determine the high-scored overall natural dataset compounds. For each target, the N-selected scoring functions according to the ROC and EF approaches were used to generate N-dimensional vectors containing the scores given by each function to that ligand as elements. These vectors were used to perform successive k-means clustering, and, in each iteration, the cluster with the best combination of scores was kept. The procedure was repeated until only 8 to 12 unique ligands (not considering enantiomers) were selected for each target.

A final set containing the top ligands of every target was constructed, and after eliminating duplicates, the pairwise Tanimoto distance was calculated. This distance metric was used for hierarchical clustering to establish clusters of chemically similar compounds, the cutoff is determined using the procedure displayed in Fig. S23. Each cluster was related to the targets by counting the percentage of the target’s top ligands that belonged to it.

A k-means clustering procedure using the centroids and Euclidean distance was applied to allocate the EOs compounds from the five plant species of interest to each cluster. It enables us to compare a wide diversity of natural molecule similarities using different metrics to compare molecular fingerprints^12^. The Tanimoto coefficient is a widely used metric for chemical similarity^12^. In the end, it is possible to validate the degree of influence of each EOs extracted from different plant species in the blowfly protein target.

In other words, the 651 unique natural compounds in the dataset. K-means clustering was applied successively on the results data considering only the best score functions of Enrichment and ROC curves analysis until the 8 to 12 top hits ligands for each blowfly target.

Cheminformatics molecular descriptors were determined using free tools Molinspiration web service^81^, ACD/ChemSketch^82^, and web service 3D-QSAR.com^83^. The descriptors' values are z-scores to allow the possible comparison among them, doing

$$z \, Scored \, Molecular \, Descriptor=\left(\frac{value-mean \, of \, all \, values \, for \, each \, descriptor}{standard \, deviation \, of \, all \, values \, for \, each \, descriptor}\right)$$

After that, the Cartesian Euclidean distances are calculated for each compound to each cluster defined as mentioned above. A relative normalization and a conversion to the measure of proximity were necessary; therefore, to normalize the values, we divide all Cartesian Euclidean distances by the higher distance obtained, making all Cartesian distances between 1.0 and 0.0:

$$Normalized \, Euclidean \, Distance=\left(\frac{value \, of \, Euclidean \, distance}{higher \, Euclidean \, distance}\right)$$

The measured proximity was calculated by subtracting the Normalized Euclidean Distance from 1. At the final calculations, we obtain a matrix of normalized proximity from the 0.1% hits ligands to each target.

### Experimental blowfly symptoms caused by essential oil application

The experimental assays were done using the *C. albiceps*^50^, a blowfly frequently observed in the Minas Gerais state, Brazil, poultry houses. The blowflies were captured and kept in plastic cages with sawdust on the ground and fed with fresh beef. The EOs used in the experiment were obtained by Hydro-Distillation for 3 h in Clevenger apparatus using 150 g of fresh. The yielded EOs are conserved at −5 °C and sheltered from light^84^. Quantitative and qualitative chemical analyses of EOs extracted were performed according to the methodology adopted by Oliveira et al. (2022)^85^, with modifications. Chemical constituents were identified by comparing their retention rates against a standard solution co-injection, library databases, and literature^86–88^. The retention rates were calculated by the equation of Van Den Dool and Kratz (1 963), and the retention rates described in the literature were consulted^86–89^.

#### Fumigant activity bioassay

Five days-old blowflies’ adults were used on a Fumigant Toxicity bioassay. Ten adults were placed inside Erlenmeyer glasses and closed; a 10 cm cotton string, previously soaked with a 1 ml treatment solution ranging from 0 to 30% of *B. dracunculifolia* Essential oil, immersed in the solution for one minute, was fixed in the middle of Erlenmeyer’s lid^90^. Mortality (%) and qualitatively abnormal behavior were registered at one hour, three hours, 22 h, and 24 h. Each treatment was repeated three times.

#### Larval toxicity bioassay

Larvae showing site-seeking behavior for pupation were used immediately in larval toxicity bioassays. Twenty larvae were treated with an EO serial dilution ranging from 0 to 30%, following the protocol established by Chaaban et al. (2018)^44^. Survivors' number, morphological and behavior change, pupae dimensions, and adult morphological aspects were evaluated every 12 h until the adult emergence. Ten-second videos of the larvae were recorded using Samsung Galaxy A30s at forty-eight hours after the treatment. These videos were stabilized and standardized to 60 frames per second using DaVinci Resolve^91^ software. A custom python script using the OpenCV 4.5.4 library^92^ was created to measure the average larval motility by (1) creating a black and white image, where white pixels corresponded to the larvae and had a value of 1 while background pixels were black with a value of 0; (2) flattening the matrix corresponding to the frame to obtain a vector; (3) measuring the cosine distance between each frame and its predecessor; (4) averaging the measured distances. The cosine distance of two vectors equals one minus the cosine of the angle between them. It is a more suitable proxy for the larval motility than the Euclidean distance because it is indifferent to the length of the vector (which is proportional to the number of white pixels in the image), which translates into a more reliable comparison between videos in which larvae sizes (thus the number of white pixels) might vary.

## Supplementary Information

Supplementary Information 1.

Supplementary Information 2.

Supplementary Information 3.

Supplementary Information 4.

## Data Availability

The data to reproduce the experiments performed here and the scripts used are available in the following GitHub repository: https://github.com/fccarvalho2/Essential_oil_Screening.git.
