# Supplementary figures and images for: Elucidating the molecular mechanisms of essential oils' insecticidal action using a novel cheminformatics protocol
Source: Sci Rep. 2023 Mar 21;13:4598. doi: 10.1038/s41598-023-29981-3 (PMC10028760; doi:10.1038/s41598-023-29981-3)

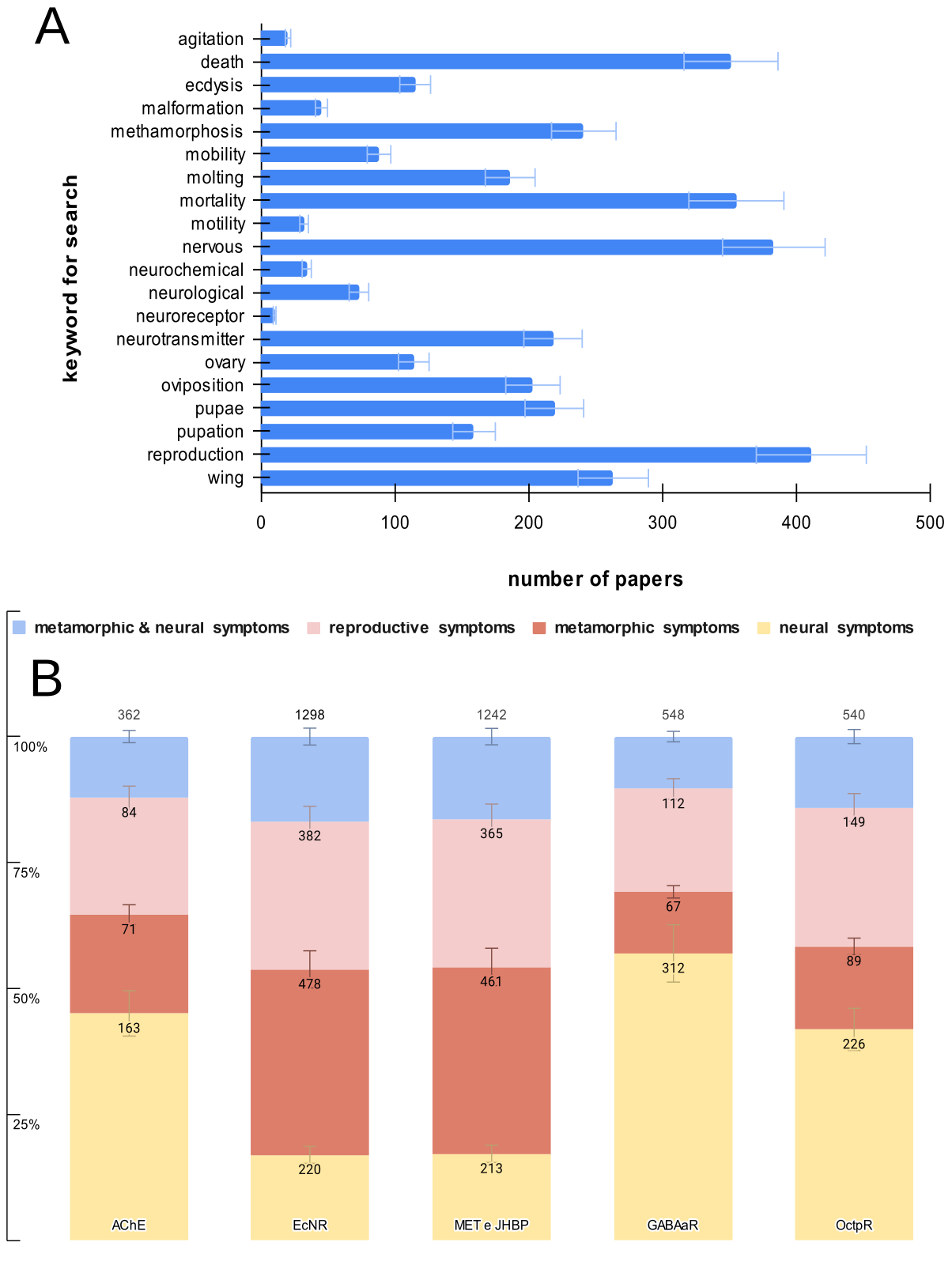

Supplement: Supplementary file 1 — Supplementary Information 1. [file 41598_2023_29981_MOESM1_ESM.zip › FigureS1.png]

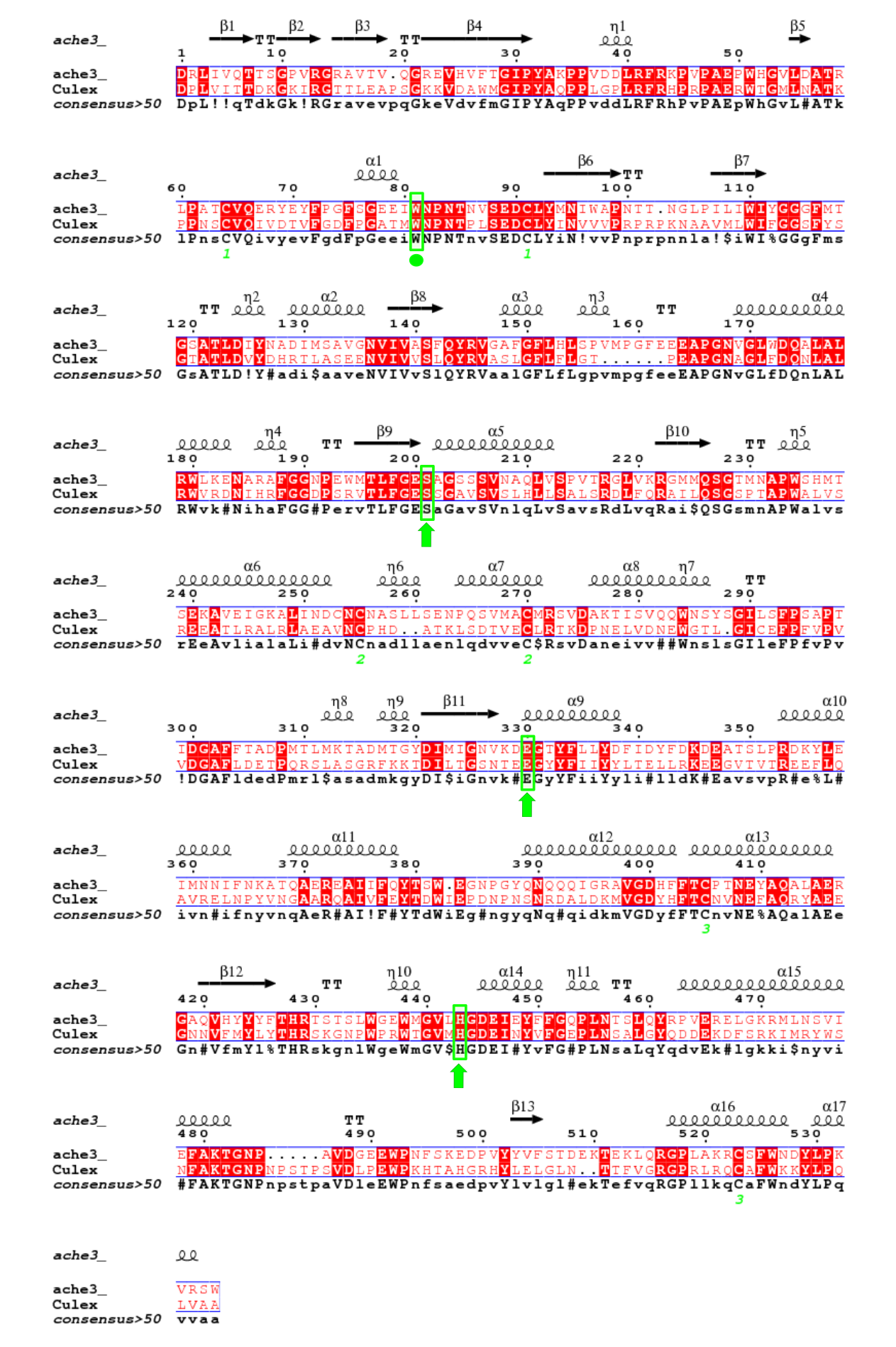

Supplement: Supplementary file 1 — Supplementary Information 1. [file 41598_2023_29981_MOESM1_ESM.zip › FigureS2.png]

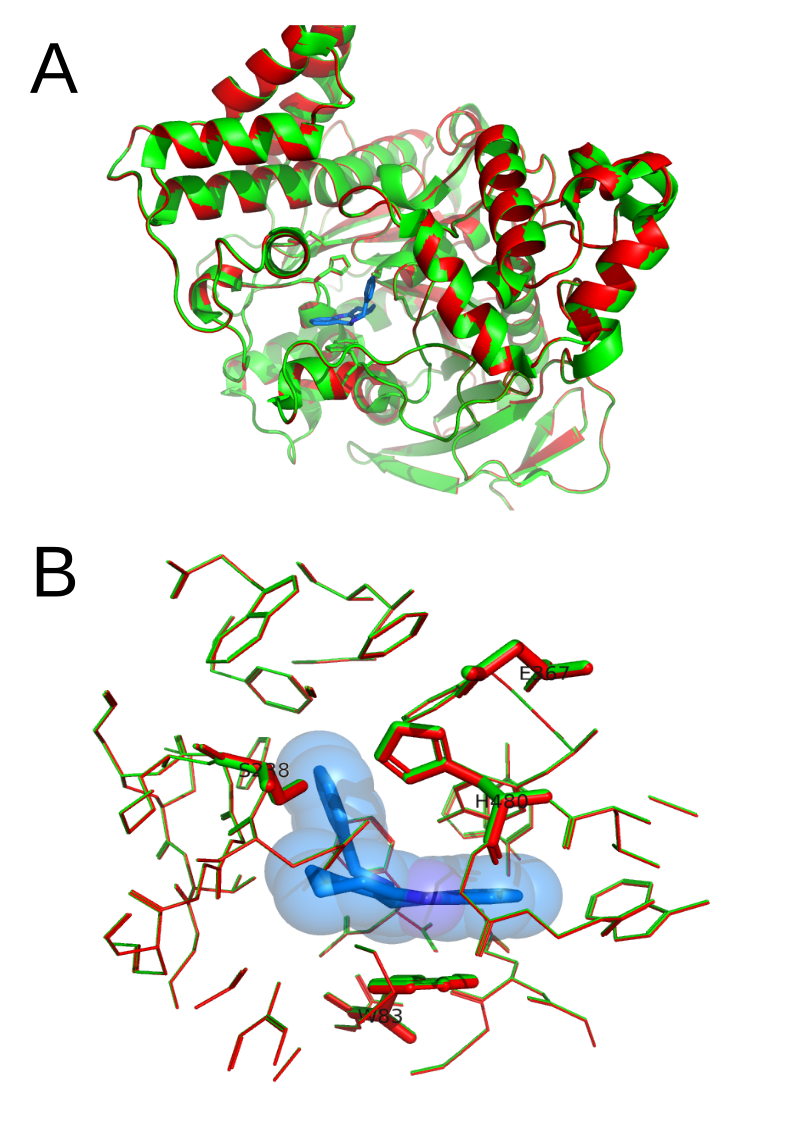

Supplement: Supplementary file 1 — Supplementary Information 1. [file 41598_2023_29981_MOESM1_ESM.zip › FigureS3.png]

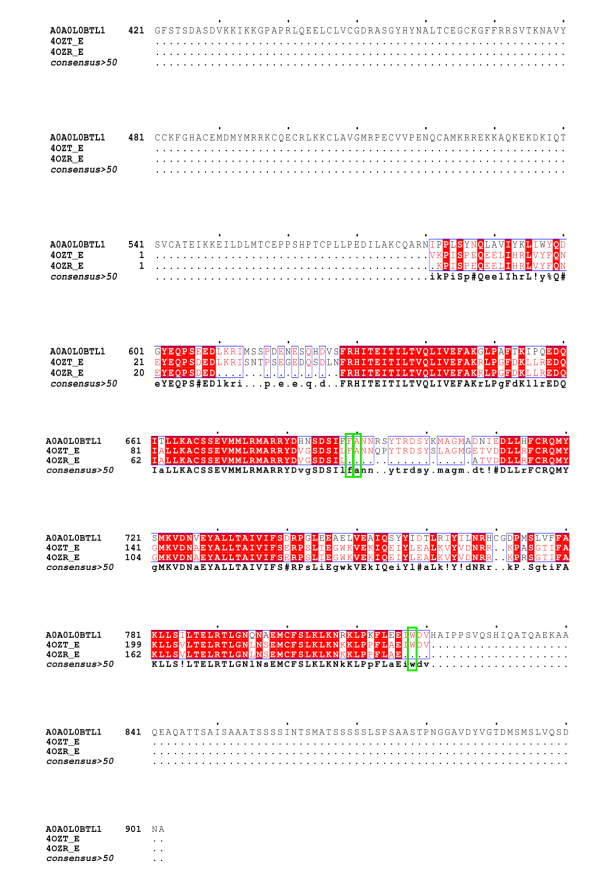

Supplement: Supplementary file 1 — Supplementary Information 1. [file 41598_2023_29981_MOESM1_ESM.zip › FigureS4.png]

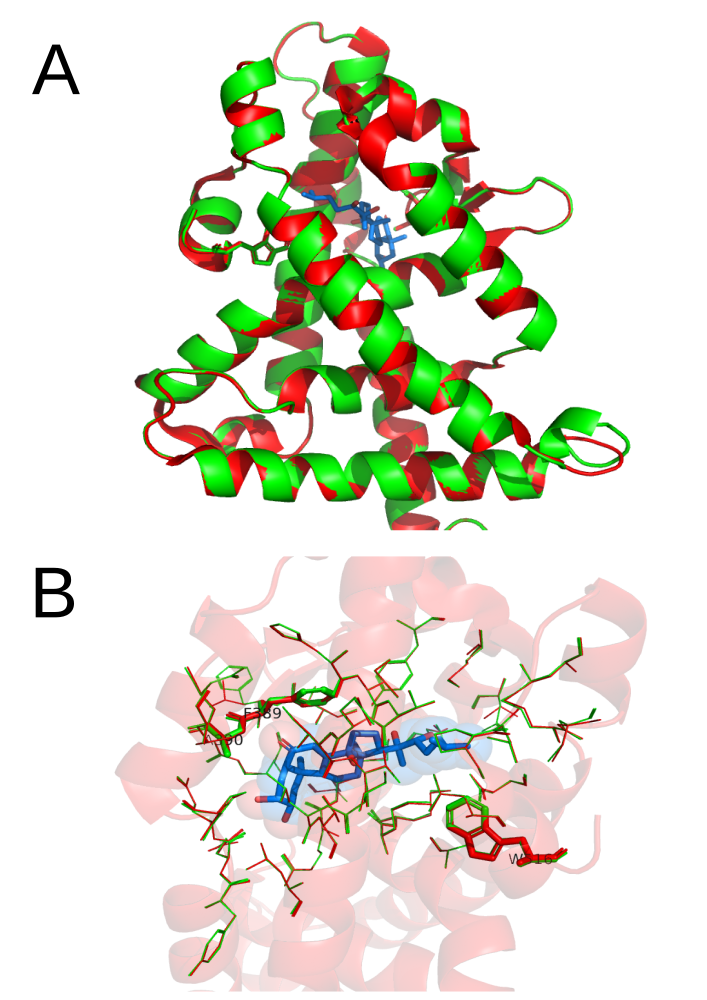

Supplement: Supplementary file 1 — Supplementary Information 1. [file 41598_2023_29981_MOESM1_ESM.zip › FigureS5.png]

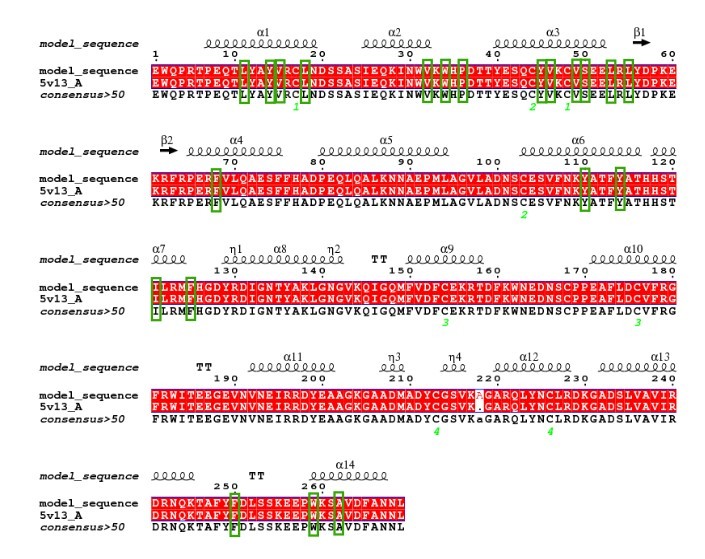

Supplement: Supplementary file 1 — Supplementary Information 1. [file 41598_2023_29981_MOESM1_ESM.zip › FigureS6.png]

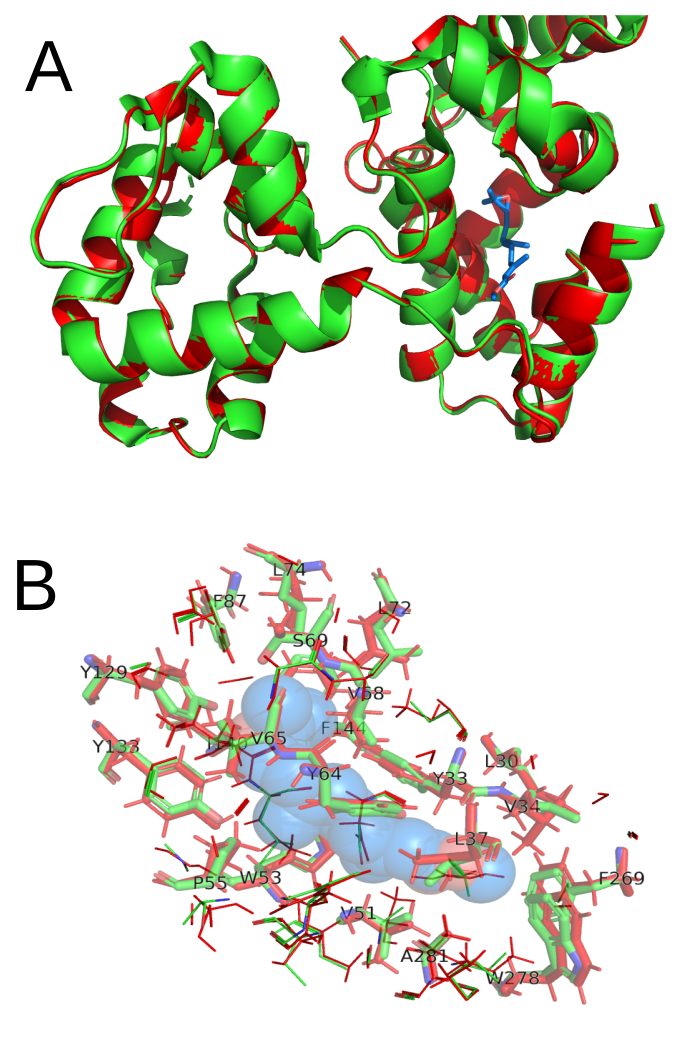

Supplement: Supplementary file 1 — Supplementary Information 1. [file 41598_2023_29981_MOESM1_ESM.zip › FigureS7.png]

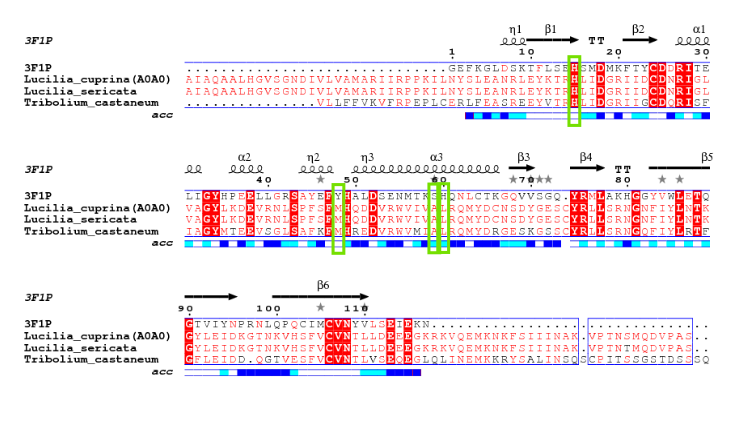

Supplement: Supplementary file 1 — Supplementary Information 1. [file 41598_2023_29981_MOESM1_ESM.zip › FigureS8.png]

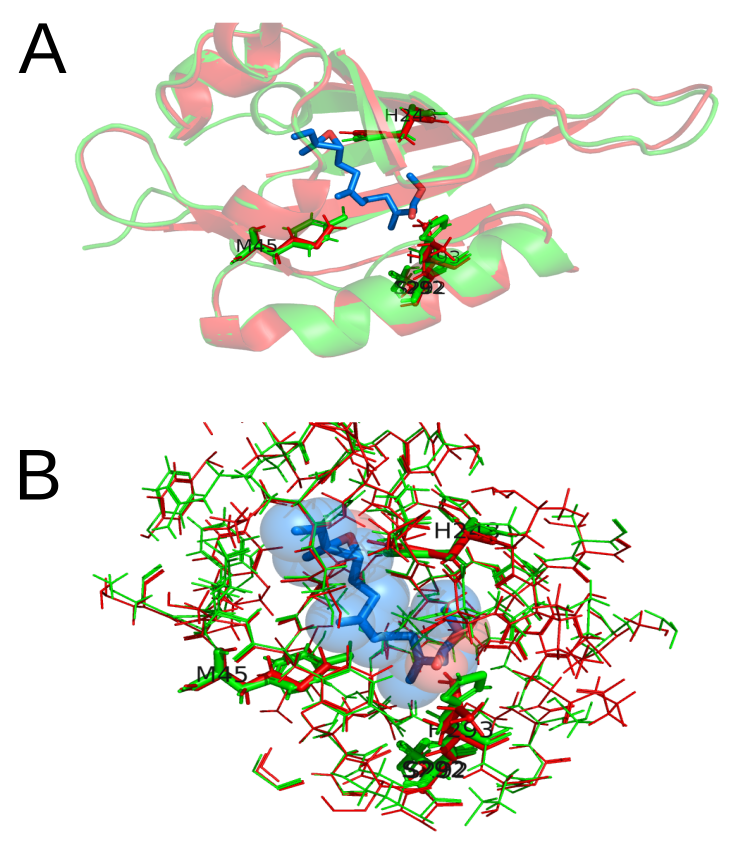

Supplement: Supplementary file 1 — Supplementary Information 1. [file 41598_2023_29981_MOESM1_ESM.zip › FigureS9.png]

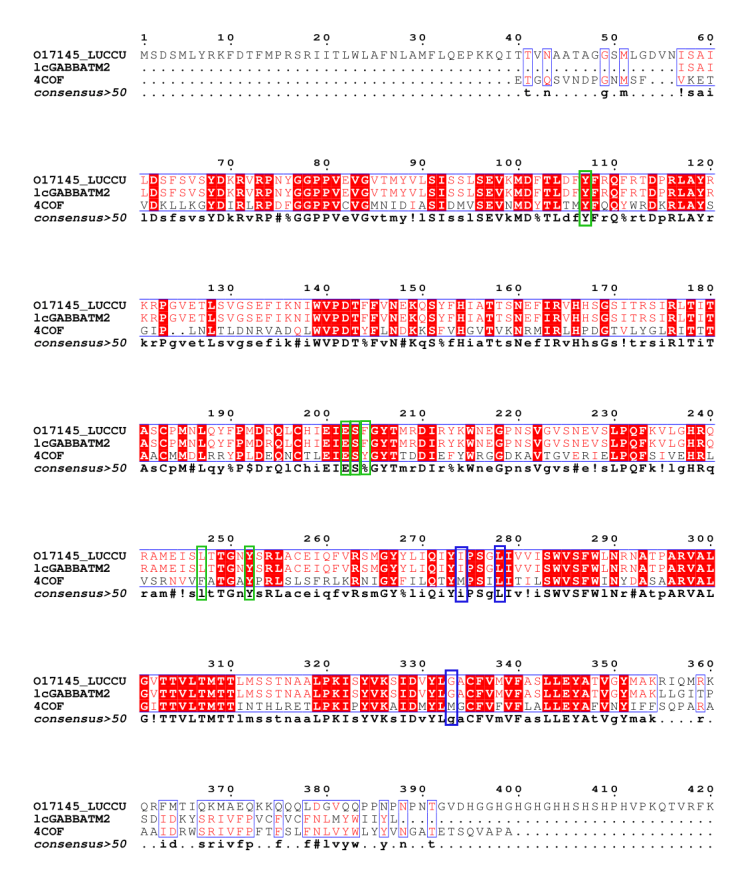

Supplement: Supplementary file 1 — Supplementary Information 1. [file 41598_2023_29981_MOESM1_ESM.zip › FigureS10.png]

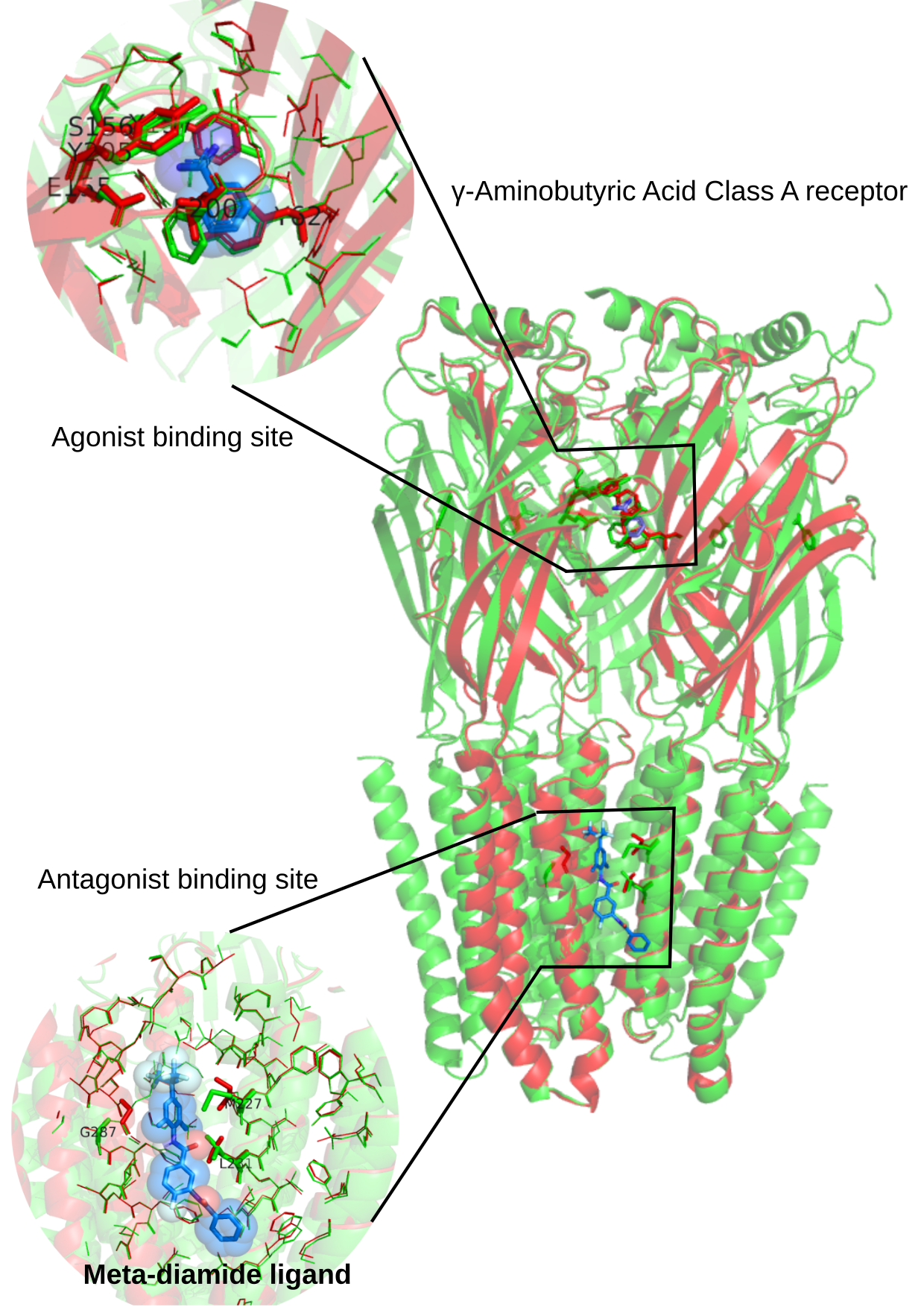

Supplement: Supplementary file 1 — Supplementary Information 1. [file 41598_2023_29981_MOESM1_ESM.zip › FigureS11.png]

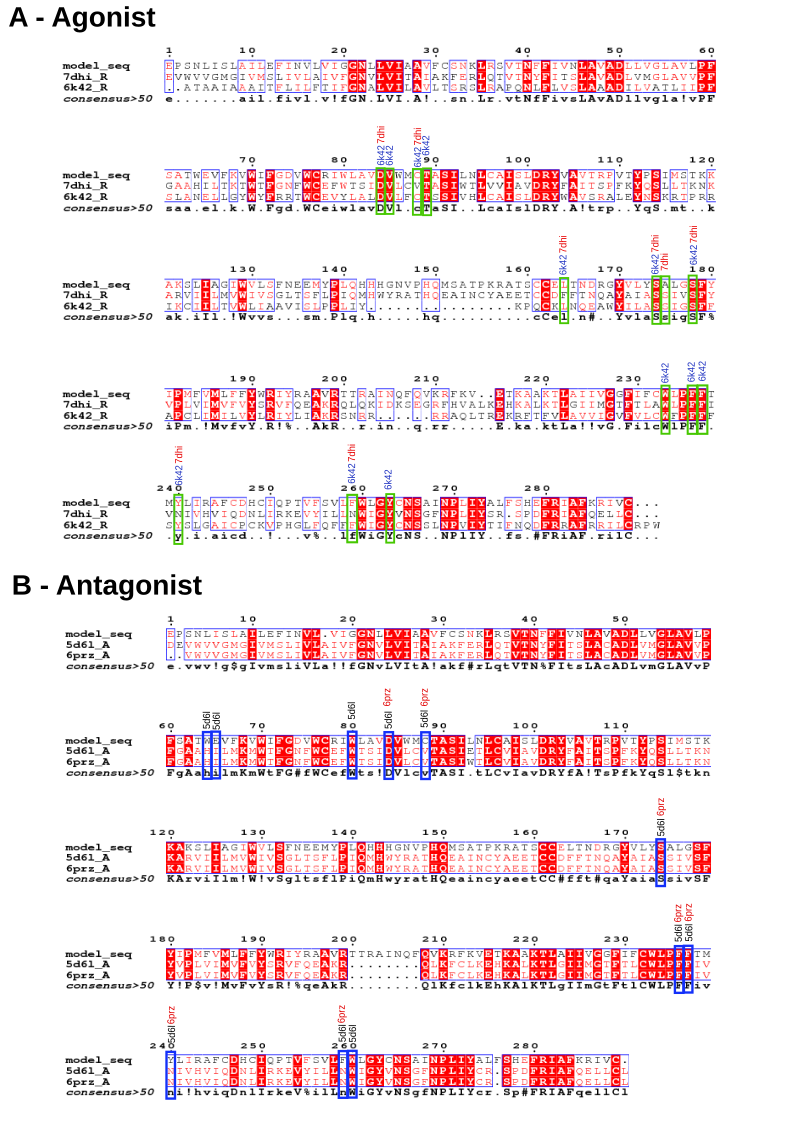

Supplement: Supplementary file 1 — Supplementary Information 1. [file 41598_2023_29981_MOESM1_ESM.zip › FigureS12.png]

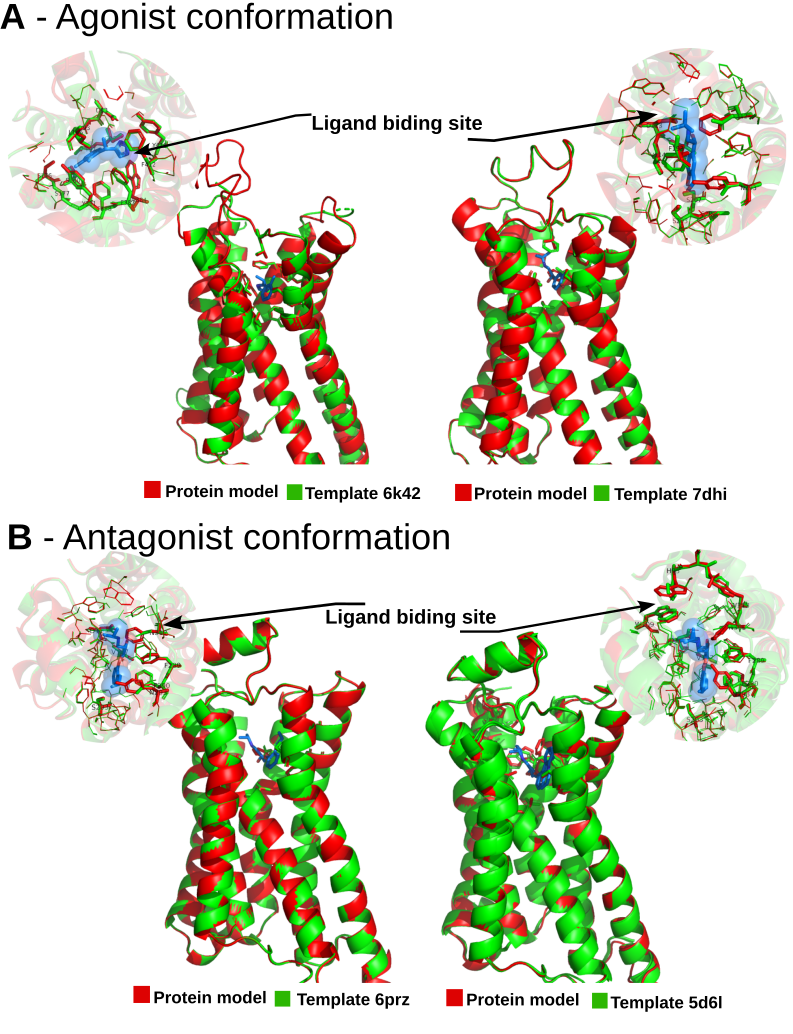

Supplement: Supplementary file 1 — Supplementary Information 1. [file 41598_2023_29981_MOESM1_ESM.zip › FigureS13.png]

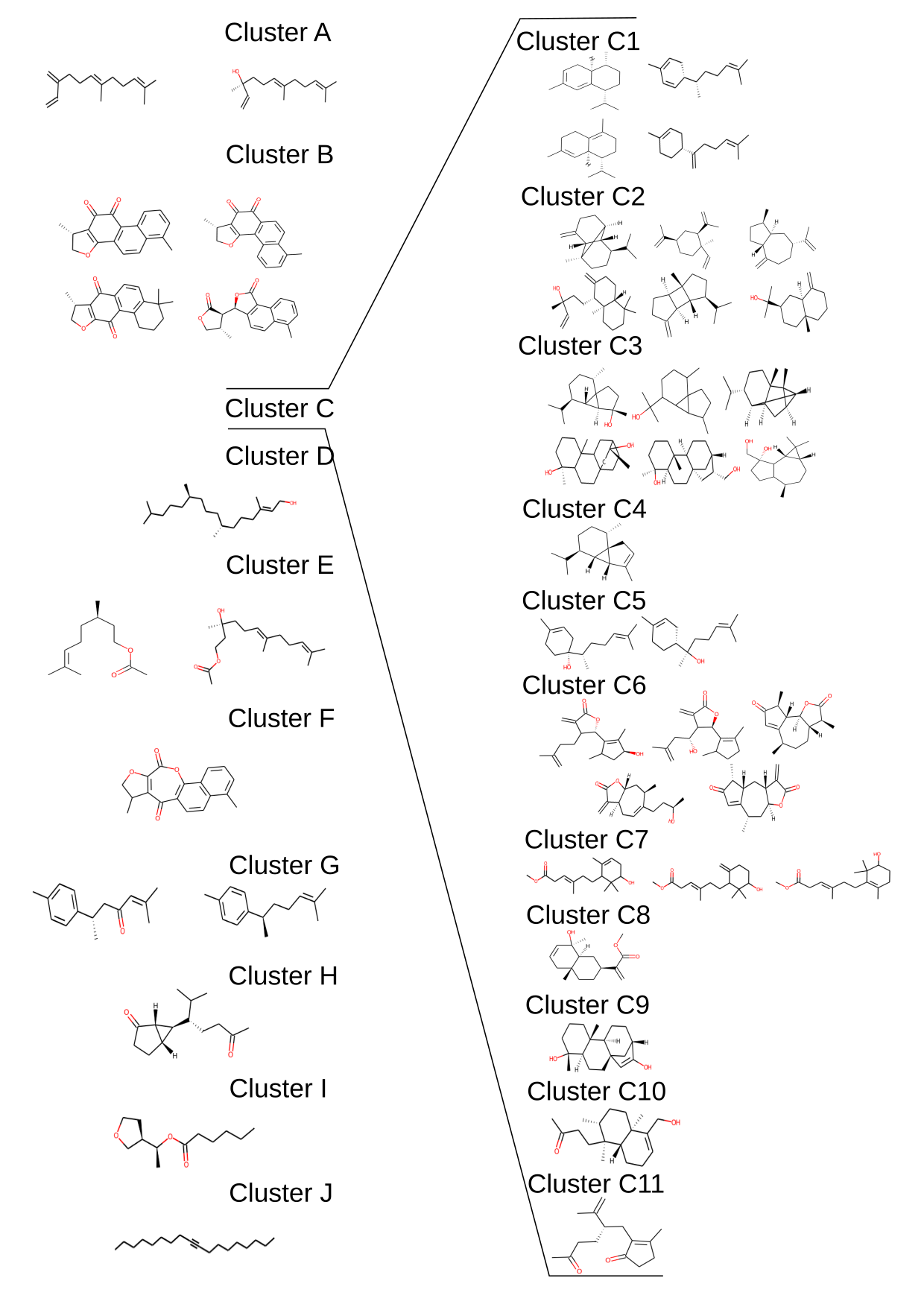

Supplement: Supplementary file 1 — Supplementary Information 1. [file 41598_2023_29981_MOESM1_ESM.zip › FigureS14.png]

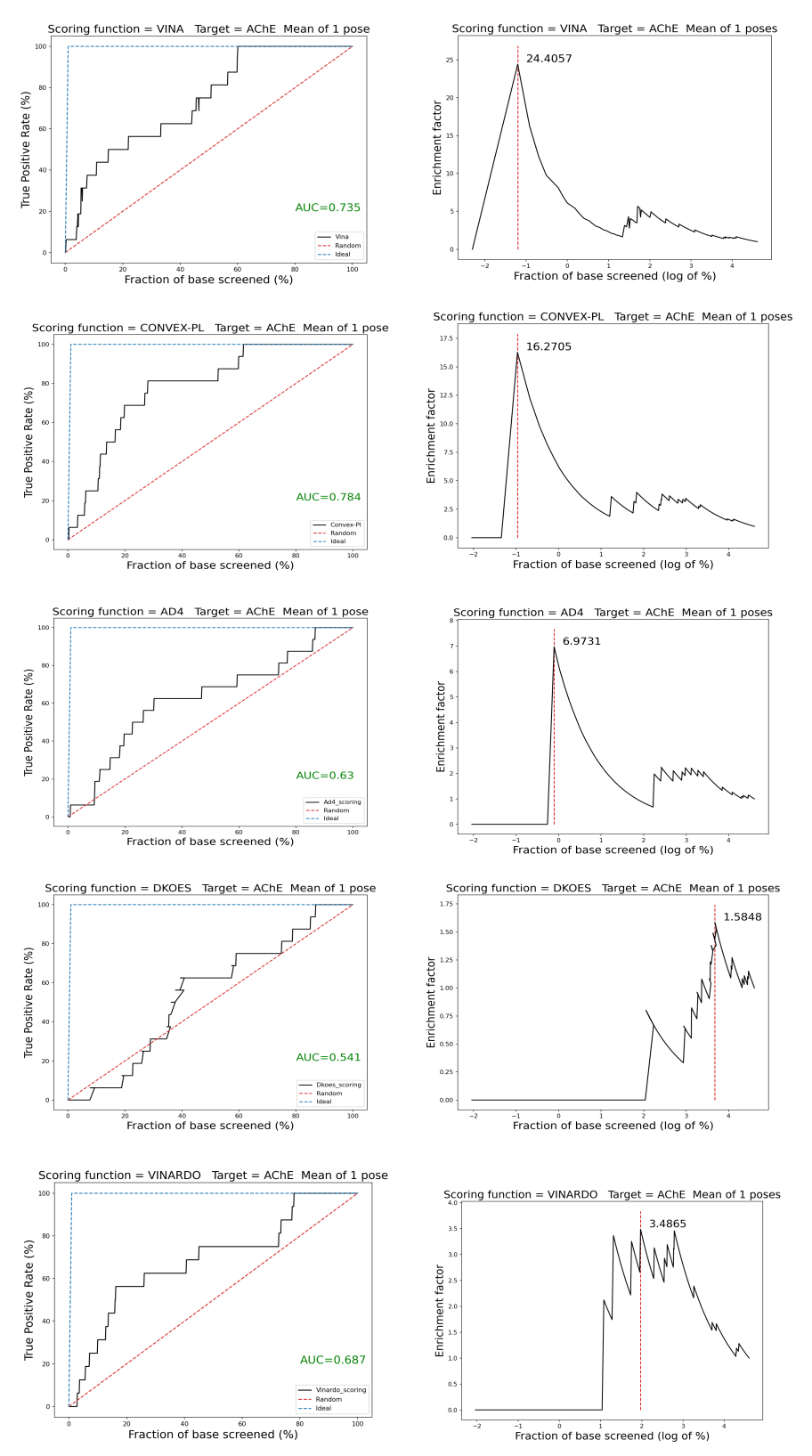

Supplement: Supplementary file 1 — Supplementary Information 1. [file 41598_2023_29981_MOESM1_ESM.zip › FigureS15_A.png]

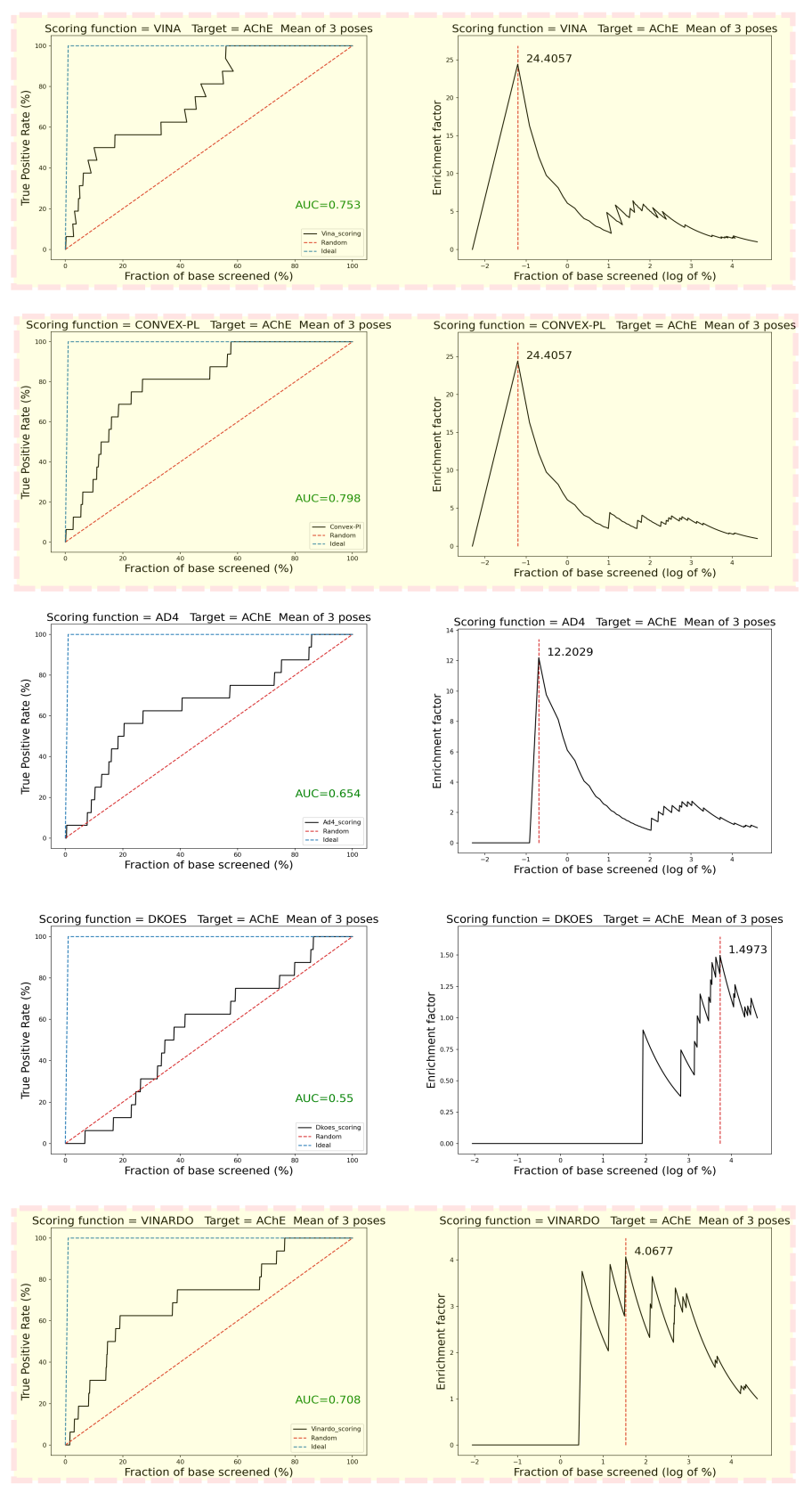

Supplement: Supplementary file 1 — Supplementary Information 1. [file 41598_2023_29981_MOESM1_ESM.zip › FigureS15_B.png]

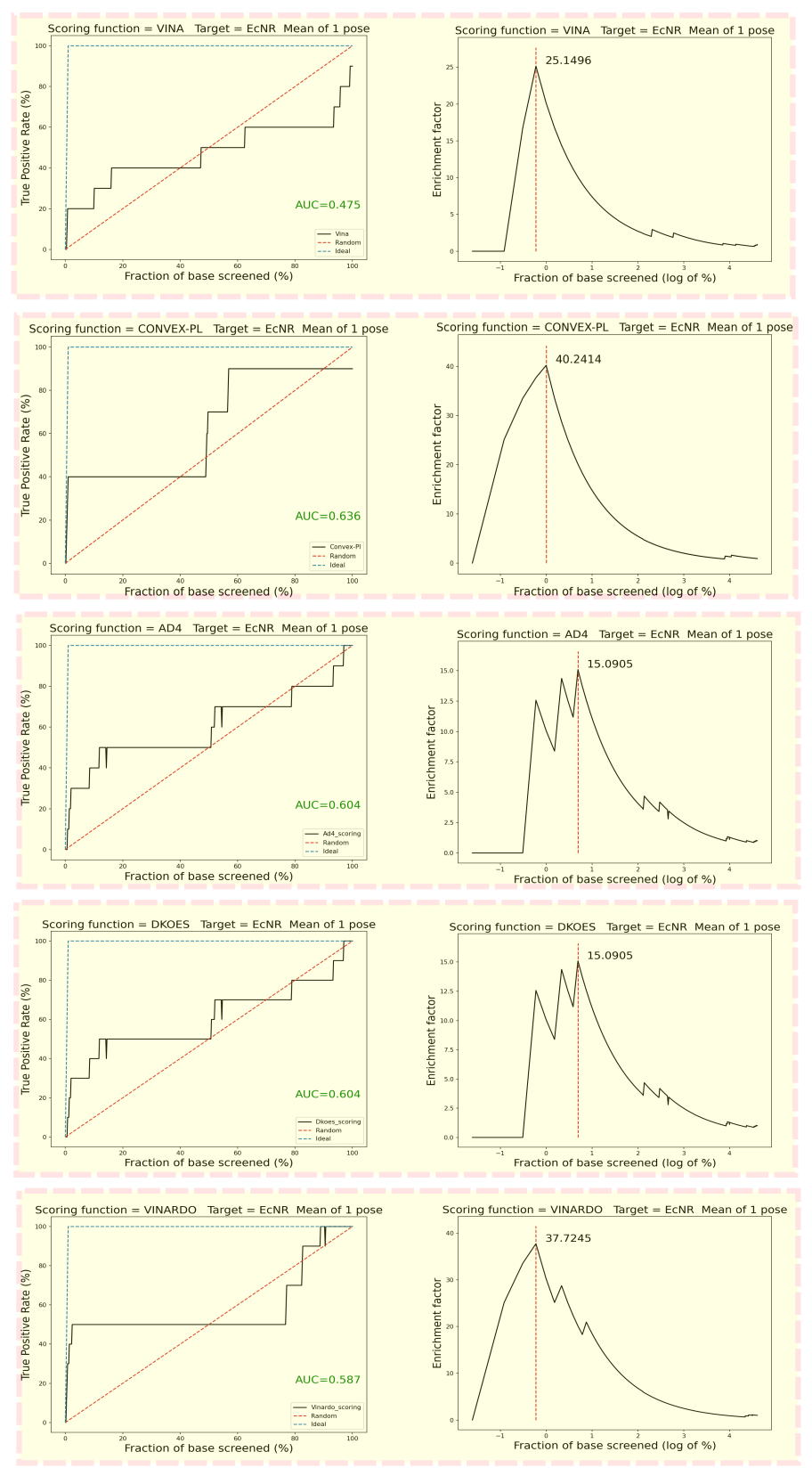

Supplement: Supplementary file 1 — Supplementary Information 1. [file 41598_2023_29981_MOESM1_ESM.zip › FigureS16_A.png]

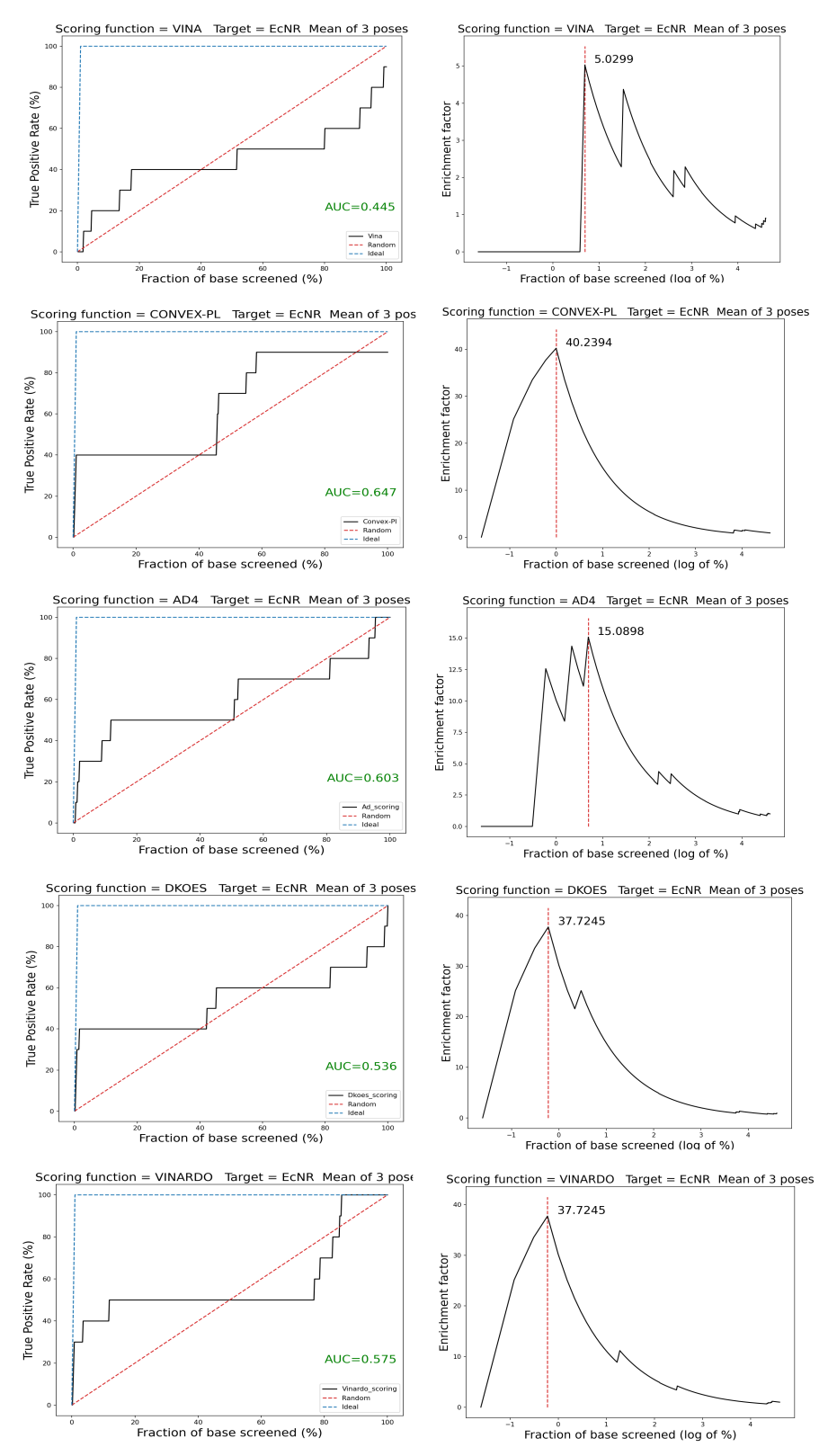

Supplement: Supplementary file 1 — Supplementary Information 1. [file 41598_2023_29981_MOESM1_ESM.zip › FigureS16_B.png]

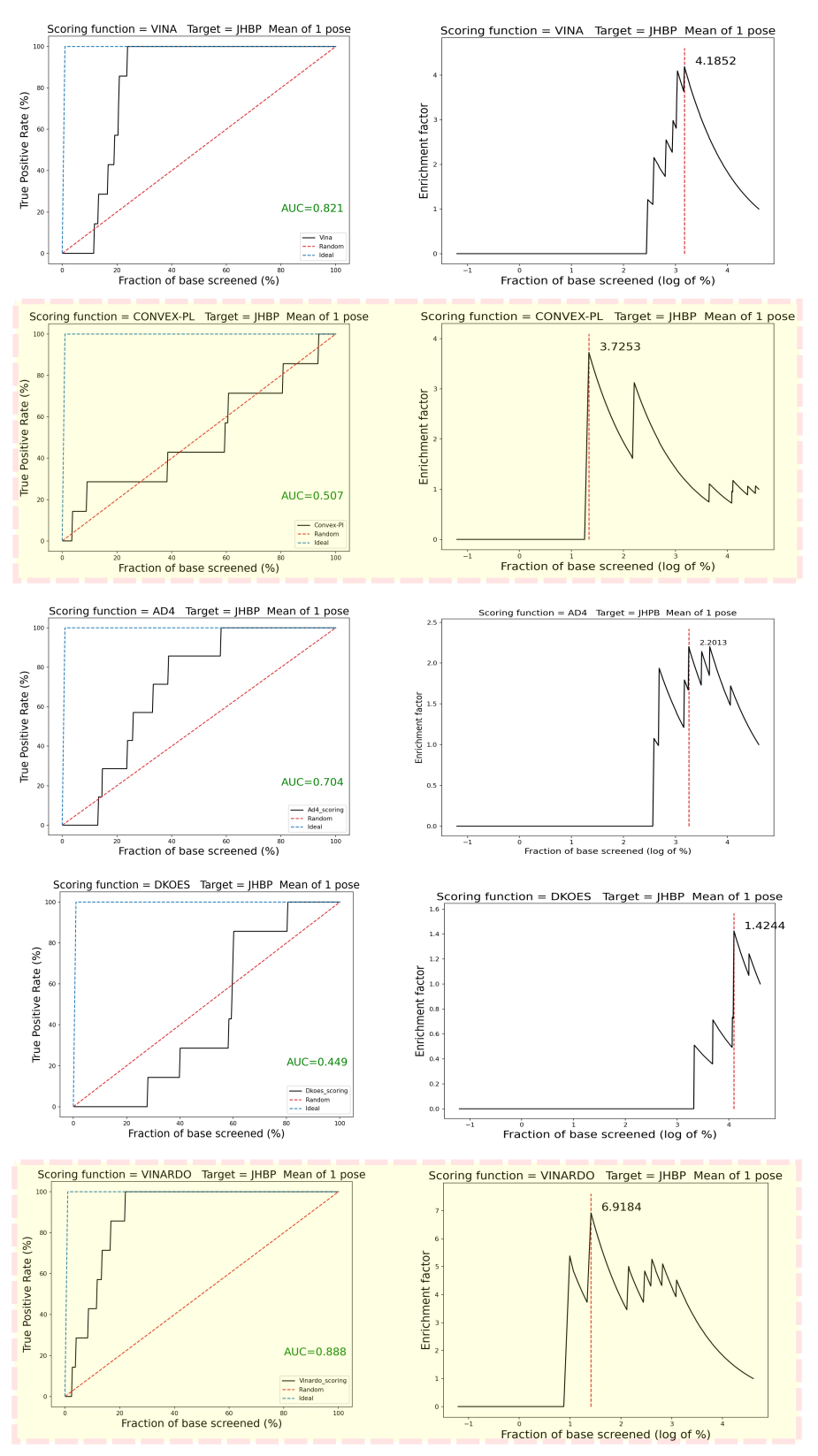

Supplement: Supplementary file 1 — Supplementary Information 1. [file 41598_2023_29981_MOESM1_ESM.zip › FigureS17_A.png]

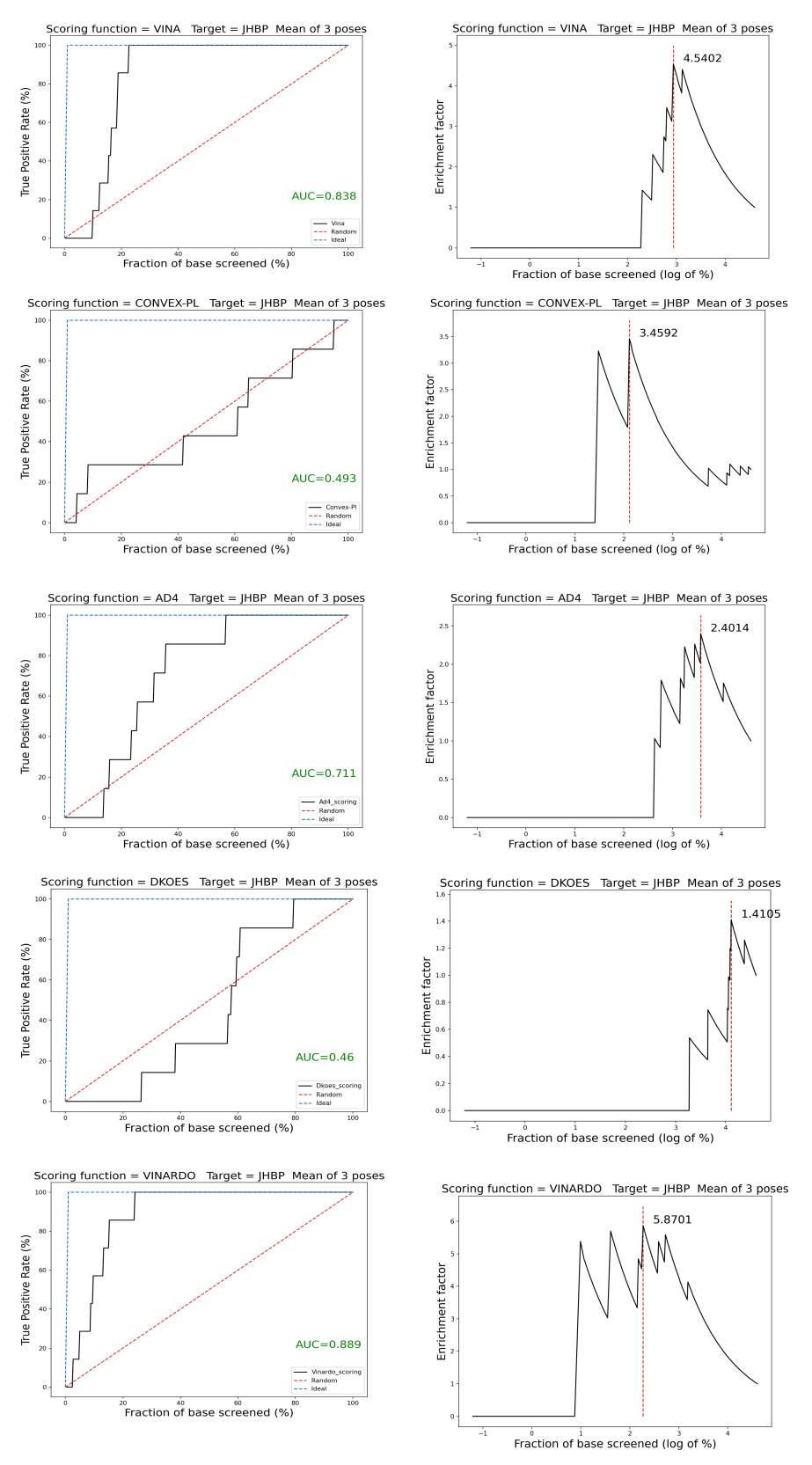

Supplement: Supplementary file 1 — Supplementary Information 1. [file 41598_2023_29981_MOESM1_ESM.zip › FigureS17_B.png]

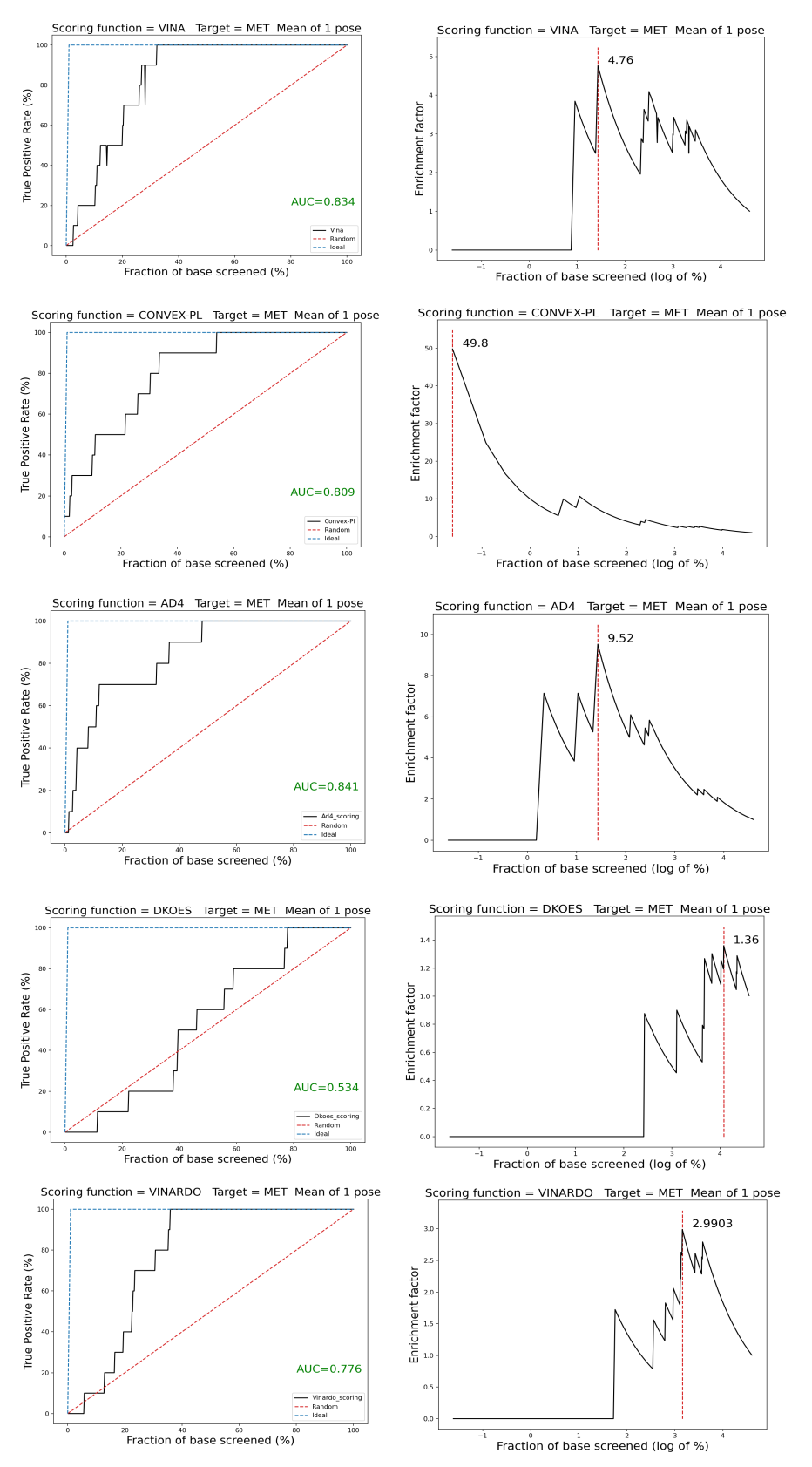

Supplement: Supplementary file 1 — Supplementary Information 1. [file 41598_2023_29981_MOESM1_ESM.zip › FigureS18_A.png]

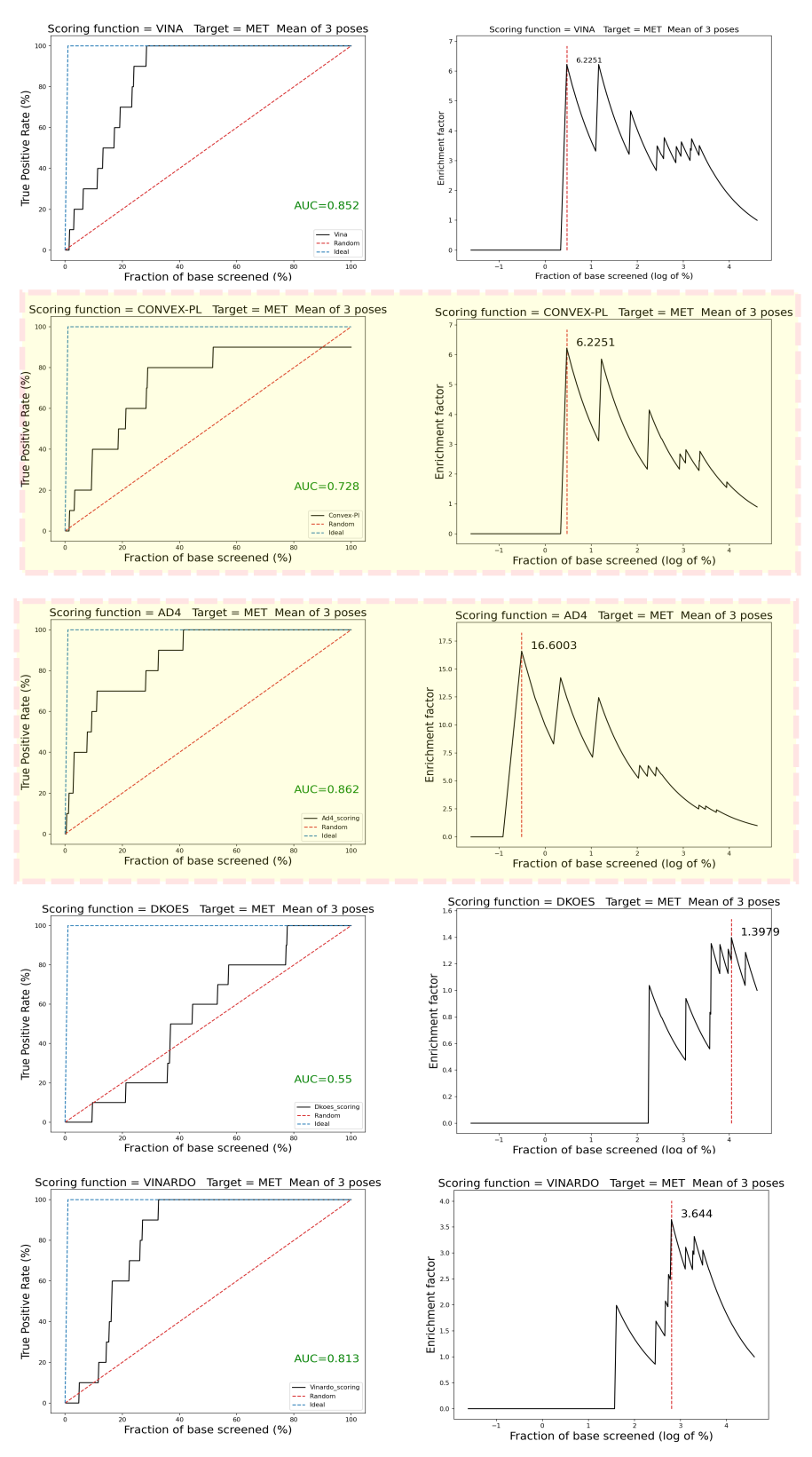

Supplement: Supplementary file 1 — Supplementary Information 1. [file 41598_2023_29981_MOESM1_ESM.zip › FigureS18_B.png]

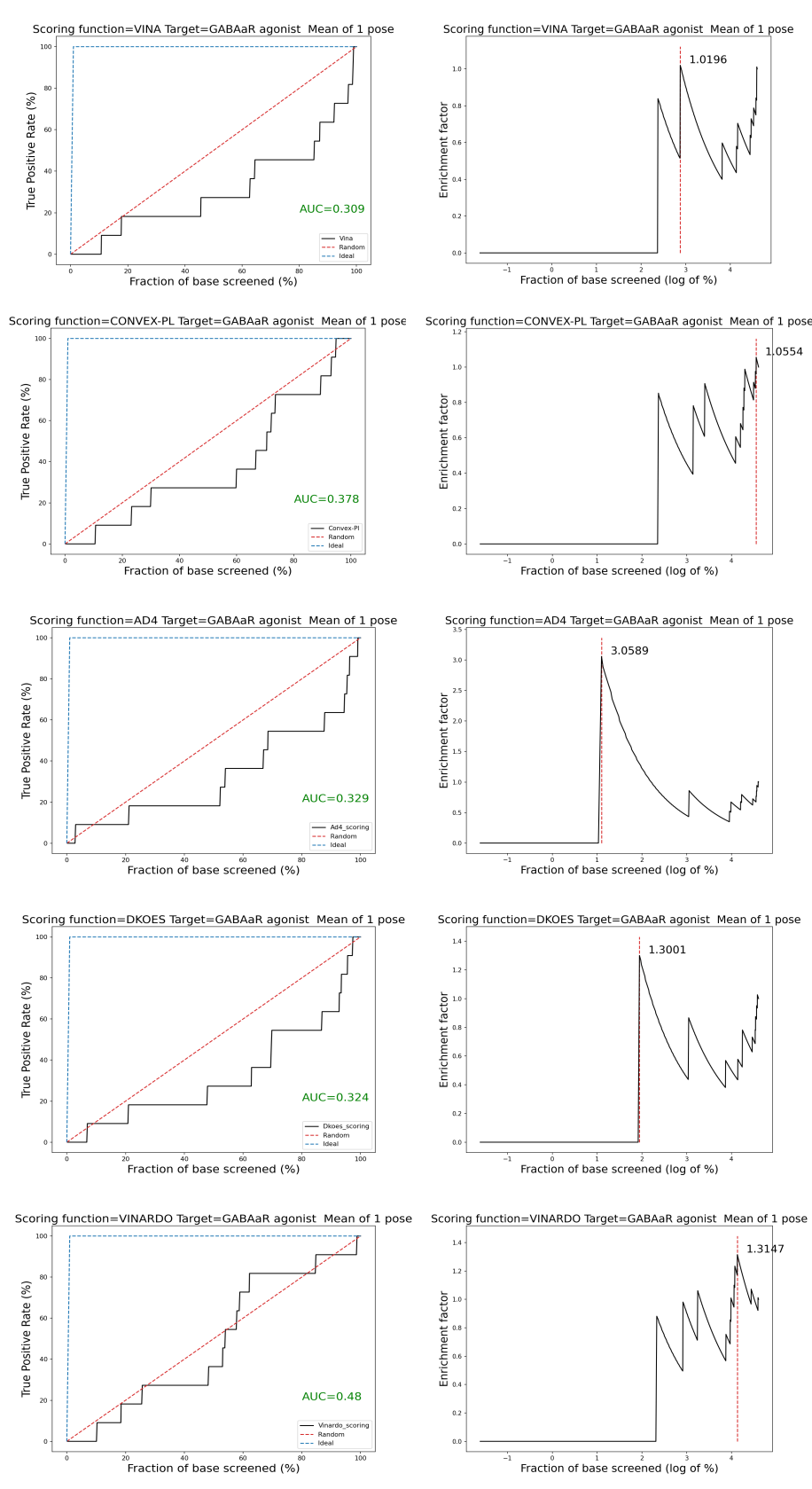

Supplement: Supplementary file 1 — Supplementary Information 1. [file 41598_2023_29981_MOESM1_ESM.zip › FigureS19_A.png]

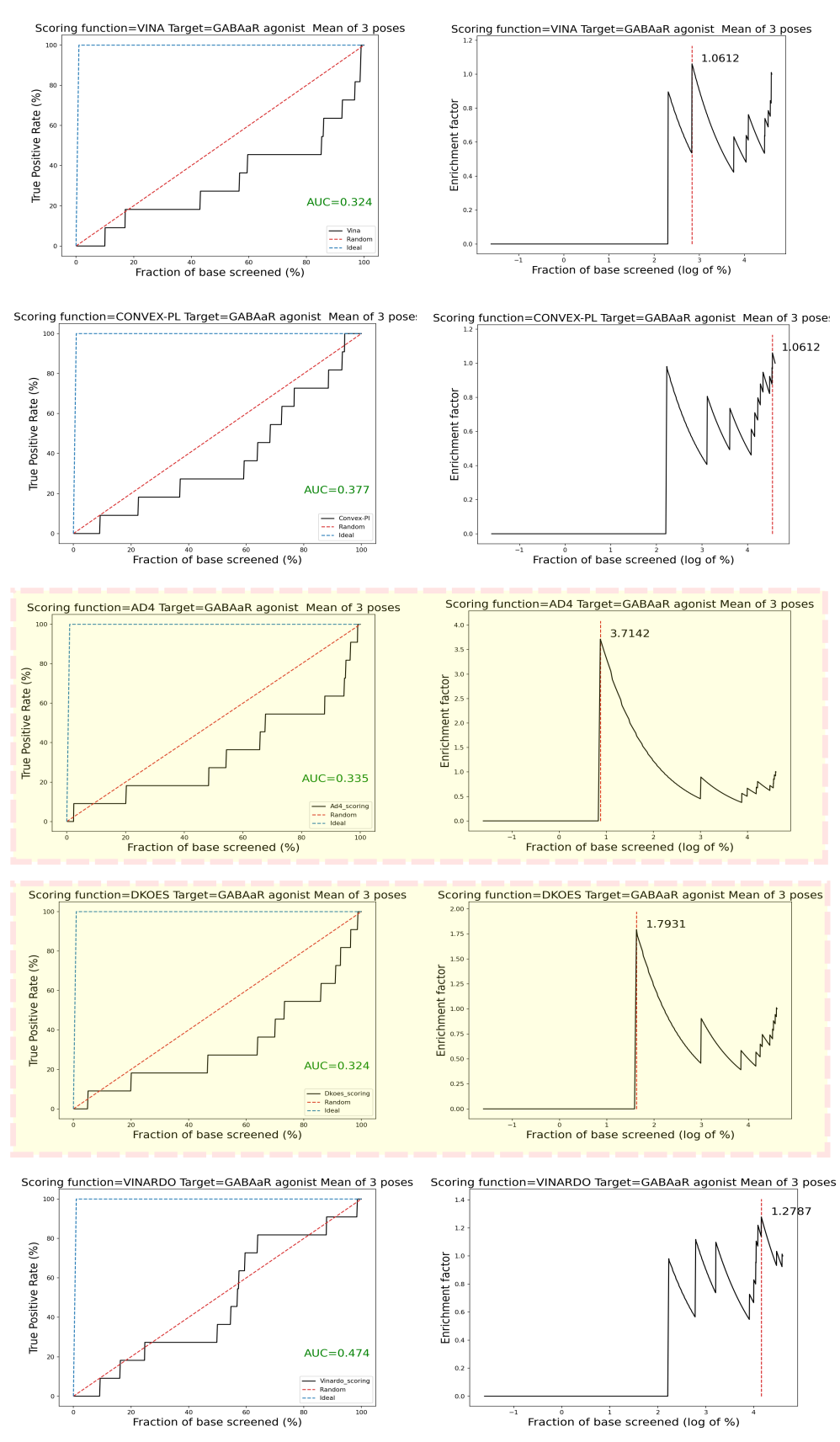

Supplement: Supplementary file 1 — Supplementary Information 1. [file 41598_2023_29981_MOESM1_ESM.zip › FigureS19_B.png]

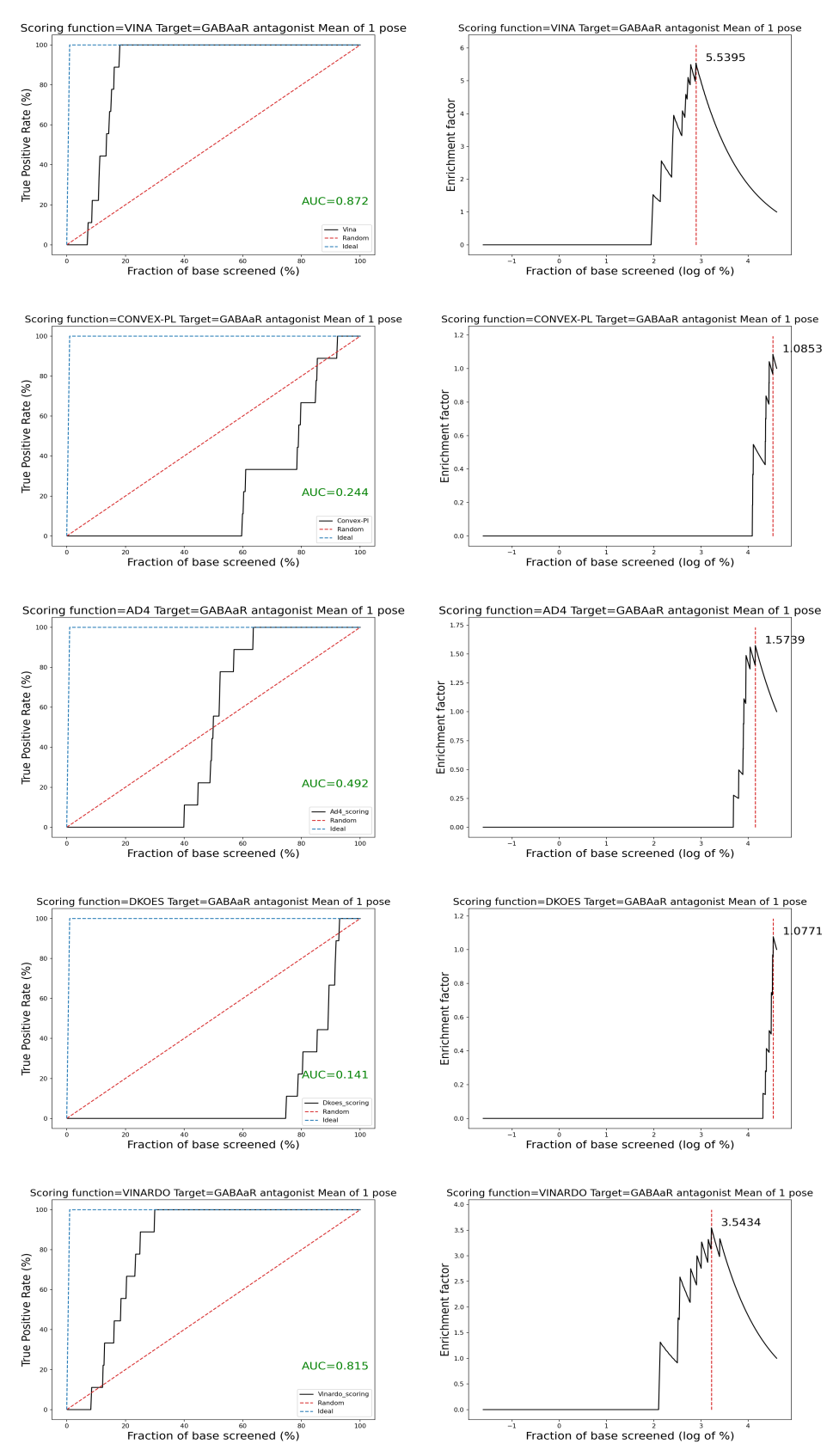

Supplement: Supplementary file 1 — Supplementary Information 1. [file 41598_2023_29981_MOESM1_ESM.zip › FigureS20_A.png]

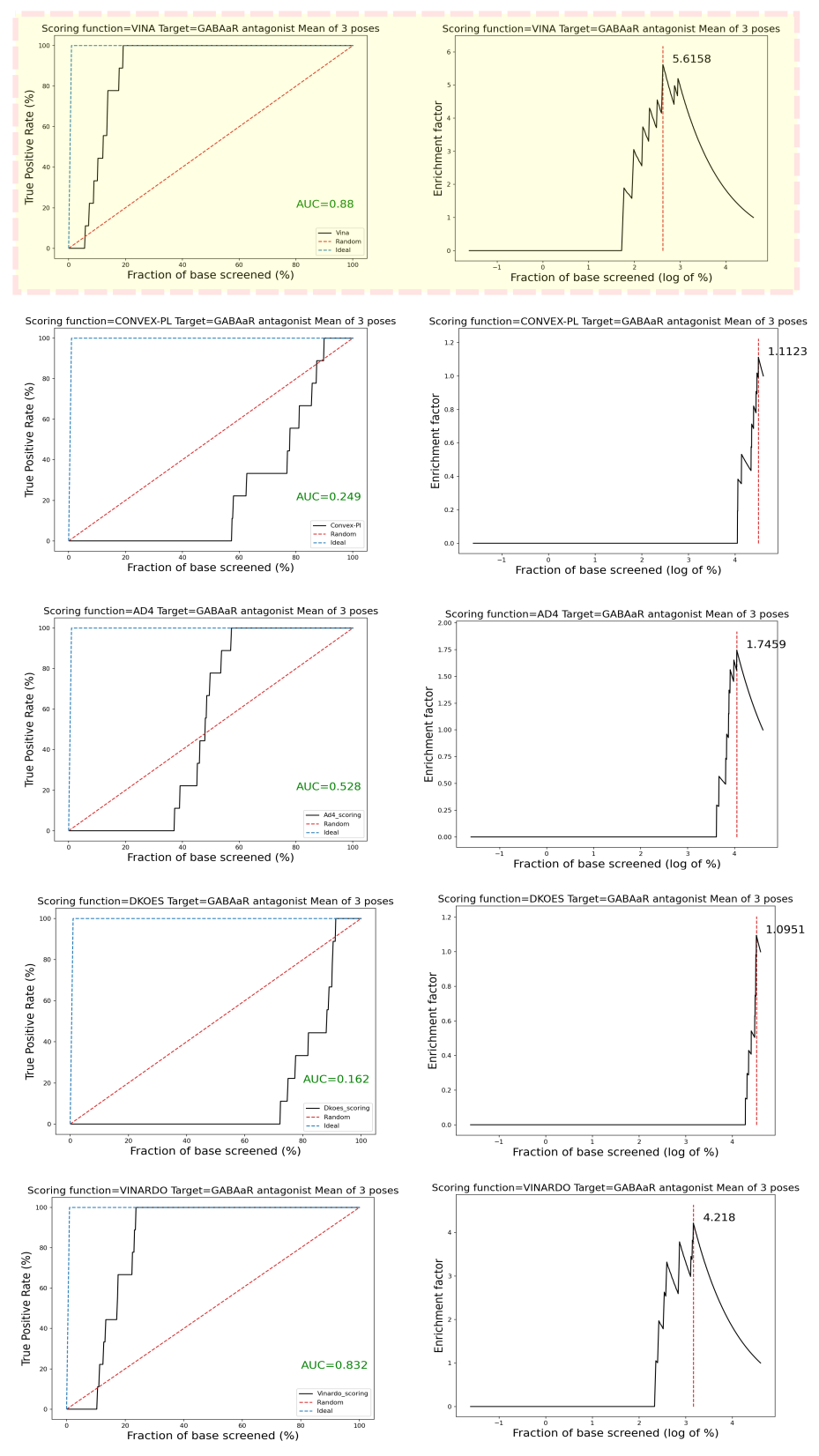

Supplement: Supplementary file 1 — Supplementary Information 1. [file 41598_2023_29981_MOESM1_ESM.zip › FigureS20_B.png]

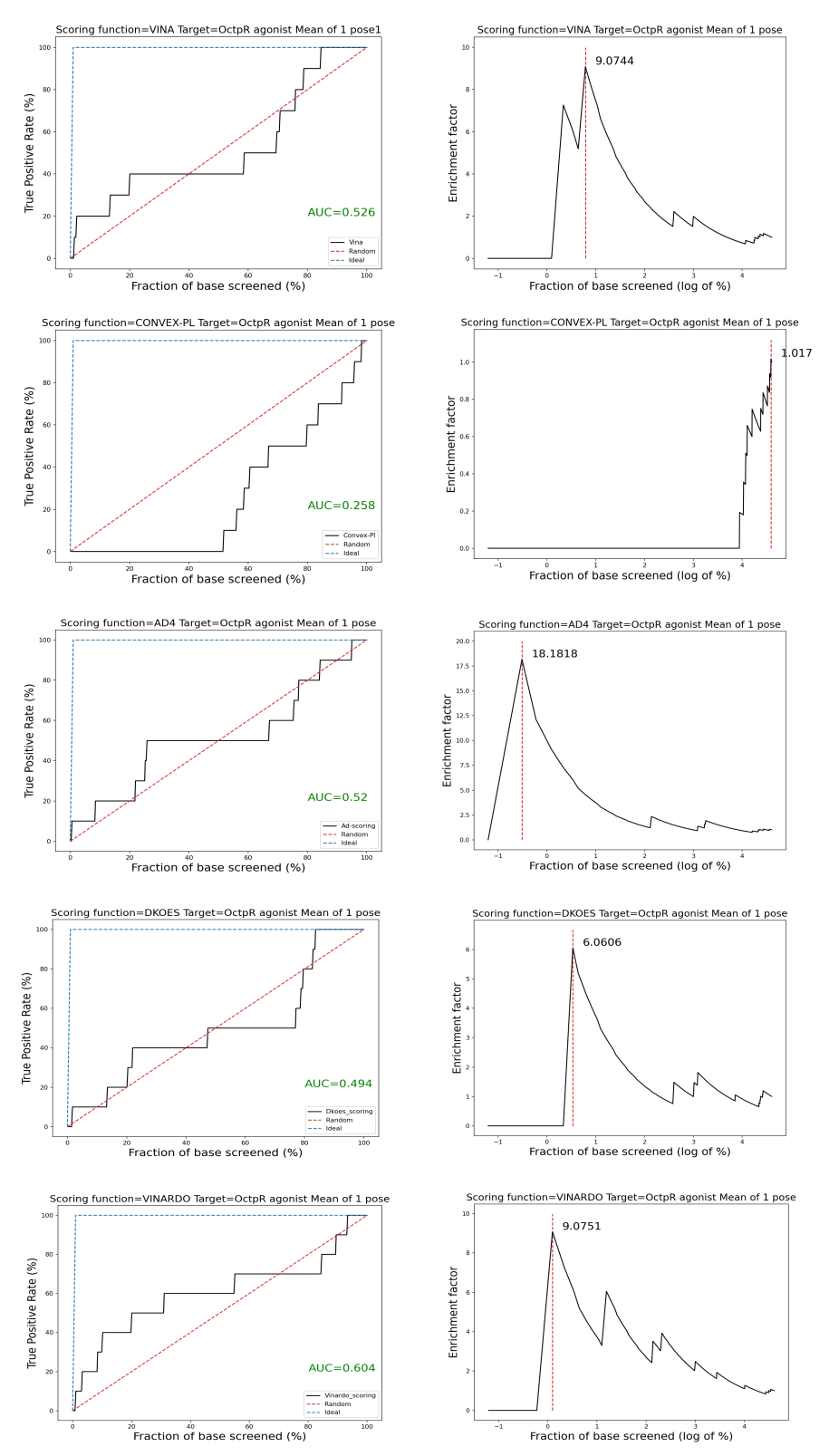

Supplement: Supplementary file 1 — Supplementary Information 1. [file 41598_2023_29981_MOESM1_ESM.zip › FigureS21_A.png]

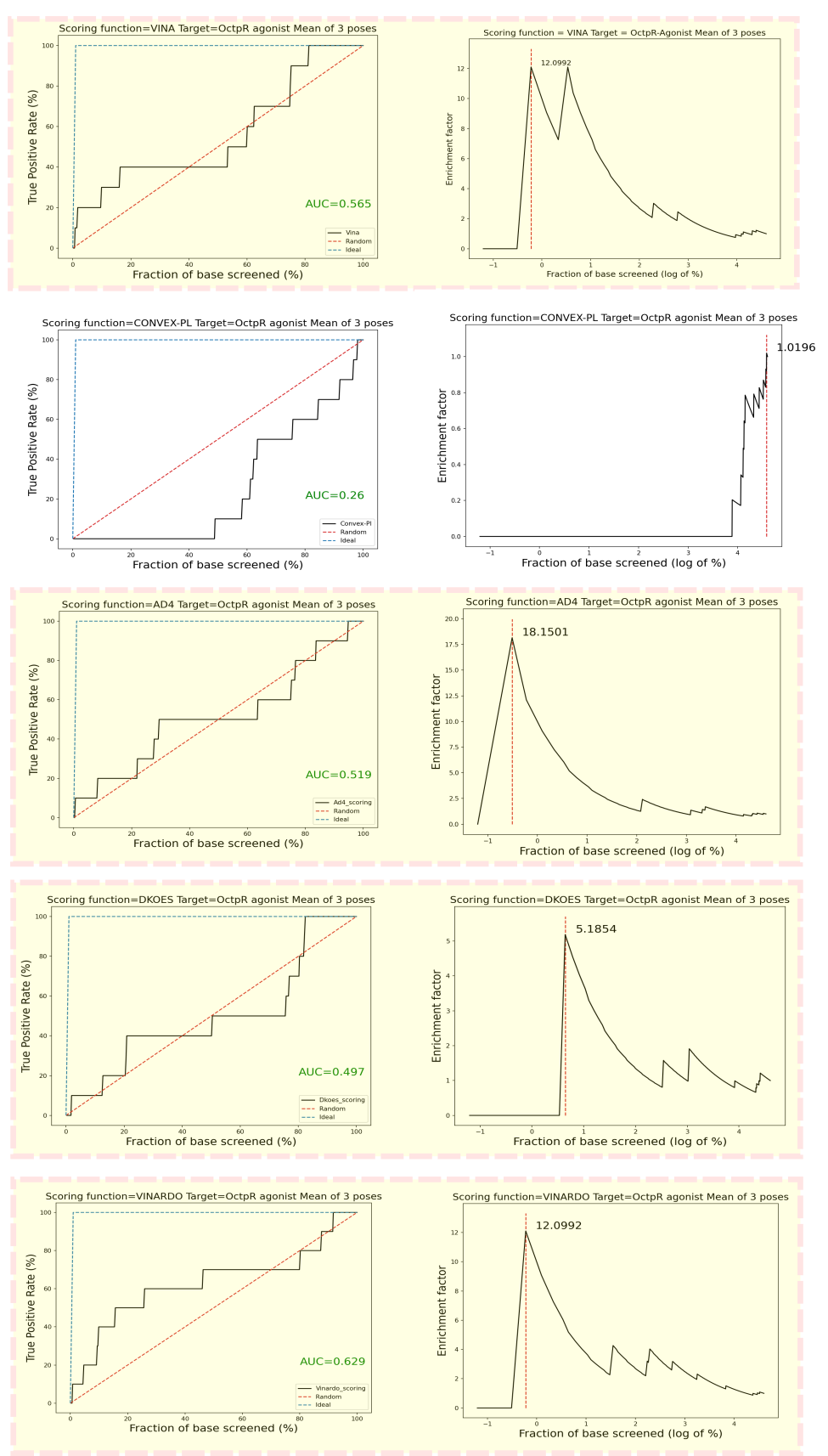

Supplement: Supplementary file 1 — Supplementary Information 1. [file 41598_2023_29981_MOESM1_ESM.zip › FigureS21_B.png]

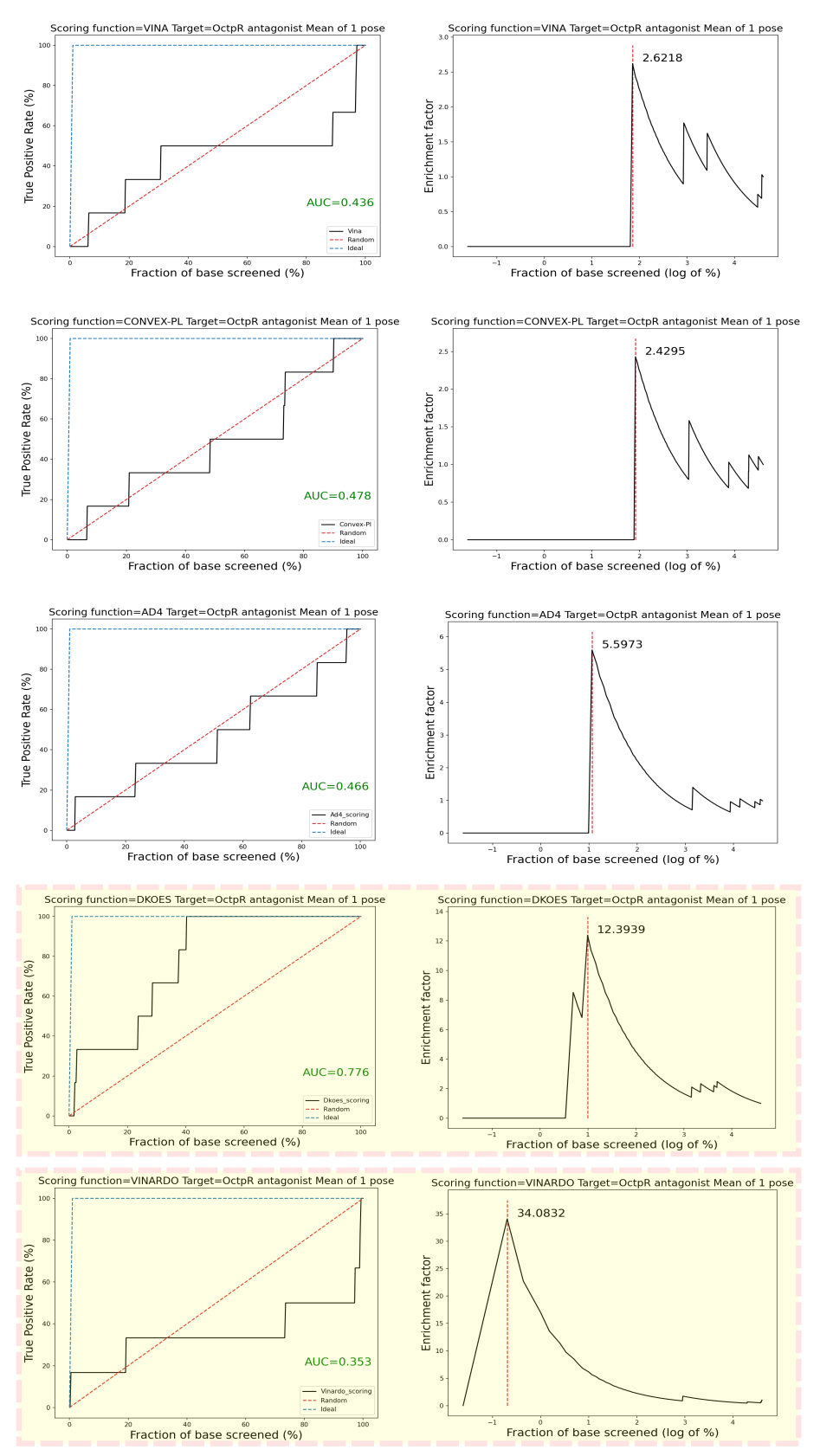

Supplement: Supplementary file 1 — Supplementary Information 1. [file 41598_2023_29981_MOESM1_ESM.zip › FigureS22_A.png]

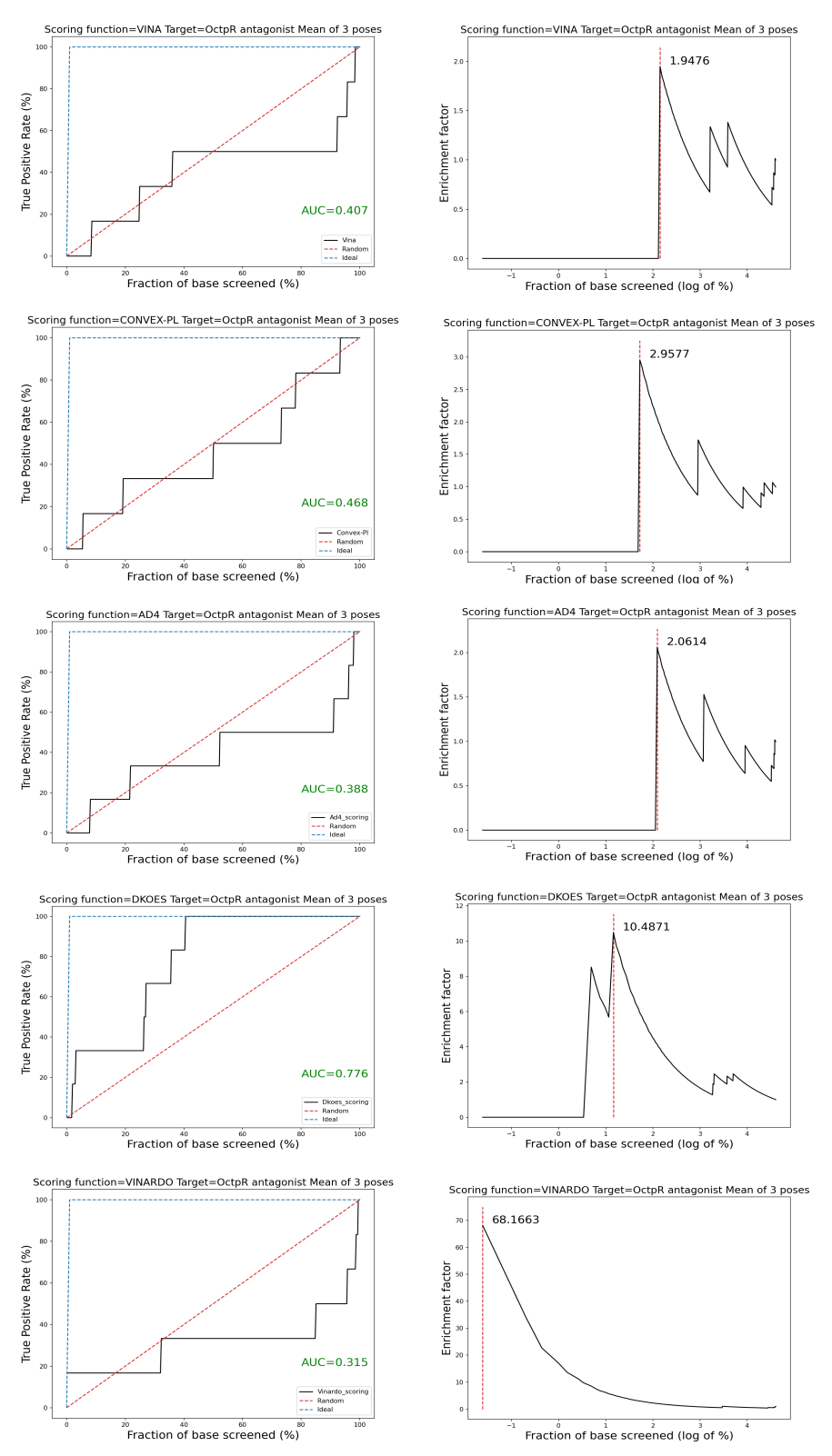

Supplement: Supplementary file 1 — Supplementary Information 1. [file 41598_2023_29981_MOESM1_ESM.zip › FigureS22_B.png]

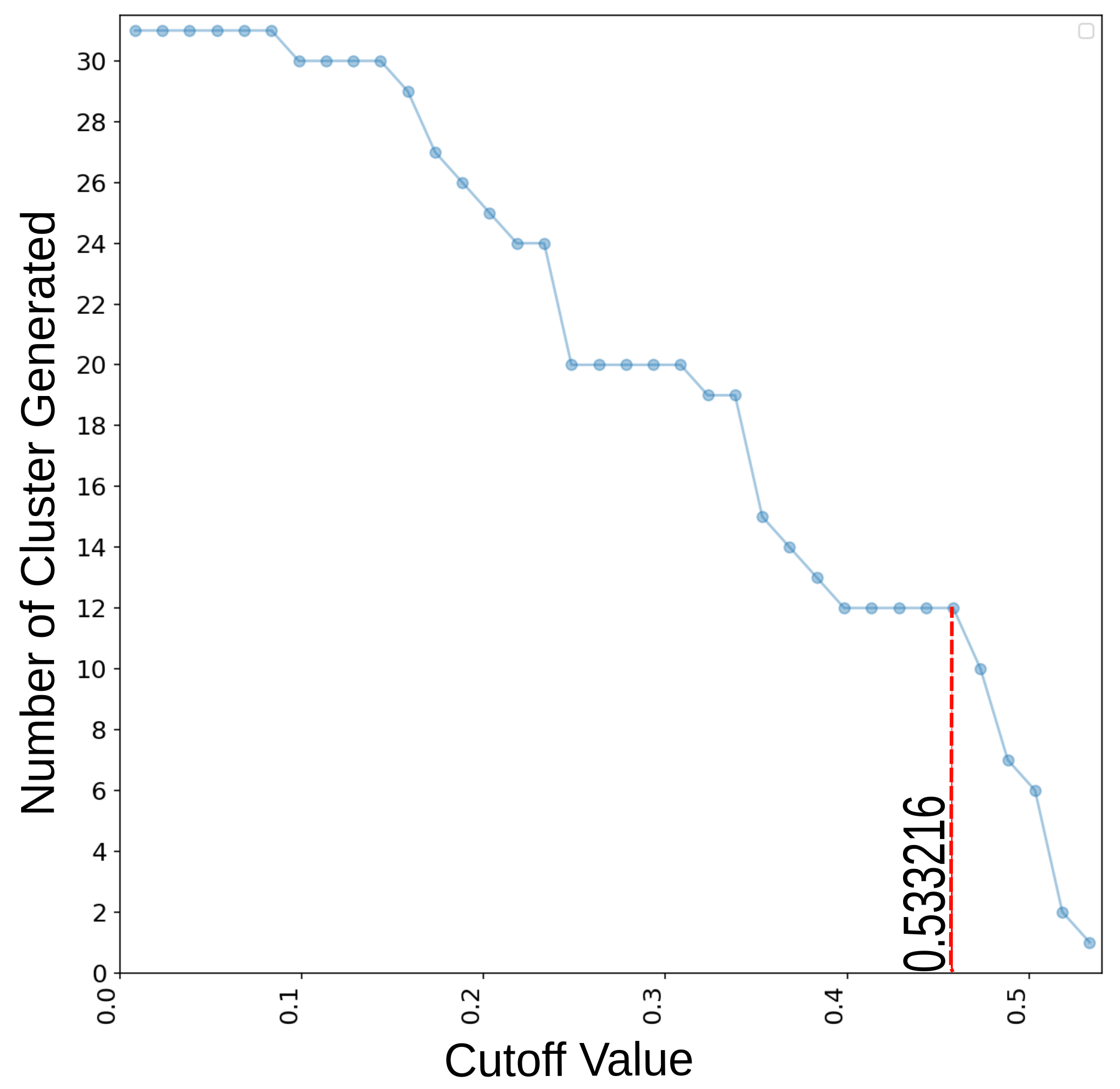

Supplement: Supplementary file 1 — Supplementary Information 1. [file 41598_2023_29981_MOESM1_ESM.zip › FigureS23.png]

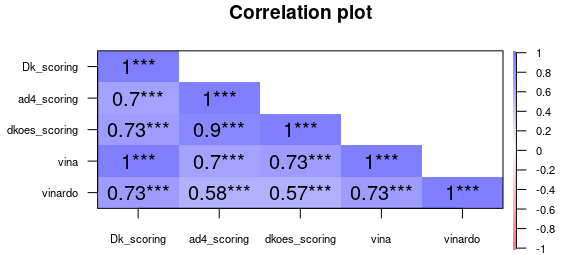

Supplement: Supplementary file 1 — Supplementary Information 1. [file 41598_2023_29981_MOESM1_ESM.zip › FigureS24.png]
