# Supplementary material for: Elucidating the molecular mechanisms of essential oils' insecticidal action using a novel cheminformatics protocol
Source: Sci Rep. 2023 Mar 21;13:4598. doi: 10.1038/s41598-023-29981-3 (PMC10028760; doi:10.1038/s41598-023-29981-3)
Supplement: Supplementary file 3 — Supplementary Information 3. [file 41598_2023_29981_MOESM3_ESM.docx]

#

#

#

#

**Supplementary Materials:**

Elucidating the molecular mechanisms of essential oils' insecticidal action using a novel chemoinformatics protocol

Eduardo José Azevedo Corrêa^1,2^, Frederico Chaves Carvalho^3^, Júlia Assunção de Castro Oliveira^4^, Suzan Kelly Vilela Bertolucci^4^, Marcus Tullius Scotti^5^, Carlos Henrique Silveira^6^, Fabiana Costa Guedes^6^, Júlio Onésio Ferreira Melo^7^, Raquel Cardoso de Melo-Minardi^3^, and *Leonardo Henrique França de Lima^7^.

***** Corresponding author

E-mail: [leofrancalima@ufsj.edu.br](mailto:leofrancalima@ufsj.edu.br)

1 Multicenter Program in Postgraduate in Biochemistry and Molecular Biology, Federal University of São João del-Rei, Campus Divinópolis, City Divinópolis-MG, Brazil.

2 Minas Gerais Agricultural Research Company (EPAMIG), City Pitangui-MG, Brazil.

3 Department of Computer Science, Institute of Exact Sciences - ICEx, Federal University of Minas Gerais, Campus Belo Horizonte, City Belo Horizonte-MG, Brazil.

4 Laboratory of Phytochemistry and Medicinal Plants, Department of Agriculture, Federal University of Lavras, City Lavras-MG, Brazil.

5 Exact and Nature Sciences Center, Chemistry Department, Federal University of Paraiba, Campus I, City João Pessoa-PB, Brazil.

6 Federal University of Itajubá, Campus Itabira, City Itabira-MG, Brazil.

7 Department of Exact and Biological Sciences, Sete Lagoas Campus, Federal University of São João Del-Rei, City Sete Lagoas-MG, Brazil.

**Figure S1. Analysis result using Mendeley Desktop of scientific literature surveyed.**


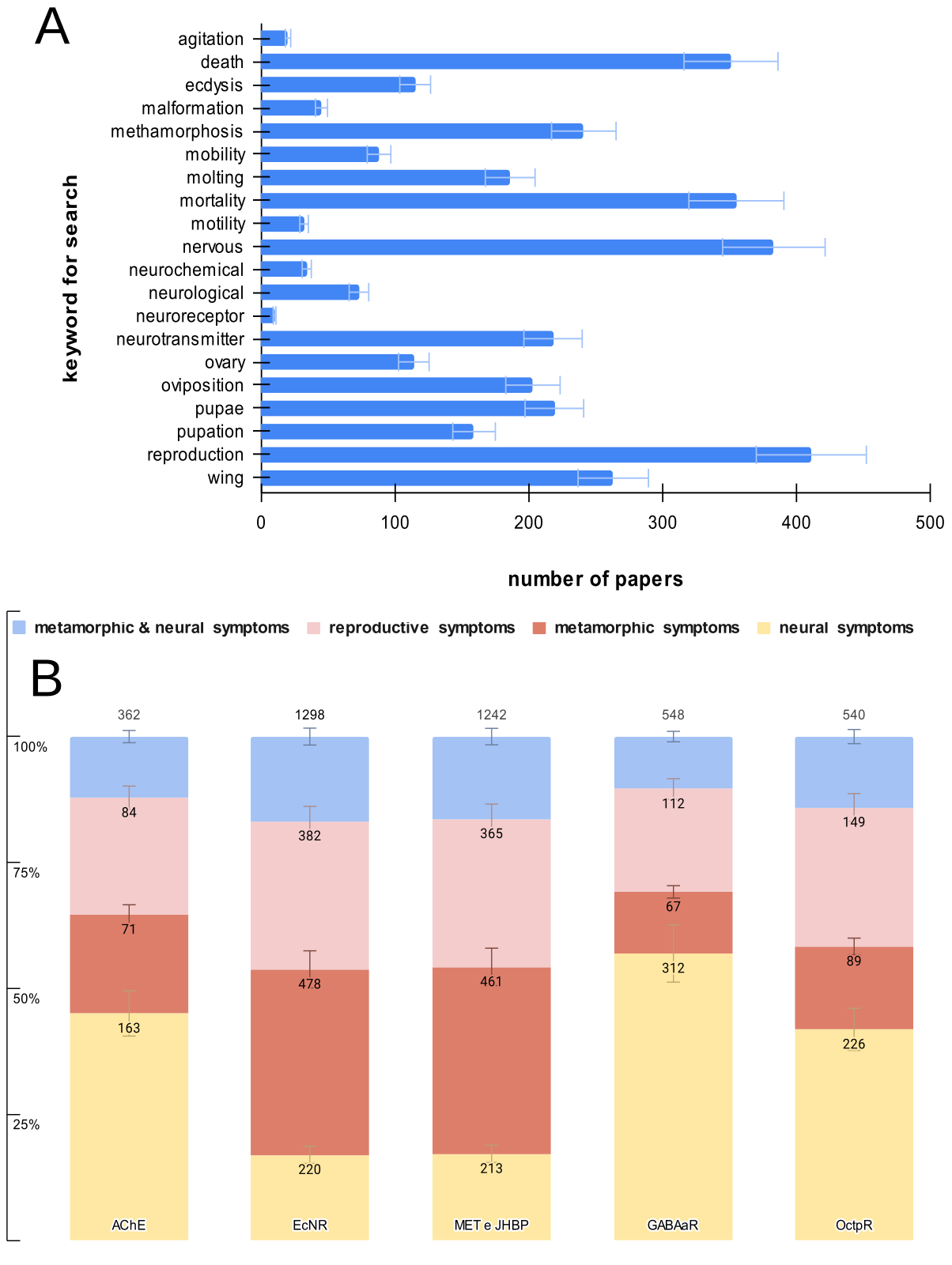


**Table S1. Data information about the Protein Homology Modelling Protocol used**

|  | **AChE** | **EcNR Agonist** | **JHBP** | **MET** | **GABAaR**  **Agonist** | **GABAaR**  **Antagonist** | **OctpR Agonist** | | **OctpR Antagonist** | |
| --- | --- | --- | --- | --- | --- | --- | --- | --- | --- | --- |
| Organism sequence | *Cochliomyia hominivorax* | *Lucilia cuprina* | *Aedes aegypti* | *Lucilia cuprina* | *Lucilia cuprina* | *Lucilia cuprina* | *Lucilia cuprina* | | *Lucilia cuprina* | |
| Amino Acids sequence ID | D5FQ11 (Uniprot) | A0A0L0BTL1 (Uniprot) | *Not Applicable | A0A0L0C1U9 (Uniprot) | O17145 (Uniprot) | O17145 (Uniprot) | XP_023298056.1 (GenBank) | | XP_023298056.1 (GenBank) | |
| Template PDB ID | 1DX4 Chain A | 4OZT Chain E | 5V13 Chain A | 3F1P Chain A | 4COF Chains A and B | 4COF Chains A and B | 6K42 Chain R | 7DHI Chain R | 5D6L Chain A | 6PRZ Chain A |
| template resolution | 2.70 | 2.70 | 1.84 | 1.17 | 2.97 | 2.97 | 4.10 | 3.26 | 3.20 | 2.80 |
| Cristalographic Ligand | Tetrahydroacridine | Ponasterone A | Juvenile Hormone III |  | Benzamidine | Benzamidine | Dexmedetomidine | Salbutamol | (S)-Carazolol | (S)-Alprenolol |
| Sequence Indentity (%) | 88.2 | 67.9 | *Not Applicable | 22.0 | 30.6 | 30.6 | 45.7 | 42.7 | 42.7 | 42.7 |
| Sequence Coverage (%) | 77.7 | 26.4 | *Not Applicable | 70.5 | 64.0 | 64.0 | 36.0 | 46.0 | 62.0 | 60.0 |
| Reference | [**1**, **2**] | [**3**, **4**] | [**5**] | [**3**, **6**] | [**7**] | [**7**] | [**8**] | [**9**] | [**11**] | [**12**] |

* The same protein homology-modelling procedure are adopted to juvenile hormone binding protein (JHBP), however the *Aedes aegypti* template 5V13 is at the same time the better target template and the more closer dipteran JHBP protein sequence to the Calliphoridae diptera. Therefore, to not alter the protein homology-modeling protocol in this unique case, we used the 5V13 fasta sequence and modeled the tertiary protein structure using the same protocols used for the other blowfly protein targets.

## **Protein Homology Modelling**

**Table S2. Reference Table of Quality Parameters Indicators for protein models generated**

|  |  |  |  |  |  |  |  | **Ramachandran plot** | | |  |  |
| --- | --- | --- | --- | --- | --- | --- | --- | --- | --- | --- | --- | --- |
| **Homoly modeling platform** | **GA341** | **DOPE** | **z-DOPE** | **molpdf** | **ProQ2** | **GMQE** | **Z-QMEAN** | **Rfr** | **Raar** | **Outliers** | **Z-Score** | **RMSD** |
| **Chimera USC** | 1.0 (native-like) | X | - 1 >  (native-like) | lowest value | X | X | X | Higher values | Higher values | lower values | More negative | lower is better |
| **Modeller** | 1.0 (native-like) | lowest value | _ | lowest value | X | X | X | Higher values | Higher values | lower values | More negative | lower is better |
| **Swiss-Model** | X | X | X | X | X | 1.0 (native-like) | 0.0 is native  < -4 is a poor model | Higher values | Higher values | lower values | More negative value | lower is better |
| **Phyre2** | X | X | X | X | Higher predicted global quality is better | X | X | Higher values | Higher values | lower values | More negative | lower is better |

**GA341** is optimized discriminant function evolved by the genetic algorithm is a nonlinear combination of model features [**13**] exhibits several interesting properties. It is restricted between 0 and 1, where 1 assumes native-like conformation and 0 is non-native.

**DOPE** (Discrete Optimized Protein Energy) potential is the sum of interactions between pairs of atoms, it can be decomposed into a score per residue that can be generated for each model to give a reference in choice of the best DOPE score [**14**]. This is a statistical potential optimized for model assessment [**15**]. The model that returns the lowest DOPE score is selected.

**Z-DOPE** It is a Z-scored of DOPE. Positive scores indicate poor models, while scores lower than -1 are likely native-like proteins. The mean and standard deviation to normalize the DOPE score for a protein is estimated from its sequence. The mean is estimated by a weighted sum of protein composition over the 20 standard amino acid residue types. The weight corresponds to the change in the score by inserting a specific type of amino acid residue [**16**]

**molpdf** molecular PDF (molpdf) scoring function is the sum of all restraints in protein structure. This score is used to rank the models calculated from the same alignment. The best-generated model can be chosen by selecting the model with the lowest molpdf value [**17**]. **ProQ2** is an algorithm that uses support vector machines (SVM) to predict protein models' local and global quality**.** The quality of a protein model is measured by combining structural and sequence-based features calculated from the model [**18**].

**GMQE** (Global Model Quality Estimate) is a quality estimate that combines target-template alignment and the template structure properties using a multilayer perceptron trained to predict the lDDT score of the resulting model. GMQE gives the overall model quality measurement between 0 and 1, higher numbers indicate higher model quality [**19**, **20**].

**Z-QMEAN** is based on the QMEAN score. They are compared with experimentally determined structures of similar size using Z-scores and determine how many standard deviations from the mean of the model score in the score distribution of a large set of experimentally determined structures. A z-QMEAN of 0.0 reflects a "native-like," while a Z-QMEAN below -4.0 indicates a model with low quality [**19**, **21**].

**Rfr** Residues in the most favored region. A higher number of residues in the favored region is better [**22**]

**Raar** = Residues in additional allowed regions. A higher number of residues in allowed regions is better [**22**]

**Outliers** = Ramachandran outliers. A lower number of residues as outlier indicate a high-quality model [**22**]

**Z-SCORE** (PROSA) z-score indicates the overall model quality based on the Cα positions and measures the total energy deviation taking into account the energy distribution of random conformations [**23**]. More negative values of the z-score correspond to better model quality [**24**].

**Table S3. Quality assessment of protein target model made using the platform Chimera.**

| **Pt** | **Sc** | **molpdf** | **GA341** | **z-DOPE** | **Ramachandran Plots (Molprobity)** | | | **Ramachandran Plots (Procheck)** | | | | **PROSA** | **RMSD** |
| --- | --- | --- | --- | --- | --- | --- | --- | --- | --- | --- | --- | --- | --- |
|  |  |  |  |  |  |  |  |  |  |  |  | **Z-Score** |  |
|  |  |  |  |  | **Rfr** | **Raar** | **Outliers** | **Rmfr** | **Raar** | **Rgar** | **Rdr** |  |  |
| AChE | Unique | 3137.96 | 1.00 | -1.216 | 94.05 | 99.3 | 0.70 | 90.2 | 8.4 | 1.0 | 0.4 | -10.31 | 0.129 |
| EcNR | Agonist | 1122.14 | 1.00 | -1.047 | 95.8 | 98.7 | 1.27 | 98.7 | 5.0 | 2.3 | 0.5 | -8.38 | 0.115 |
| JHBP | Unique | 33994.94 | 1.00 | -0.404 | 97.0 | 99.6 | 0.37 | 91.4 | 8.2 | 0.4 | 0.0 | -8.27 | 0.220 |
| MET | Unique | 40147.88 | 0.88 | -0.365 | 93.9 | 99.0 | 1.01 | 90.1 | 8.8 | 0.0 | 1.1 | -4.91 | 0.317 |
| GABAaR | Agonist | 3239.36 | 0.99 | -0.413 | 98.2 | 99.5 | 0.46 | 95.5 | 4.4 | 0.2 | 0.0 | -3.25 | 0.334 |
| GABAaR | Antagonist | 3239.36 | 0.99 | -0.413 | 98.2 | 99.5 | 0.46 | 95.5 | 4.4 | 0.2 | 0.0 | -3.25 | 0.334 |
| OctpR | Agonist | 1421.27 | 0.91 | -0.255 | 94.1 | 98.6 | 1.39 | 92.1 | 6.8 | 0.4 | 0.8 | -3.32 | 0.202 |
| OctpR | Agonist | 1556.63 | 0.99 | -0.132 | 95.8 | 99.3 | 0.70 | 93.6 | 6.0 | 0.4 | 0.0 | -3.78 | 0.235 |
| OctpR | Antagonist | 1064.25 | 1.00 | -0.532 | 95.8 | 99.3 | 0.70 | 93.6 | 5.3 | 0.8 | 0.4 | -4.07 | 0.123 |
| OctpR | Antagonist | 1199.53 | 0.99 | -0.596 | 96.5 | 99.3 | 0.70 | 95.5 | 3.4 | 0.4 | 0.8 | -4.04 | 0.186 |

**Pt** = Protein Target; **Sc** = Structure conformation; **Rfr** = Residues in most favored region; **Raar** = Residues in additional allowed regions; **Outliers** = Ramachandran outliers; **Rmfr** = Residues in most favored region; **Raar** = Residues in additional allowed regions; **Rgar** = Residues in generously allowed regions; **Rdr** = Residues in disallowed regions; **RMSD** = RMSD align to PBD template.

**Table S4. Quality assessment of protein target model made using the platform Modeller 9.25.**

| **Pt** | **Sc** | **molpdf** | **GA341** | **DOPE** | **Ramachandran Plots (Molprobity)** | | | **Ramachandran Plots (Procheck)** | | | | **PROSA** | **RMSD** |
| --- | --- | --- | --- | --- | --- | --- | --- | --- | --- | --- | --- | --- | --- |
|  |  |  |  |  |  |  |  |  |  |  |  | **Z-Score** |  |
|  |  |  |  |  | **Rfr** | **Raar** | **Outliers** | **Rmfr** | **Raar** | **Rgar** | **Rdr** |  |  |
| AChE | Unique | 4063.10 | 1.00 | -66902.83 | 94.3 | 99.5 | 0.52 | 90.5 | 8.0 | 1.0 | 0.4 | -10.29 | 0.162 |
| EcNR | Agonist | 1108.12 | 1.00 | -29002.91 | 97.0 | 100.0 | 0.00 | 94.1 | 5.0 | 0.9 | 0.0 | -8.11 | 0.100 |
| JHBP | Unique | 1307.97 | 1.00 | -33255.50 | 96.6 | 99.6 | 0.37 | 92.2 | 7.4 | 0.4 | 0.0 | -8.28 | 0.203 |
| MET | Unique | 990.55 | 0.91 | -11082.37 | 90.9 | 99.0 | 1.01 | 87.9 | 8.8 | 1.1 | 2.2 | -4.71 | 0.225 |
| GABAaR | Agonist | 3207.62 | NC | NC | 98.0 | 99.7 | 0.30 | 95.5 | 4.5 | 0.0 | 0.0 | -3.25 | 0.192 |
| GABAaR | Antagonist | 3207.62 | NC | NC | 98.0 | 99.7 | 0.30 | 95.5 | 4.5 | 0.0 | 0.0 | -3.25 | 0.192 |
| OctpR | Agonist | 1548.47 | 0.98 | -37246.41 | 93.7 | 98.6 | 1.39 | 92.1 | 7.2 | 0.4 | 0.4 | -3.56 | 0.197 |
| OctpR | Agonist | 1521.10 | 0.98 | -37542.42 | 95.1 | 99.0 | 1.05 | 91.3 | 7.5 | 0.8 | 0.4 | -3.57 | 0.331 |
| OctpR | Antagonist | 1164.34 | 0.99 | -39810.37 | 96.5 | 99.3 | 0.70 | 94.3 | 4.9 | 0.8 | 0.0 | -3.75 | 0.143 |
| OctpR | Antagonist | 1188.18 | 0.99 | -39890.62 | 96.2 | 99.0 | 1.05 | 94.7 | 3.4 | 0.4 | 1.5 | -3.81 | 0.150 |

**Pt** = Protein Target; **Sc** = Structure conformation; **Rfr** = Residues in most favored region; **Raar** = Residues in additional allowed regions; **Outliers** = Ramachandran outliers; **Rmfr** = Residues in most favored region; **Raar** = Residues in additional allowed regions; **Rgar** = Residues in generously allowed regions; **Rdr** = Residues in disallowed regions; **RMSD** = RMSD align to PBD template. **NC =** Modeller did not calculate this parameter because the model is a two-chain dimer. Therefore Modeller returns only model quality in the molpdf value.

**Table S5. Quality assessment of protein target model made using the platform Phyre2.**

| **Pt** | **Sc** | **ProQ2**  **Global Quality** | **Ramachandran Plots (Molprobity)** | | | **Ramachandran Plots (Procheck)** | | | | **PROSA** | **RMSD** |
| --- | --- | --- | --- | --- | --- | --- | --- | --- | --- | --- | --- |
|  |  |  |  |  |  |  |  |  |  | **Z-Score** |  |
|  |  |  | **Rfr** | **Raar** | **Outliers** | **Rmfr** | **Raar** | **Rgar** | **Rdr** |  |  |
| AChE | Unique | 456.07 | 88.2 | 97.6 | 2.43 | 85.4 | 12.9 | 0.9 | 0.9 | -10.61 | 0.000 |
| JHBP | Unique | 203.81 | 95.9 | 98.9 | 1.12 | 90.6 | 8.2 | 0.8 | 0.4 | -7.78 | 0.180 |
| MET | Unique | 68.89 | 96.0 | 98.0 | 2.02 | 90.1 | 7.7 | 2.2 | 0.0 | -4.61 | 0.102 |

**Pt** = Protein Target; **Sc** = Structure conformation; **Rfr** = Residues in the most favored region; **Raar** = Residues in additional allowed regions; **Outliers** = Ramachandran outliers; **Rmfr** = Residues in the most favored region; **Raar** = Residues in additional allowed regions; **Rgar** = Residues in generously allowed regions; **Rdr** = Residues in disallowed regions; **RMSD** = RMSD align to PBD template.

**Table S6. Quality assessment of protein target model made using the platform Swiss-Model.**

| **Pt** | **Sc** | **GMQE** | **Z-QMEAN** | **Ramachandran Plots (Molprobity)** | | | **Ramachandran Plots (Procheck)** | | | | **PROSA** | **RMSD** |
| --- | --- | --- | --- | --- | --- | --- | --- | --- | --- | --- | --- | --- |
|  |  |  |  |  |  |  |  |  |  |  | **Z-Score** |  |
|  |  |  |  | **Rfr** | **Raar** | **Outliers** | **Rmfr** | **Raar** | **Rgar** | **Rdr** |  |  |
| AChE | Unique | 0.57 | -1.46 | 91.2 | 97.9 | 2.11 | 85.0 | 13.1 | 1.2 | 0.6 | -10.92 | 0.394 |
| EcNR | Agonist | 0.69 | -1.50 | 95.8 | 98.3 | 1.69 | 91.5 | 6.7 | 0.9 | 0.9 | -8.16 | 0.094 |
| JHBP | Unique | 0.89 | -0.99 | 97.8 | 100.0 | 0.00 | 92.4 | 7.4 | 0.0 | 0.4 | -8.12 | 0.210 |
| MET | Unique | 0.67 | -2.85 | 94.9 | 99.0 | 1.01 | 90.1 | 6.6 | 3.3 | 0.0 | -4.65 | 0.099 |
| GABAaR | Agonist | 0.76 | -3.06 | 95.6 | 98.8 | 1.22 | 93.4 | 5.9 | 0.3 | 0.3 | -3.36 | 0.137 |
| GABAaR | Antagonist | 0.76 | -3.06 | 95.6 | 98.8 | 1.22 | 93.4 | 5.9 | 0.3 | 0.3 | -3.36 | 0.137 |
| OctpR-6k42 | Agonist | 0.63 | -4.76 | 92.2 | 98.9 | 1.06 | 88.5 | 10.3 | 0.8 | 0.4 | -3.64 | 0.098 |
| OctpR-7dhi | Agonist | 0.68 | -4.49 | 93.3 | 98.2 | 1.75 | 87.5 | 11.7 | 0.8 | 0.0 | -3.61 | 0.111 |
| OctpR-5d6l | Antagonist | 0.67 | -3.51 | 96.5 | 99.6 | 0.35 | 94.3 | 4.9 | 0.4 | 0.4 | -4.47 | 0.133 |
| OctpR-6prz | Antagonist | 0.66 | -3.61 | 95.8 | 98.9 | 1.06 | 93.9 | 5.0 | 0.0 | 1.1 | -4.18 | 0.118 |

**Pt** = Protein Target; **Sc** = Structure conformation; **Rfr** = Residues in the most favored region; **Raar** = Residues in additional allowed regions; **Outliers** = Ramachandran outliers; **Rmfr** = Residues in the most favored region; **Raar** = Residues in additional allowed regions; **Rgar** = Residues in generously allowed regions; **Rdr** = Residues in disallowed regions; **RMSD** = RMSD align to PBD template.

**Table S7. More common chemical Features of the Tanimoto similarity clusters for the multiple target major hits at the database.**

|  | **Chemical Features of the Clusters and comparisons with canonical plant compounds described in [25]** |
| --- | --- |
| **Cluster A** | Aliphatic sesquiterpene C15, structurally similar to ocimene skeleton. It is a polymer of three isoprene units, forming a JH-like structure. |
| **Cluster B** | is Similar to the phytosteroid with three rings C6, similar to the phenanthrene skeleton. The oxygenated group is reduced to ketone form. |
| **Cluster C1** | Monocyclic and bicyclic sesquiterpenes. |
| **Cluster C2** | Cyclic sesquiterpenes with unique arrangements resembling patchoulene skeletons. Tricyclic, bicyclic, and monocyclic sesquiterpene. |
| **Cluster C3** | Diterpenes polymer of four isoprenes units C20 resembling the basic structure of the phytosteroid, clustered together by having a hydroxylated structure. |
| **Cluster C4** | Polycyclic sesquiterpenes are the same as the first in cluster C3 but without oxygenation. |
| **Cluster C5** | Cluster C5 is the same as the two compounds in C1 but are monocyclic oxygenated sesquiterpenes. |
| **Cluster C6** | Sesquiterpenes with diverse skeleton C15 structures all having three oxygens in the skeleton. |
| **Cluster C7** | Cluster C7 differs from cluster C1 only in the esterification. They are esters of monocyclic sesquiterpenes. It is very reminiscent of the Juvenile hormone. The carbonyl C=O-CH_3_ structures in this cluster are structurally very similar to the juvenile hormone. |
| **Cluster C8** | Sesquiterpenes. |
| **Cluster C9** | Diterpenes. |
| **Cluster C10** | Sesquiterpenes. |
| **Cluster C11** | Sesquiterpenes. |
| **Cluster D** | Diterpenes. |
| **Cluster E** | Cluster E belongs to esters compounds, the monoterpene ester and a sesquiterpene ester. All of them are terpenoid esters with open chains. |
| **Cluster F** | Basic Phytosteroid skeletons, with the first ring presenting 5 and the second with seven elements. |
| **Cluster G** | Terpenoids with a phenyl group linked to a side aliphatic chain presenting an isoprene unit at the extremity. Carbon skeleton topology is relatively like one of the biogenic amines, like octopamine, epinephrine, norepinephrine, adrenaline, dopamine, phenethylamine, and trace amines, among others; even there are no nitrogen groups in their structures and the aliphatic principal chain present one to two more elements in length. Suggest chemical signature related to octopamine receptor agonist ligands. |
| **Cluster H** | Polyoxygenated bicyclic sesquiterpene C15. |
| **Cluster I** | Simple esters, common in pheromones with short chains, many having odors like pineapple, green apple, and grape. It belongs to short-chain pheromones. |
| **Cluster J** | 9-octodecyne. Describe the essential oil components of at least one of the reference papers used to build the compound database. Although there is a probability of being a column bleed artifact from the chromatography technique, it was included in the database due to having been cited in the references and due to the similarity of some parts of its aliphatic hydrocarbon chain with those of some of the more hydrophobic compounds from the dataset (i.e., more similar to fatty acid chains). |

##

##

## **Chromatographic Analysis of the plant species’ Essential oils**

**Tables S8. Chemical composition of the essential oils of *Baccharis dracunculifolia*, *Baccharis retusa* e *Disynaphia spathulata*.**

| **Nº** | **Compound** | **RI** | **Lit.** | **Area (> 1% ±SD)** | | |
| --- | --- | --- | --- | --- | --- | --- |
|  |  |  |  | ***B. dracunculifolia*** | ***B. retusa*** | ***D. spathulata*** |
| **Monoterpene Hydrocarbon** | | | | | | |
| 1 | α-Thujene | 925 | 924 | nd | 1.99 | nd |
| 2 | α-Pinene | 932 | 932 | 2.12 | 13.86 | 3.45 |
| 3 | Sabinene | 974 | 969 | 0.12 | nd | nd |
| 4 | β-Pinene | 975 | 974 | 2.66 | 11.78 | nd |
| 5 | β-Myrcene | 990 | 988 | 1.17 | 3.56 | 05.05 |
| 6 | Limonene | 1027 | 1024 | 8.03 | nd | 3.08 |
| 7 | (E)- β-Ocimene | 1045 | 1044 | nd | nd | 4.24 |
| **Sesquiterpene Hydrocarbon** | | | | | | |
| 8 | (E)-Caryophyllene | 1417 | 1417 | 7.68 | 28.87 | 3.84 |
| 9 | α-Humulene | 1450 | 1452 | nd | nd | 2.16 |
| 10 | Germacrene-D | 1480 | 1480 | 9.53 | 17.02 | 38.26 |
| 11 | Bicyclogermacrene | 1496 | 1500 | 12.03 | nd | 23.69 |
| 12 | δ-Cadinene | 1522 | 1522 | 4.18 | 2.81 | 02.03 |
| **Sesquiterpene oxygenated** | | | | | | |
| 13 | (E)-Nerolidol | 1564 | 1561 | 25.86 | nd | nd |
| 14 | Spathulenol | 1574 | 1577 | 10.19 | nd | nd |
| 15 | Caryophyllene oxide | 1580 | 1582 | 03.06 | 1.96 | nd |

*Retention index relative to the n-alkane series (C8–C20) in the HP-5 MS column in order of elution. Area (>1%): mean of the relative area of the chromatographic peaks above 1%. SD, standard deviation (n=3). nd, not detected or area percent below 1%.

##

## **Ligand binding site**

Acetylcholinesterase Enzyme (AChE)

**Figure S2. Amino acid sequence (Uniprot ID: D5FQ11) alignment of the Calliphoridae fly *Cochliomyia macellaria* and the protein template *Drosophila melanogaster* fly (PDB ID: 1DX4).**


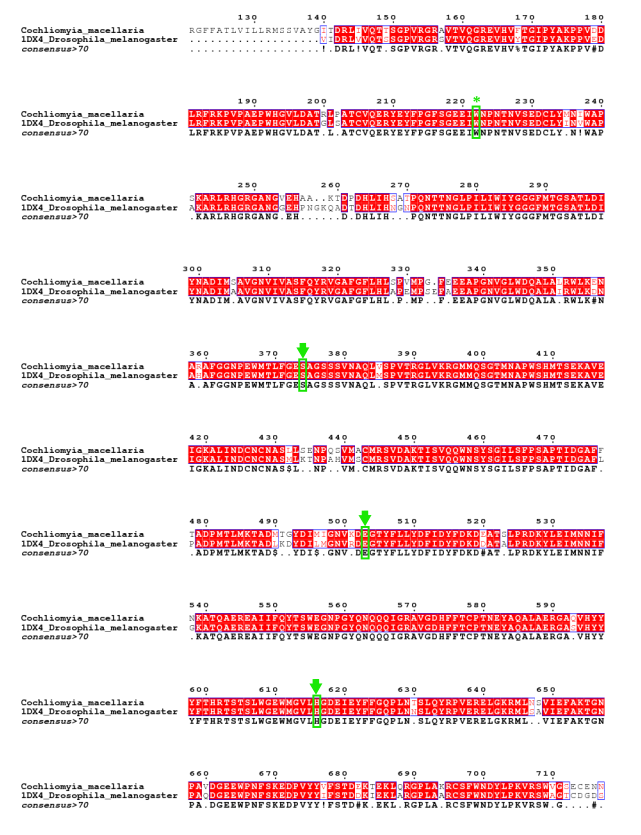


In green, amino acid residues are responsible for the catalytic site. Arrows indicate the members of the catalytic triad, and a circle indicates the main component of the choline-binding site, according to da Silva et al., 2011 [**1**].

**Figure S3. The binding site of Ligand showing, in red, the AChE model by Homology and in green the PDB Template 1DX4.**


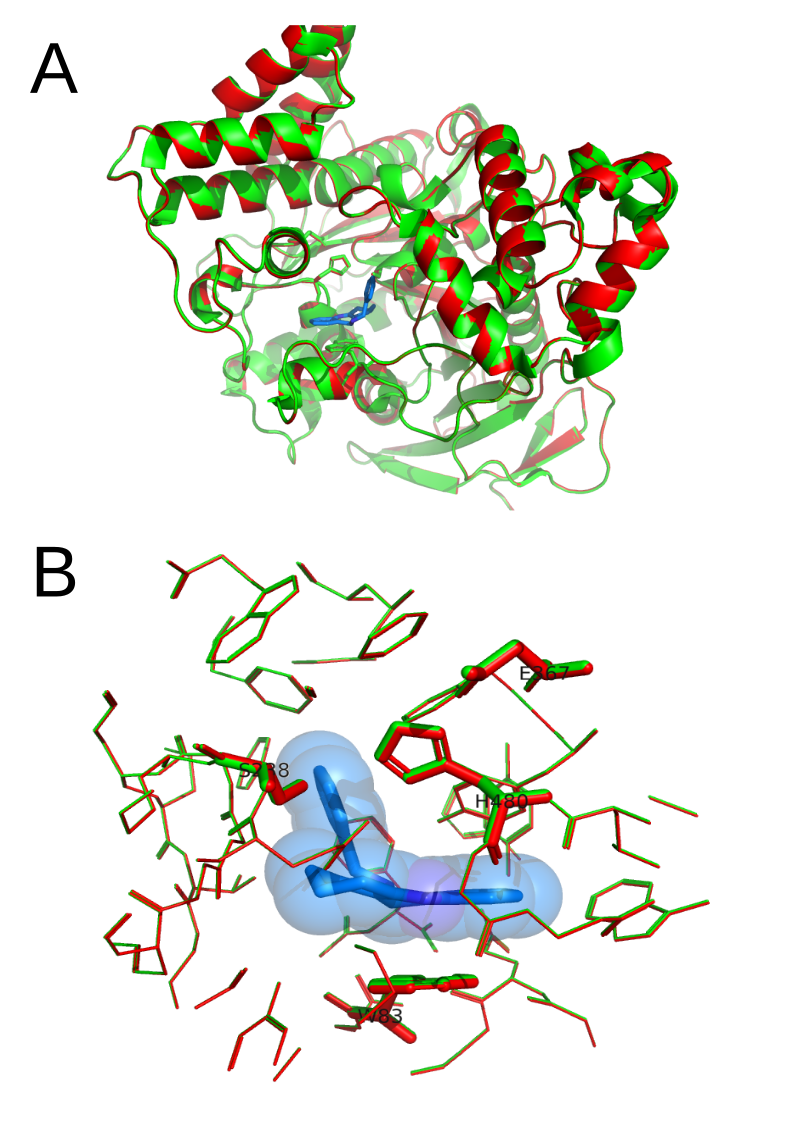


In sticks form showing the catalytic triad with the main component of the choline-binding site. Amino acids presented in line form are the residues 8 Angstrom far from the center of the crystallographic ligand, 9-N-Phenylmethylamino-Tacrine.

Ecdysone Receptor (EcNR)

**Figure S4. Amino acid sequence (Uniprot ID: A0A0L0BTL1) alignment of the**  **Calliphoridae fly *Lucilia cuprina* and the protein template of sheep lice *Bovicola ovis* (PDB ID: 4OZT).**


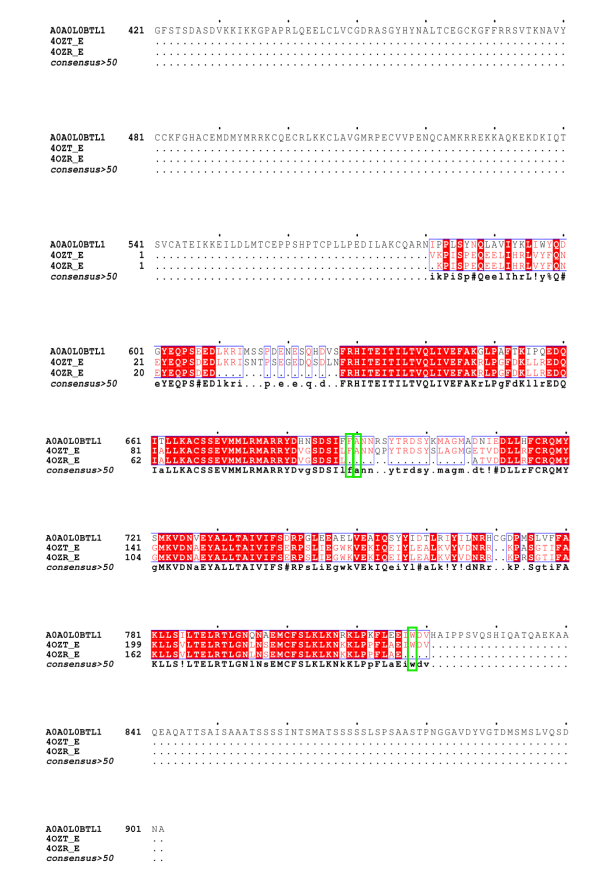


In green amino acids, residues are responsible for sealing the ligand-binding pocket at one end of the Trp516, acting as a lid and forming hydrophobic interaction with the alkyl tail of crystallographic ligand Ponasterone A (PonA). A β-sheet seals the pocket's opposite end, forming a hydrophobic interaction involving Phe3889 and a hydrogen bond between Ala390 and PonA [**4**].

**Figure S5. The binding site of Ligand shows the EcNR model by Homology in red and green the PDB Template 4OZT.**

**
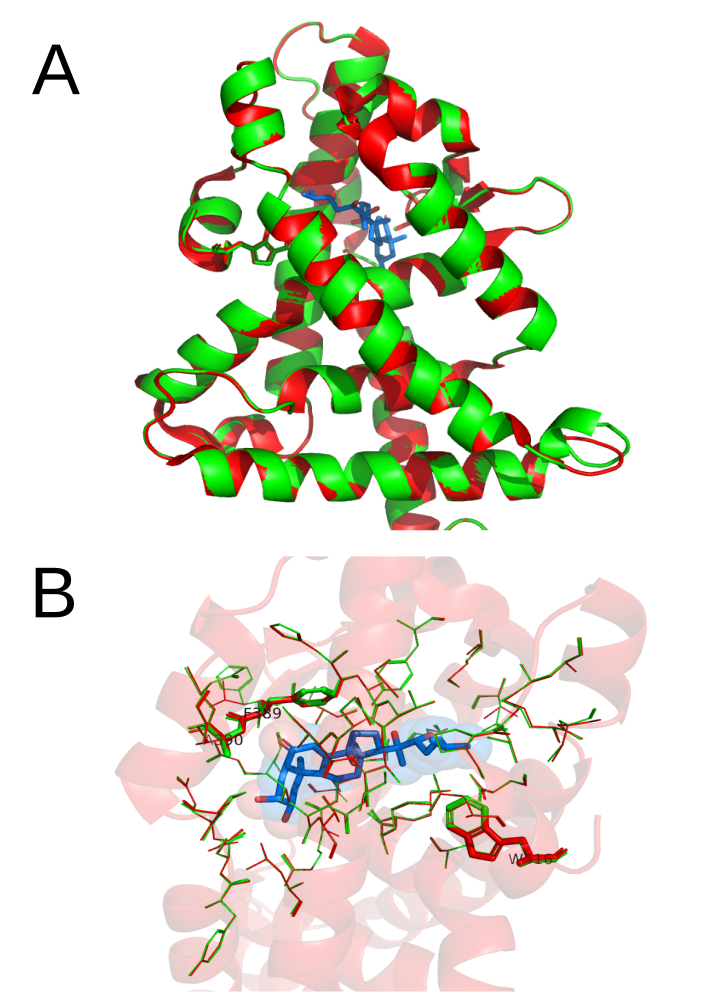
**

In stick form showing amino acids residues responsible for sealing the ligand-binding pocket at one end, the W516 acts as a lid and forms hydrophobic interaction with the alkyl tail of crystallographic ligand Ponasterone A (PonA). A β-sheet seals the pocket's opposite end, forming a hydrophobic interaction involving F389 and a hydrogen bond between A390 and PonA, according to Ren et al. (2014) [**4**]. Amino acids presented in line form are the residues 8 Angstrom far from the center of the crystallographic ligand, Ponasterone A (PonA).

Juvenile Hormone Binding Protein (JHBP)

**Figure S6. Amino acid sequence model alignment with protein template *Aedes aegypti* (PDB ID: 5V13).**


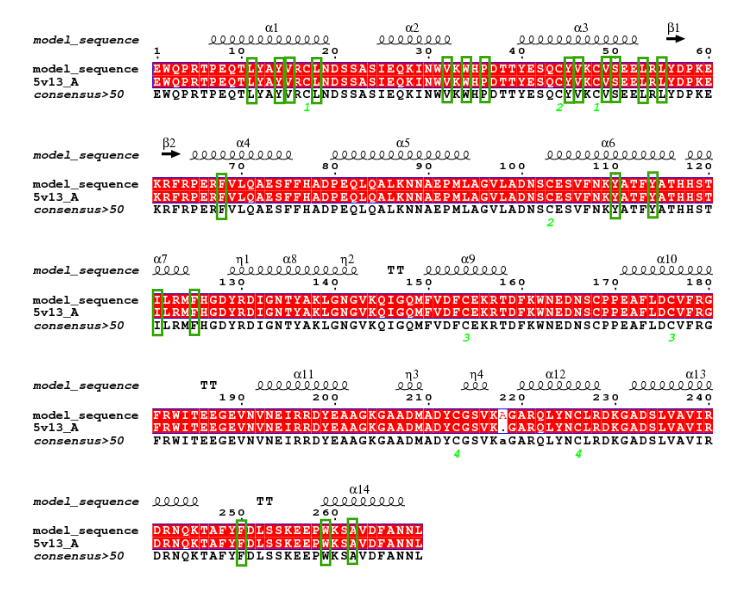


It is highlighted in the green residues indicate conserved binding pocket sites that contact the bound ligand in the *Ae. aegypti* mJHBP–JH III complex, according to Kim et al. (2017) [**5**].

* It is important to remark that, only for this protein target, the *Aedes aegypti* template (5V13) sequence is used as the primary sequence to be modeled. This is done because this amino acid sequence is the founded dipteran JHBP protein sequence closer to the Calliphoridae family. And seeking to adopt the same protocol for all protein targets, the homology-modeling procedures are identically adopted for the juvenile hormone binding protein (JHBP) template sequence.

**Figure S7. Ligand binding site Model (in red) and protein template (in green) of *Drosophila melanogaster* fly (PDB ID: 5V13).**


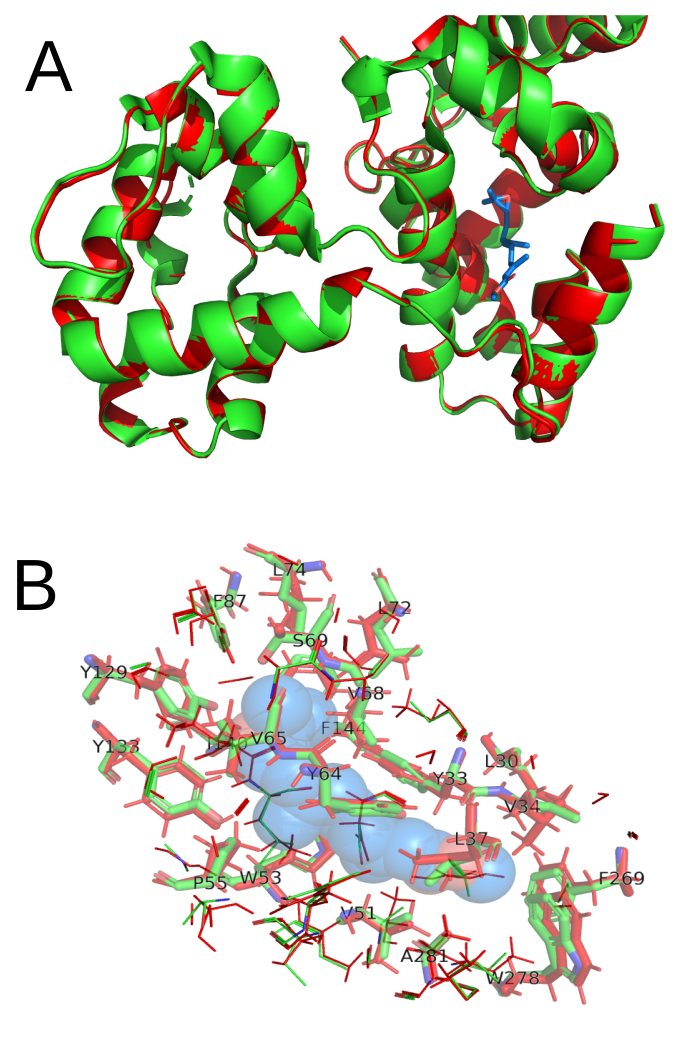


In the sticks shows, the residues indicate conserved binding pocket sites that contact the ligand in the *A. aegypti* mJHBP–JH III complex, according to Kim et al. (2017) [**5**]. Residues shown in line represent the 8 Angstrom far from crystallographic ligand JH III.

Methoprene-Tolerant Receptor (MET)

**Figure S8. The amino acid sequence of *Lucilia cuprina* (Uniprot ID: A0A0L0C1U9_LUCCU) aligned with the protein template of Homo sapiens (PDB ID: 3F1P).**


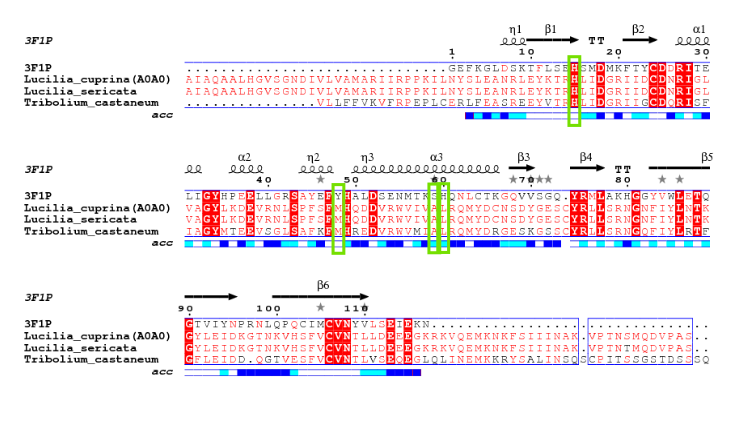


It is highlighted in green to indicate residues that interact with the ligand, as mentioned in Scheuermann et al. (2009) [**6**].

**Figure S9. Ligand binding site Model, in red, and protein template (PDB ID: 3F1P) PAS Domain of *Homo sapiens*, in green**


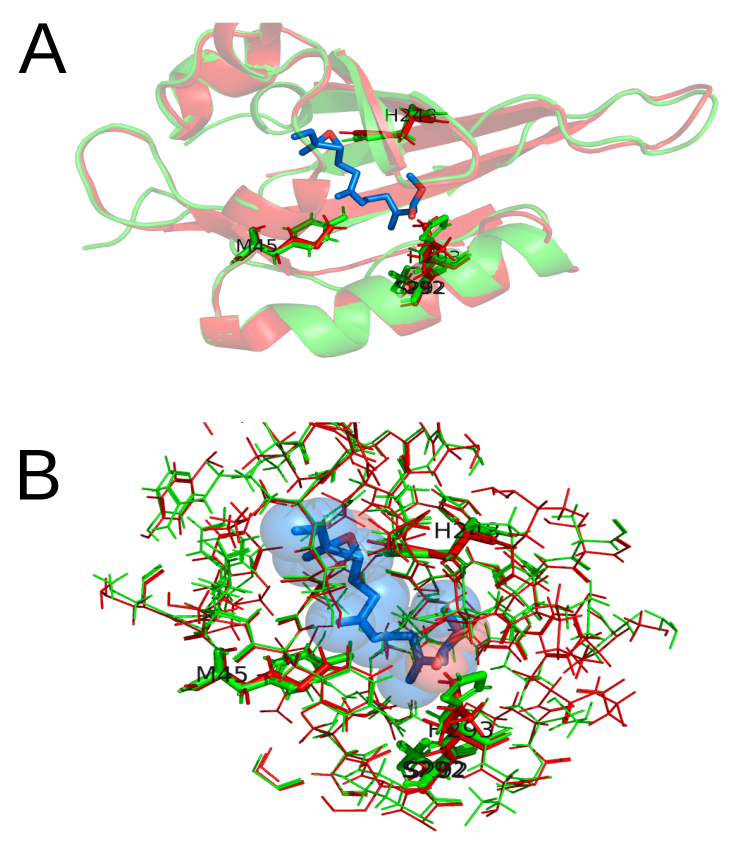


Residues in sticks form and labeled indicate that they interact with the ligand according to mentioned in Scheuermann et al. (2009) [**6**]. Residues presented in line format represent all residues 8 Angstrom far from the “ligand interacting residues.” The blue ligand is the true natural ligand Juvenile Hormone III docked in protein model.

Gamma-Aminobutyric Acid Receptor (GABAaR)

**Figure S10. Amino acid sequence (Uniprot ID: O17145) alignment of the Calliphoridae fly *Lucilia cuprina* and the protein template *Homo sapiens* (PDB ID: 4COF).**


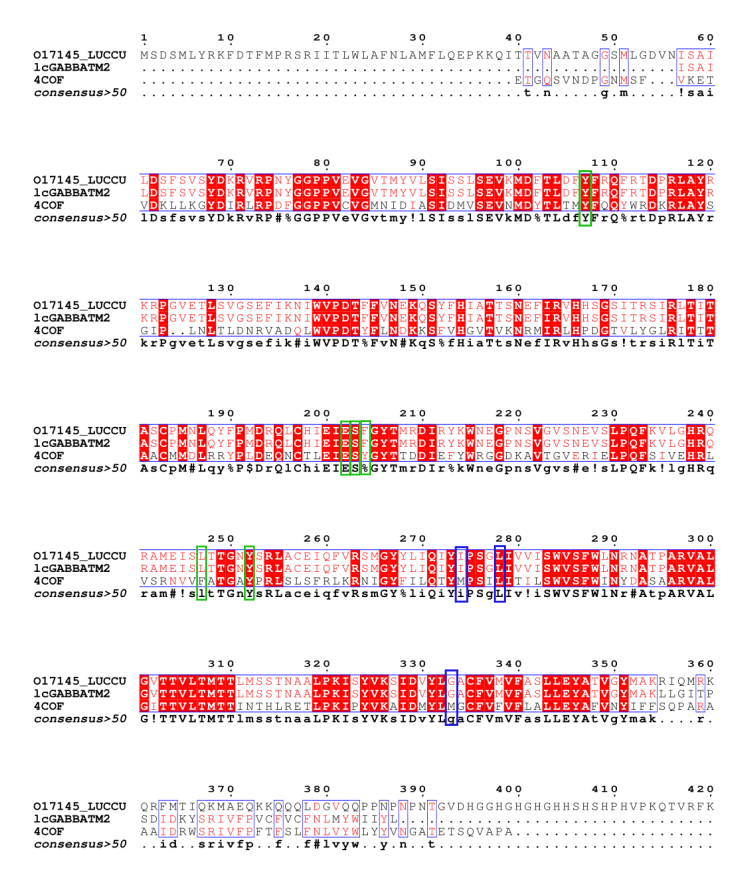


Highlighted in green amino acids residues involved in agonist binding pocket according to Miller and Aricescu (2014) [**7**]. And highlighted in blue are shown residues involved in the antagonist binding pocket according to Gao et al., 2020 [**26**]

**Figure S11. The ligand binding site in the Model is shown in red, and the protein template *Homo sapiens’* γ-aminobutyric acid receptors (GABAaR) are shown in green. Agonist and Antagonist binding sites are presented at the same protein structure bonded to crystallographic ligand Benzamidine and docked antagonist RDL GABA Meta-diamide, respectively.**


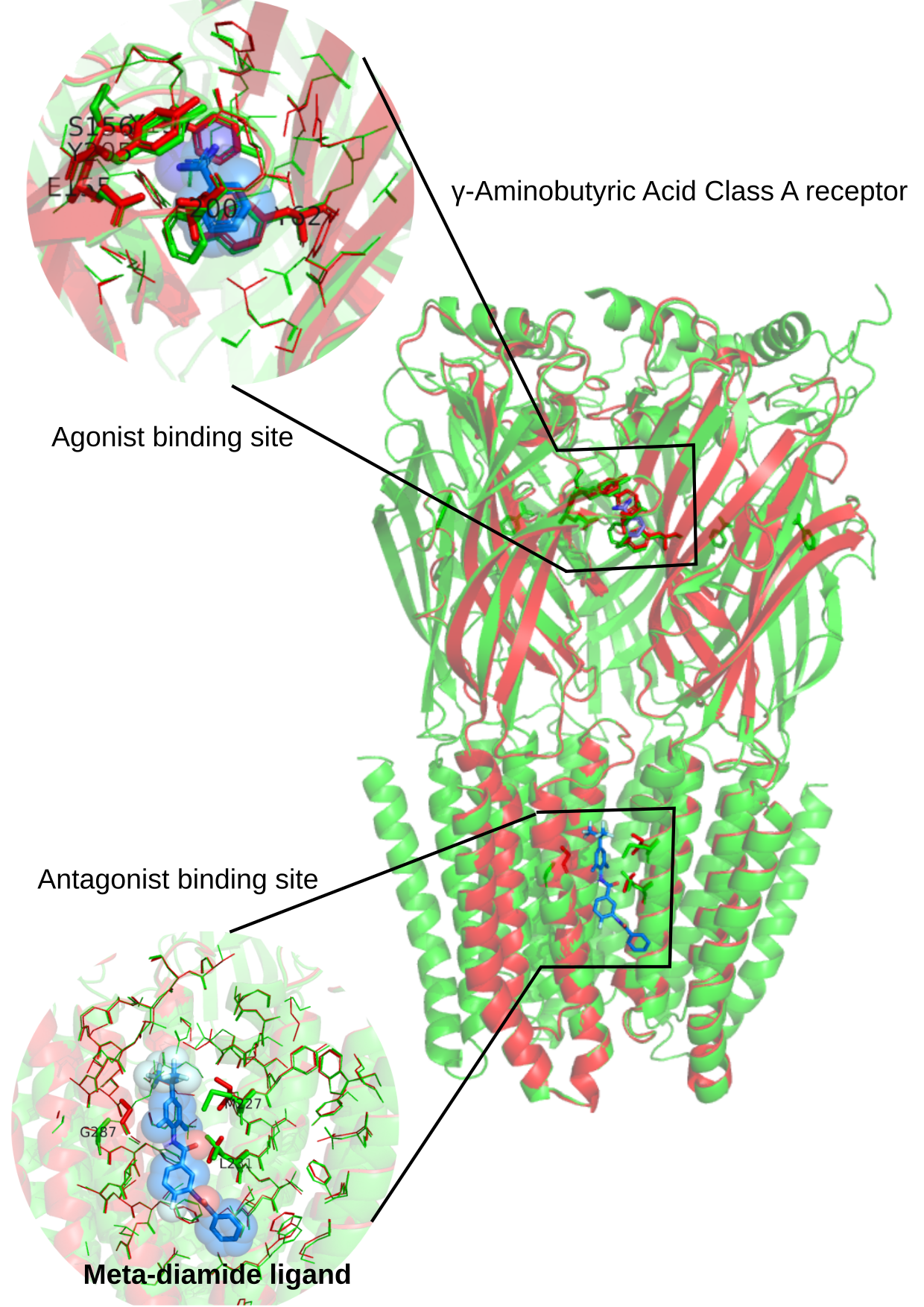


Residues involved in the agonist binding pocket are presented, according to Miller and Aricescu (2014) [**7**]. Residues involved in the antagonist binding pocket are shown, as mentioned in Gao et al., 2020 [**8**].

Octopamine receptor (OctpR)

**Figure S12. Model amino acid sequence alignment template amino acid sequence of receptor-Gs protein templates in an agonist conformation (PDB ID: 7DHI and 6K52) and antagonist conformation (PDB ID: 5D6L and 6PRZ).**


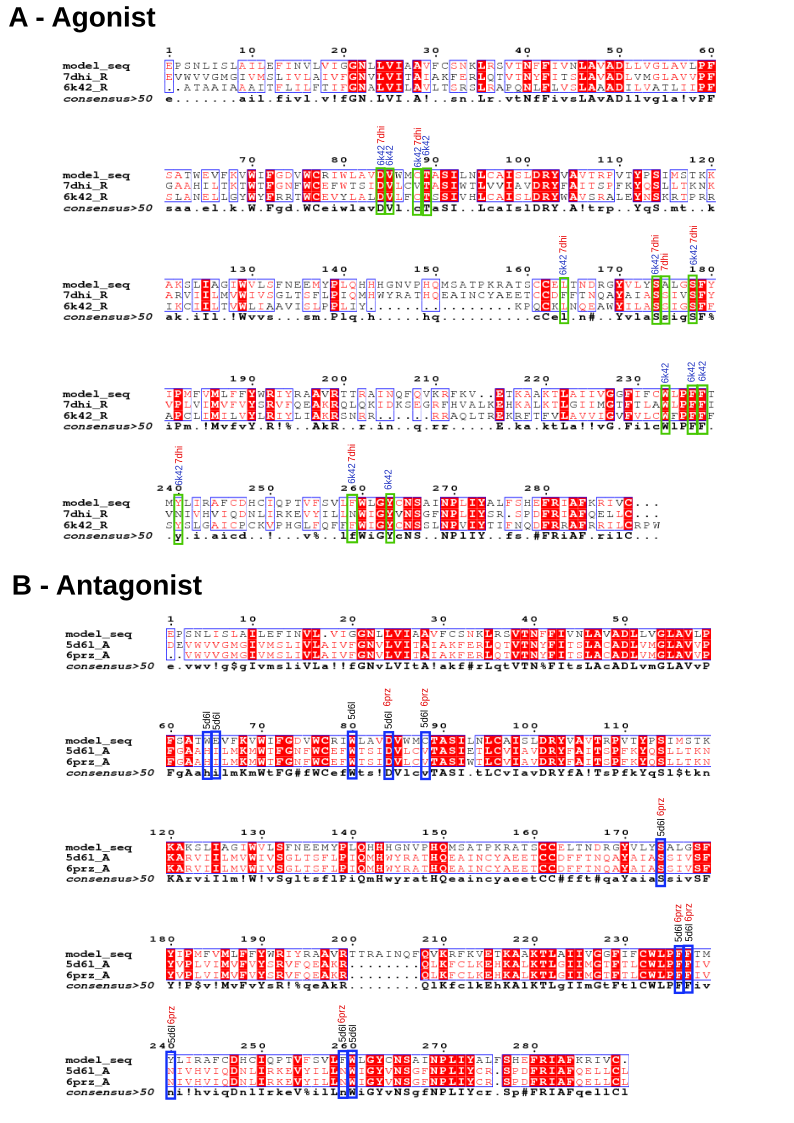


In A (Agonist conformation), protein residues highlighted in green are amino acid residues involved in the agonist binding pocket. For Template 6K42, according to Yuan et al., 2020 [**8**], and for Template 7DHI, according to Yang et al., 2020 [**9**]. And in B (Antagonist conformation), residues highlighted in blue are shown that are involved in the antagonist binding pocket, according to Ishchenko et al., 2020 [**12**] for both templates 5D6L and 6PRZ. The model sequence is equal for both conformations.

**Figure S13. The ligand binding site in the protein homology model is shown in red, and the protein template for the Octopamine receptor (OctpR) is shown in green. Antagonist and Agonist binding sites are presented in the figure details.**


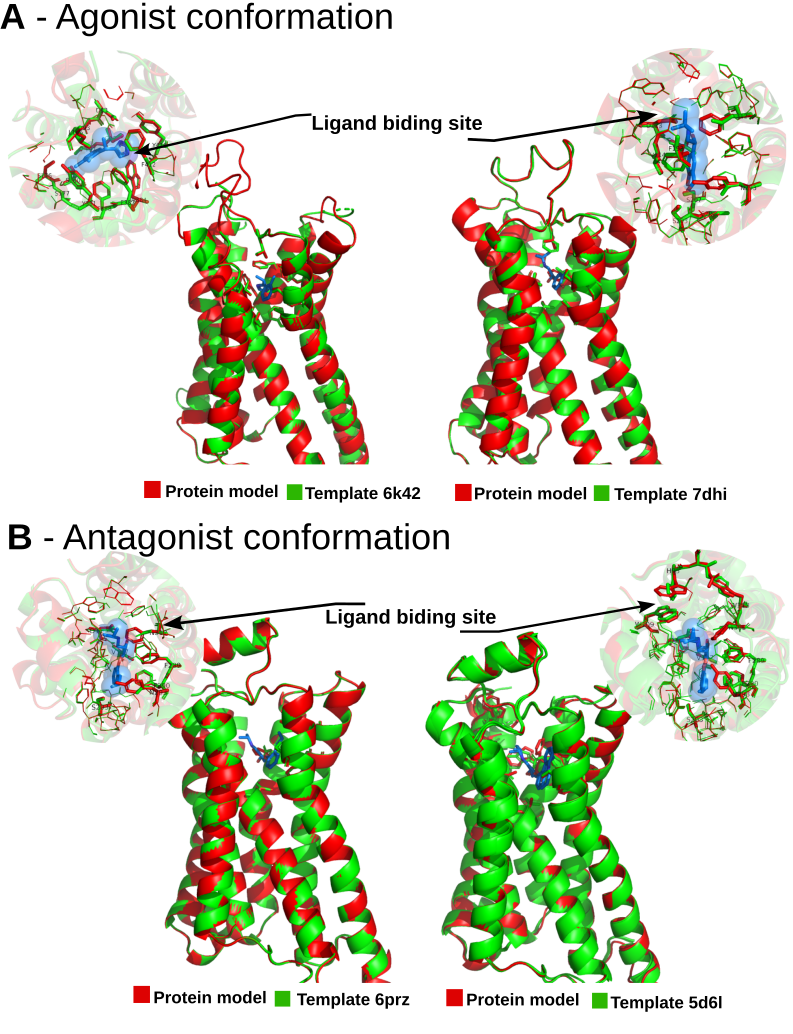


**Docking Grid Box**

A protocol for determining the correct Grid Box to dock in Vina as used for all target protein models.

First, the critical residues as capacity sites or binding sites for receptors are established based on the literature on the Template PDB, references of the Calliphoridae fly obtained on the page of the amino acid sequence of Uniprot and in the literature about the target biochemistry mechanism. Crystallographic ligands were determined in the PDB template.

Templates and models have aligned on PyMOL using the command: “align model. template and name c+ca+n+o”. The residues 8 Angstrom far from the ligand or the critical residues are determined on PyMOL by selecting the ligand or the critical residues and using the PyMOL command: “select sele around 8” to select the 8 Angstrom far amino acid residues, and after that, the command “show sticks. sele”. These core amino acid residues must be inside the grid box.

The PyMOL Plugin Autodock/Vina was used to center and size the grid box determined by increasing the x, y and z dimensions until all the amino acid residues were 8 Angstrom far from the critical residues or ligands and became inside the grid box.

A config.txt file was annotated with the Grid-Box configuration and used in docking experiments. The Grid-box dimensions in Å for each blowfly protein target obtained are presented in Table S7:

**Table S9. Grid-Box dimension used on virtual docking experiments.**

| **Target** | **Center (x. y. z)** | **Dimensions (x. y. z)** |
| --- | --- | --- |
| AChE | -11.43. -43.74. 30.37 | 25. 20. 20 |
| EcNR Agonist | -10.86. 11.4. 12.96 | 28. 23. 24 |
| JHBP | 239.78. -26.46. 352.50 | 21. 21. 17 |
| MET | 12.64. -40.26. 14.71 | 18. 10. 09 |
| GABBAa Agonist | -1.09. 24.72. 121.28 | 19. 14. 16 |
| GABBAa Antagonist | 15.50. 17.22. 164.12 | 21. 15. 20 |
| OctpR Agonist | 18.96. 4.78. -9.10 | 17. 18. 21 |
| OctpR Antagonist | 18.96. 4.78. -9.10 | 17. 18. 21 |

**Table S10. True Positive ligands (Controls) are used to create the ROC curve and Enrichment Curve.**

| **AChE** | **EcNR** | **JHBP** | **MET** | **GABAaR**  **Agonist** | **GABAaR**  **Antagonist** | **OctpR**  **Agonist** | **OctpR**  **Antagonist** |
| --- | --- | --- | --- | --- | --- | --- | --- |
| Bis Tacrine [**27**] | 20Hydroxyecdysone [**31**. **32**] | 6.7-Dihydro  Juvenile Hormone III [**33**] | Desmethyl Pyriproxyfen [**35**] | Progabide [**36**] | Meta-Diamide 5 [**38**] | Phentolamine [**40**] | Spiperone [**42**] |
| Donepezil [**27**. **28**] | Chromafenozide [**31**] | Ethyl-Ester  Juvenile Hormone [**33**] | Juvenile Hormone 0 [**35**] | Propofol [**36**] | Meta-Diamide 1 [**38**] | Naphazoline [**40**. **41**] | Propranolol [**43**] |
| Galantamine [**27**. **28**] | Cyasterone [**31**] | Juvenile Hormone II [**33**] | Juvenile Hormone I [**35**] | Piperidine-4-Sulfonic-Acid [**36**] | Meta-Diamide 9 [**38**] | Levomedetomidine [**41**] | Promethazine [**41**. **43**] |
| Heptyl Physostigmine [**27**] | Ecdysone [**31**. **32**] | R-Juvenile Hormone III [**34**] | Juvenile Hormone II [**35**] | Gaboxadol (THIP) [**37**] | 3-benzamido-N-phenylbenzamides (BPBs) 9 [**39**] | Dexmedetomidine [**41**] | cis-(Z)-Flupentixol [**43**] |
| Physostigmine [**28**] | Halofenozide [**31**] | S-Juvenile Hormone III [**34**] | Juvenile Hormone III [**35**] | Isoguvacine [**37**] | Meta-Diamide7 [**38**] | Tizanidine [**41**] | Epinastine [**42**] |
| Tacrine [**27**] | Makisterone A [**31**] | Juvenile Hormone III [**34**] | Methoprene [**35**] | 5Amino Valeric Acid [**37**] | Meta-Diamide6 [**38**] | Clonidine [**40**] | Mianserin [**42**] |
| Tolserine [**29**] | Methoxyfenozide [**31**] | Methyl Farnesoate [**33**] | Methyl Farnesoate [**35**] | Muscimol [**37**] | 3-benzamido-N-phenylbenzamides (BPBs) 10 [**39**] | Demethyl Chlordimeform [**42**] |  |
| Velnacrine [**27**] | Ponasterone A [**31,** **32**] |  | Phenoxycarb [**35**] | Amino Butenoic Acid (TACA) [**37**] | Meta-Diamide 8 [**38**] | Tolazoline [**40**] |  |
| 2-Iodo 2-Tetrahydroacridin [**30**] | Rh 5849 [**31**] |  | Pyriproxyfen [**35**] | γ-Aminobutyric Acid (GABA) [**37**] | 3-benzamido-N-phenylbenzamides (BPBs) 8 [**39**] | Synephrine [**40**] |  |
| 2-Iodo 3-Tetrahydroacridin [**30**] | Tebufenozide [**31**] |  | Thiazolyl Analog [**35**] | Taurine [**37**] |  | Octopamine [**40**. **41**] |  |
| 2-Iodo 4-Tetrahydroacridin [**30**] |  |  |  | B-Alanine [**37**] |  |  |  |
| 3-Iodo 2-Tetrahydroacridin [**30**] |  |  |  |  |  |  |  |
| 3-Iodo 4-Tetrahydroacridin [**30**] |  |  |  |  |  |  |  |
| 4-Iodo 2-Tetrahydroacridin [**30**] |  |  |  |  |  |  |  |
| 4-Iodo 3-Tetrahydroacridin [**30**] |  |  |  |  |  |  |  |
| 4-Iodo 4-Tetrahydroacridin [**30**] |  |  |  |  |  |  |  |

**Figure S14. Chemical structures of ligands scored high in virtual screening with blowfly protein targets divided by the hierarchical clusters.**


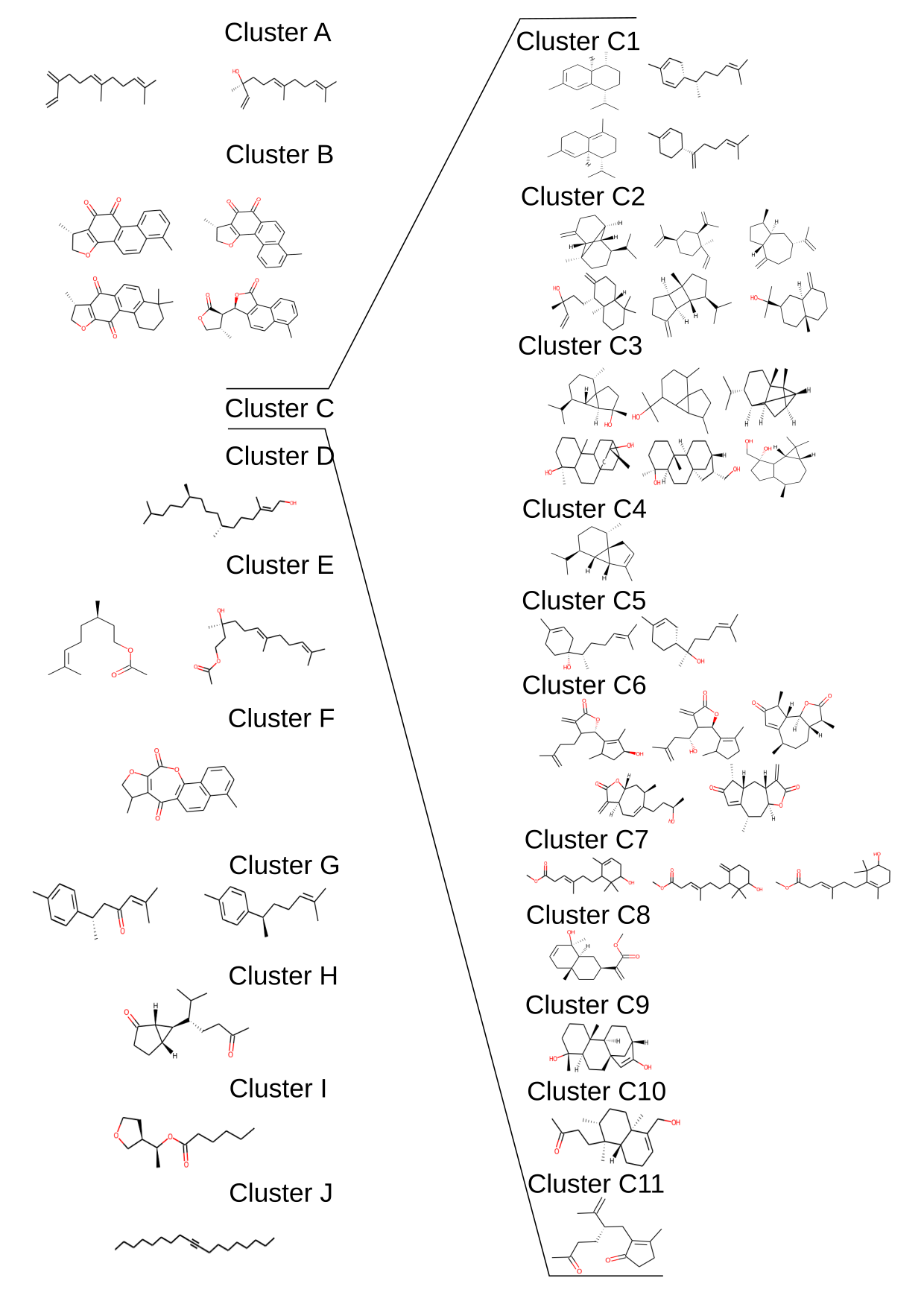


## **ROC (Receiver Operating Characteristics) curve and Enrichment Curve Analysis**

**Figure S15. ROC and Enrichment curves considering true positive ligands and decoys ligands for the protein target Acetylcholinesterase enzyme (AChE). (A) Curve produced using only the highest affinity score for different affinity score functions. and (B) Curve produced using the three high-affinity scores for different affinity score functions. The area under the ROC curve is denoted in green by the initials AUC, and the selected scoring functions for the screening analyses are highlighted in yellow.**

(A)


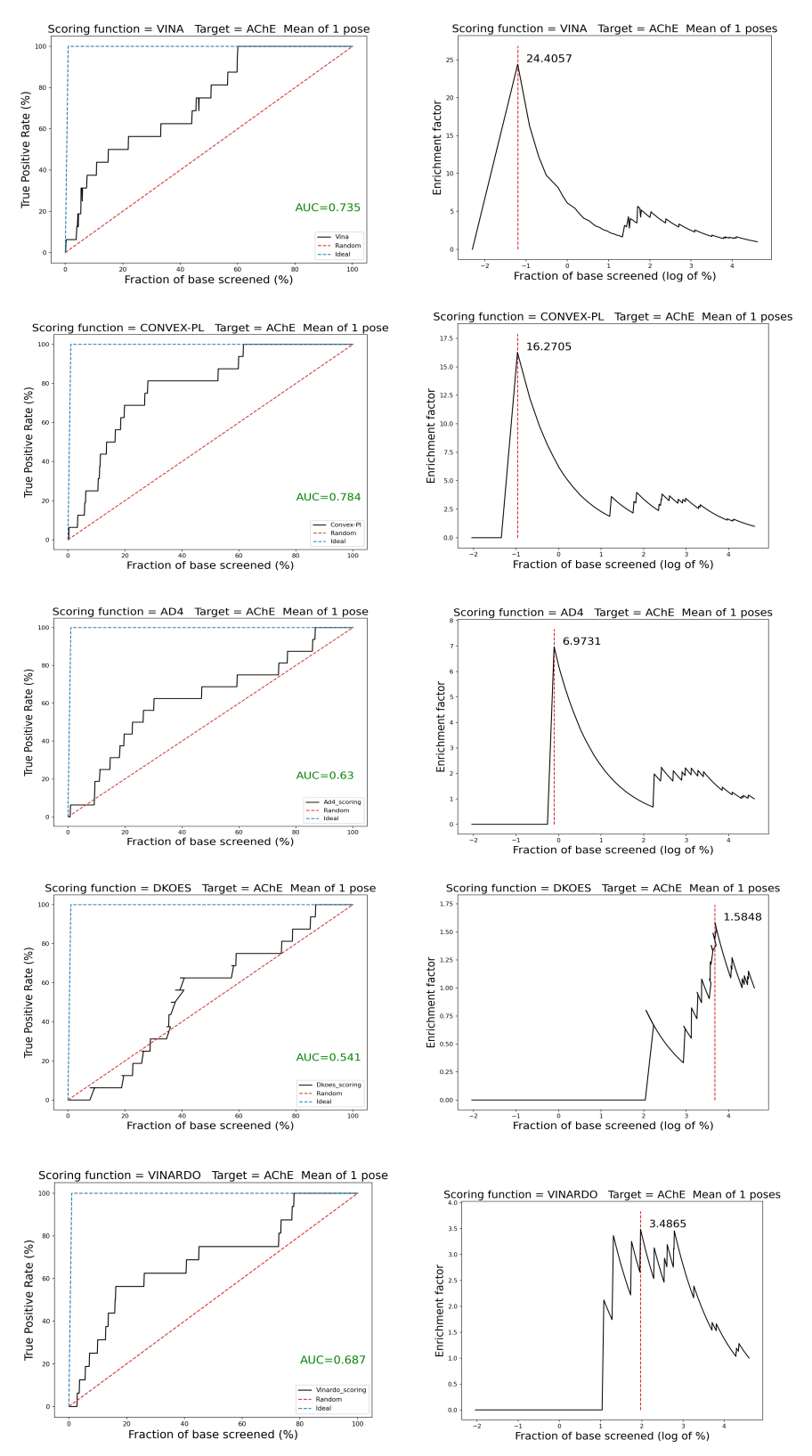


(B)


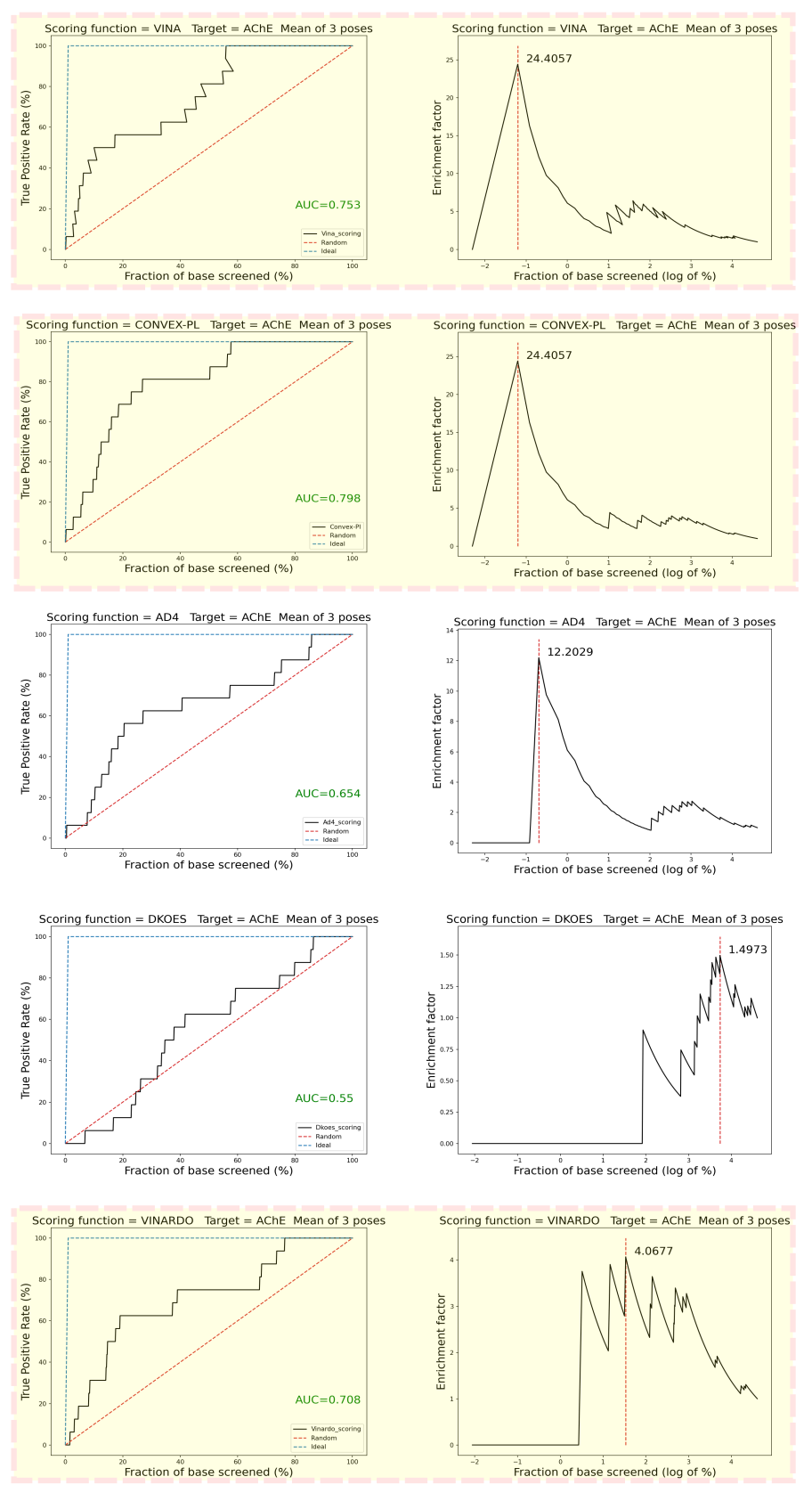


*Score function selected to perform forward virtual screening analysis is highlighted in yellow.*

**Figure S16. ROC and Enrichment curves consider true positive ligands and decoys ligands for the protein target Ecdysone receptor (EcNR). (A) Curve produced using only the highest affinity score for different affinity score functions. and (B) Curve produced using the three high-affinity scores for different affinity score functions. The area under the ROC curve is denoted in green by the initials AUC and the selected scoring functions for the screening analyses are highlighted in yellow.**

(A)


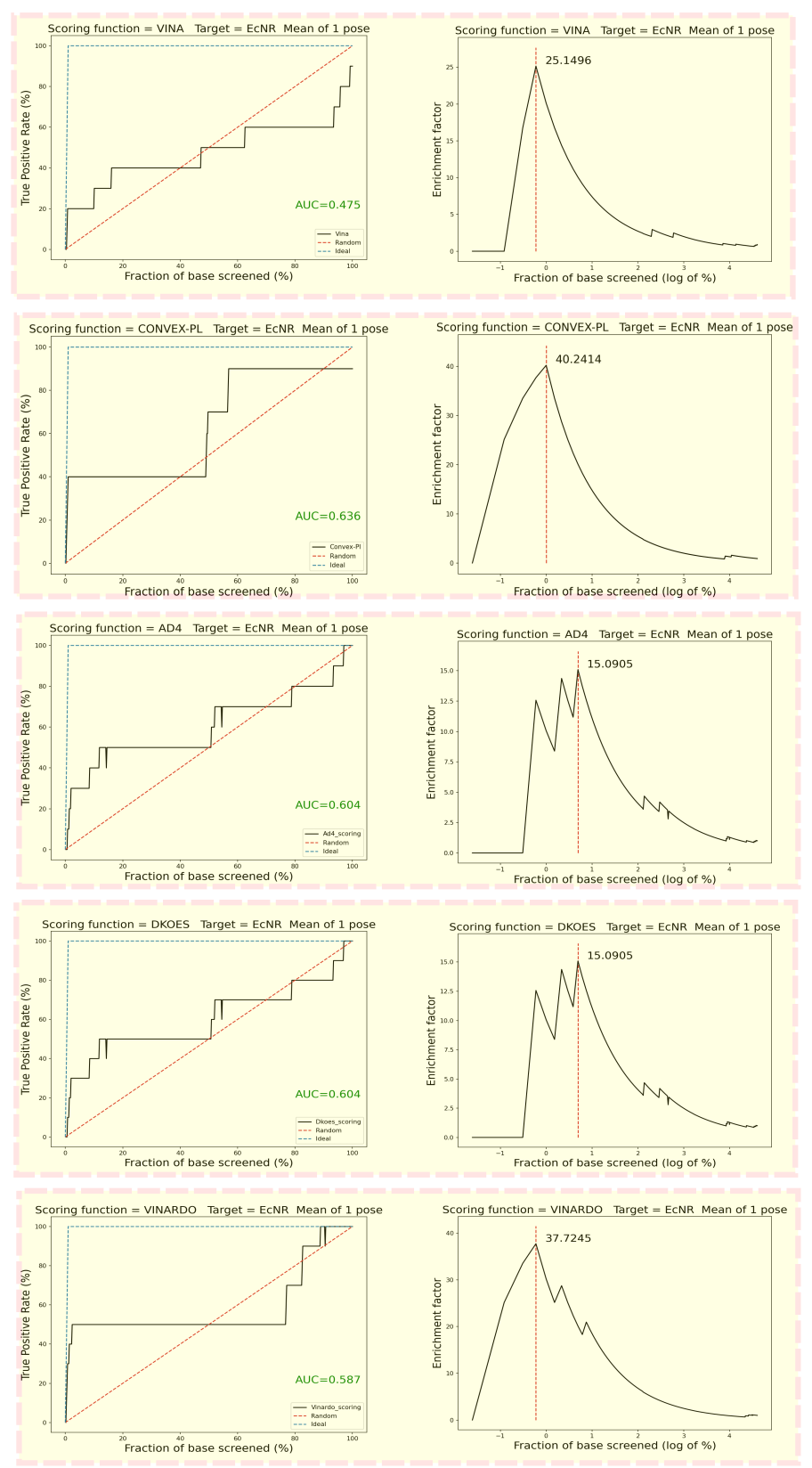


*Score function selected to perform forward virtual screening analysis is highlighted in yellow.*

(B)


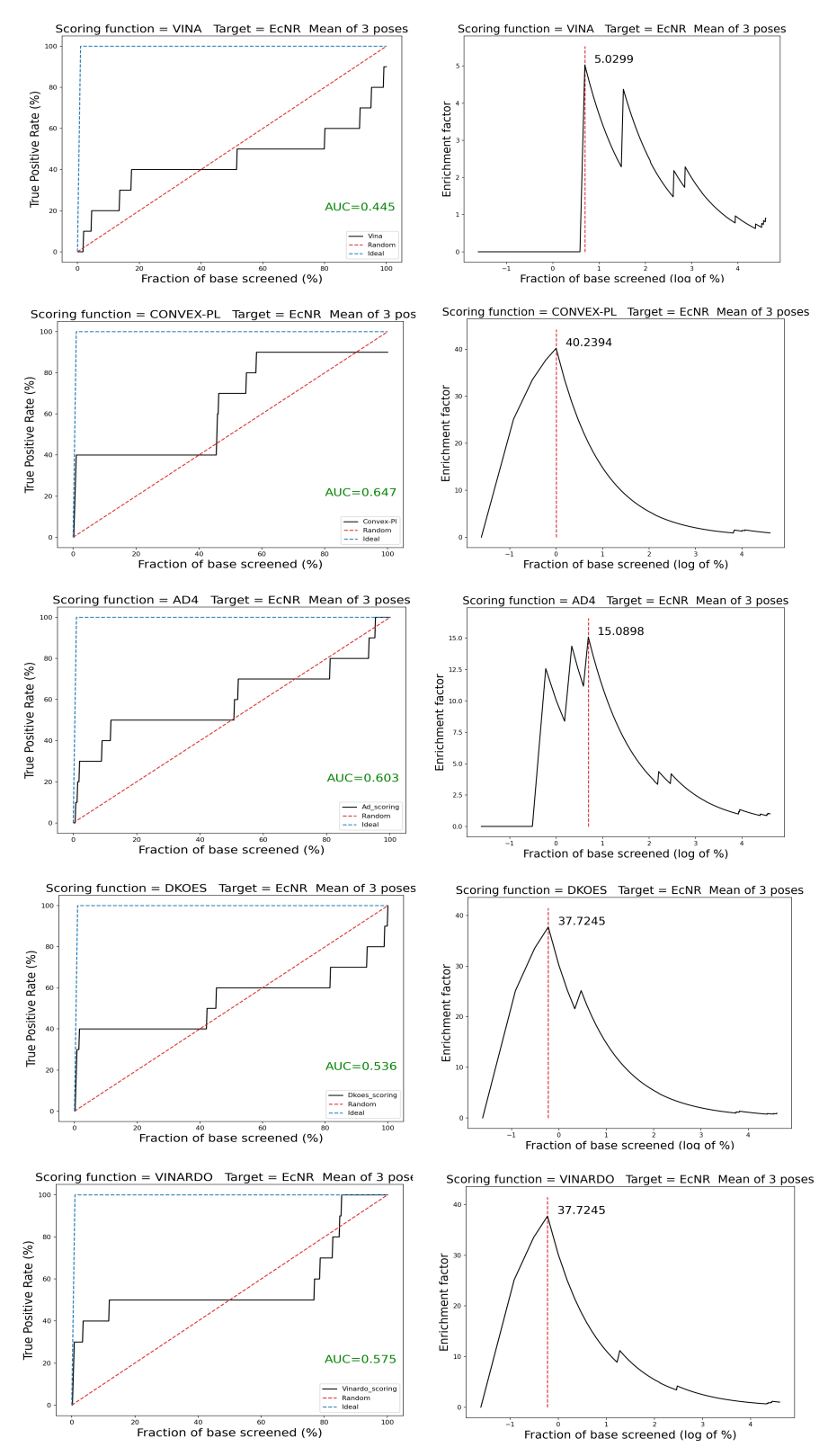
c

**Figure S17. ROC and Enrichment curves considering true positive ligands and decoys ligands for the protein target Juvenile Hormone Binding Protein (JHBP). (A) Curve produced using only the highest affinity score for different affinity score functions. and (B) Curve produced using the three high-affinity scores for different affinity score functions. The area under the ROC curve is denoted in green by the initials AUC and the selected scoring functions for the screening analyses are highlighted in yellow.**

(A)


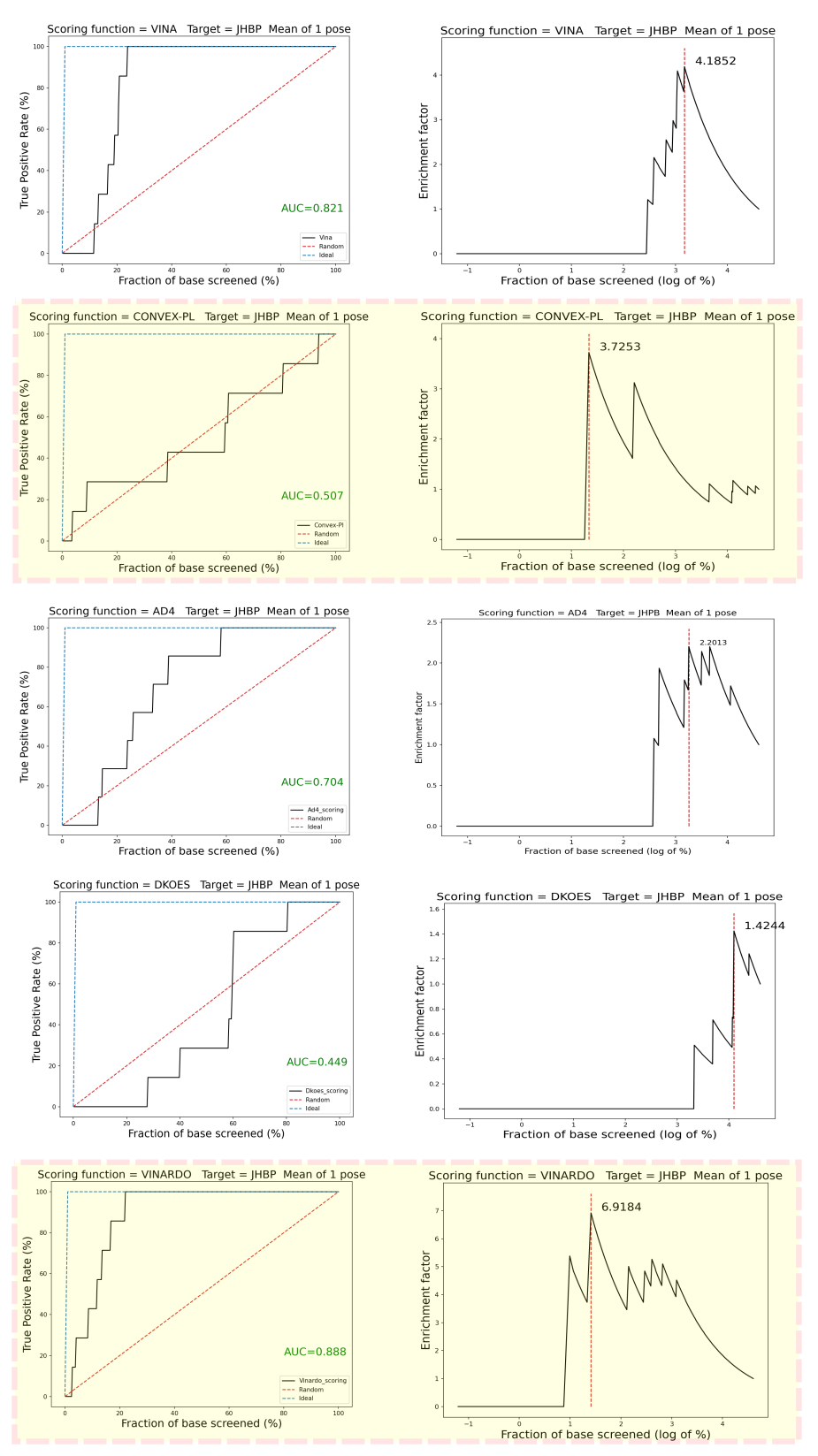


*Score function selected to perform forward virtual screening analysis is highlighted in yellow.*

(B)


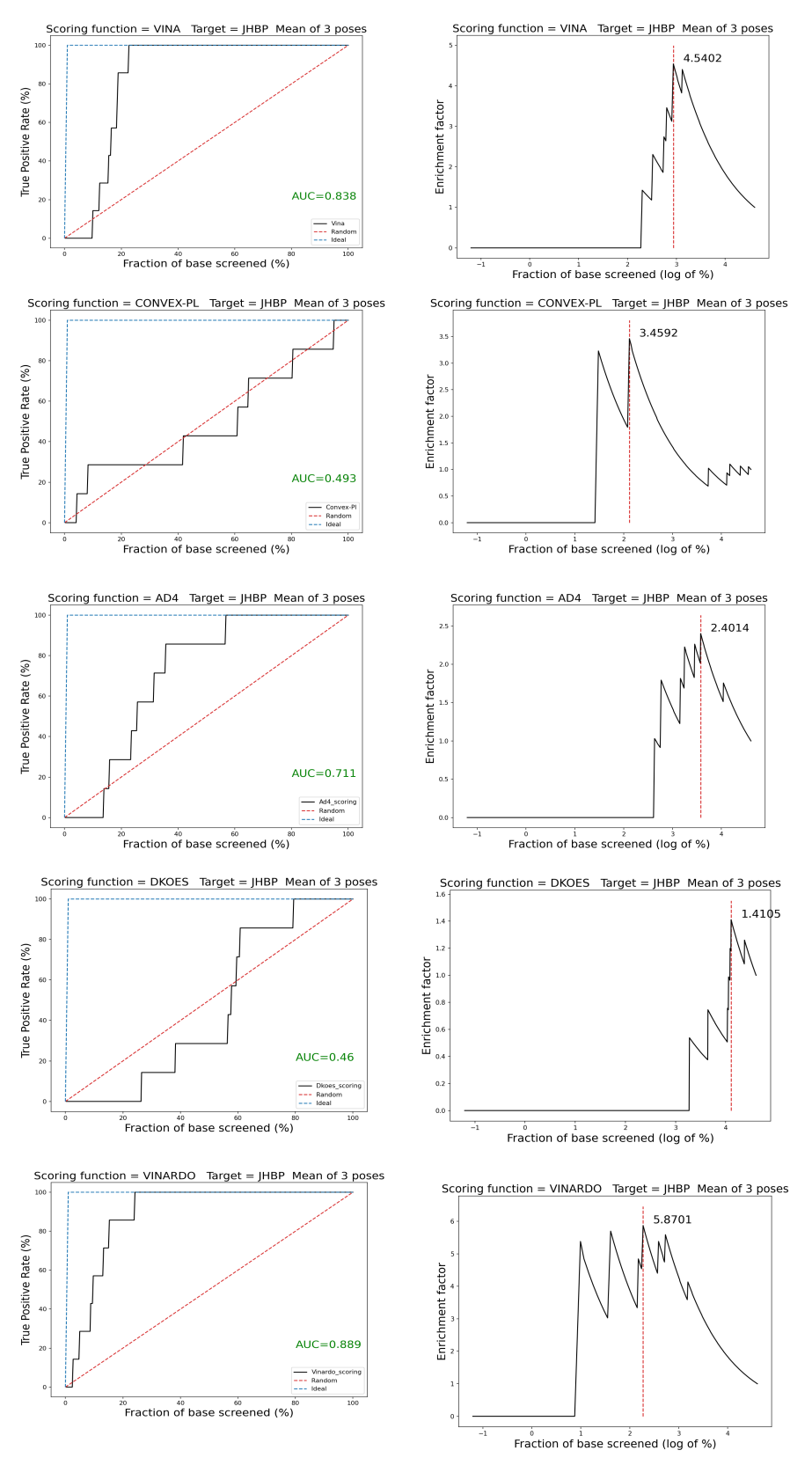


**Figure S18. ROC and Enrichment curves considering true positive ligands and decoys ligands for the protein target PAS domain of Methoprene-Tolerant Receptor (MET). (A) Curve produced using only the highest affinity score for different affinity score functions. and (B) Curve produced using the three high-affinity scores for different affinity score functions. The area under the ROC curve is denoted in green by the initials AUC and the selected scoring functions for the screening analyses are highlighted in yellow.**

(A)
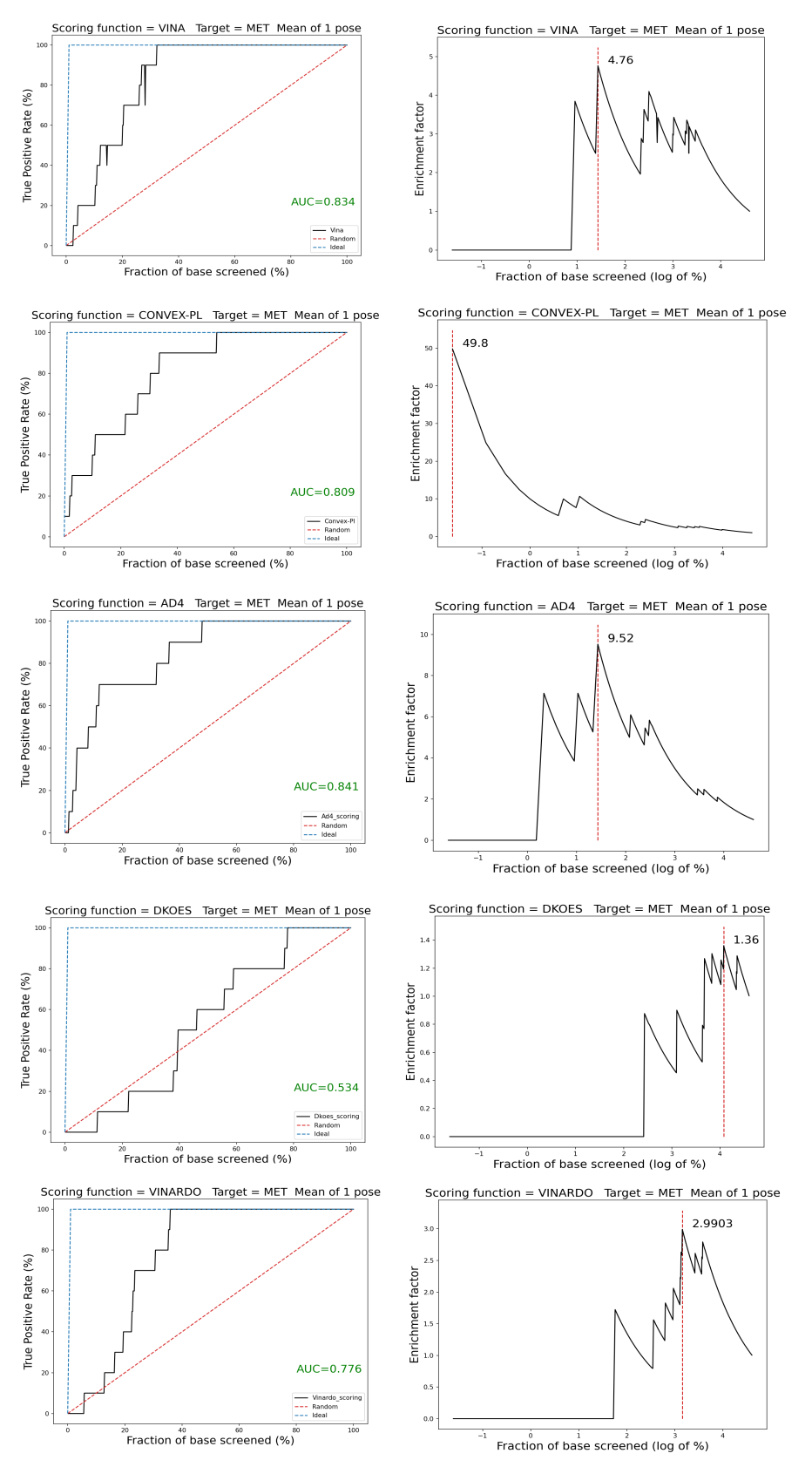


(B)


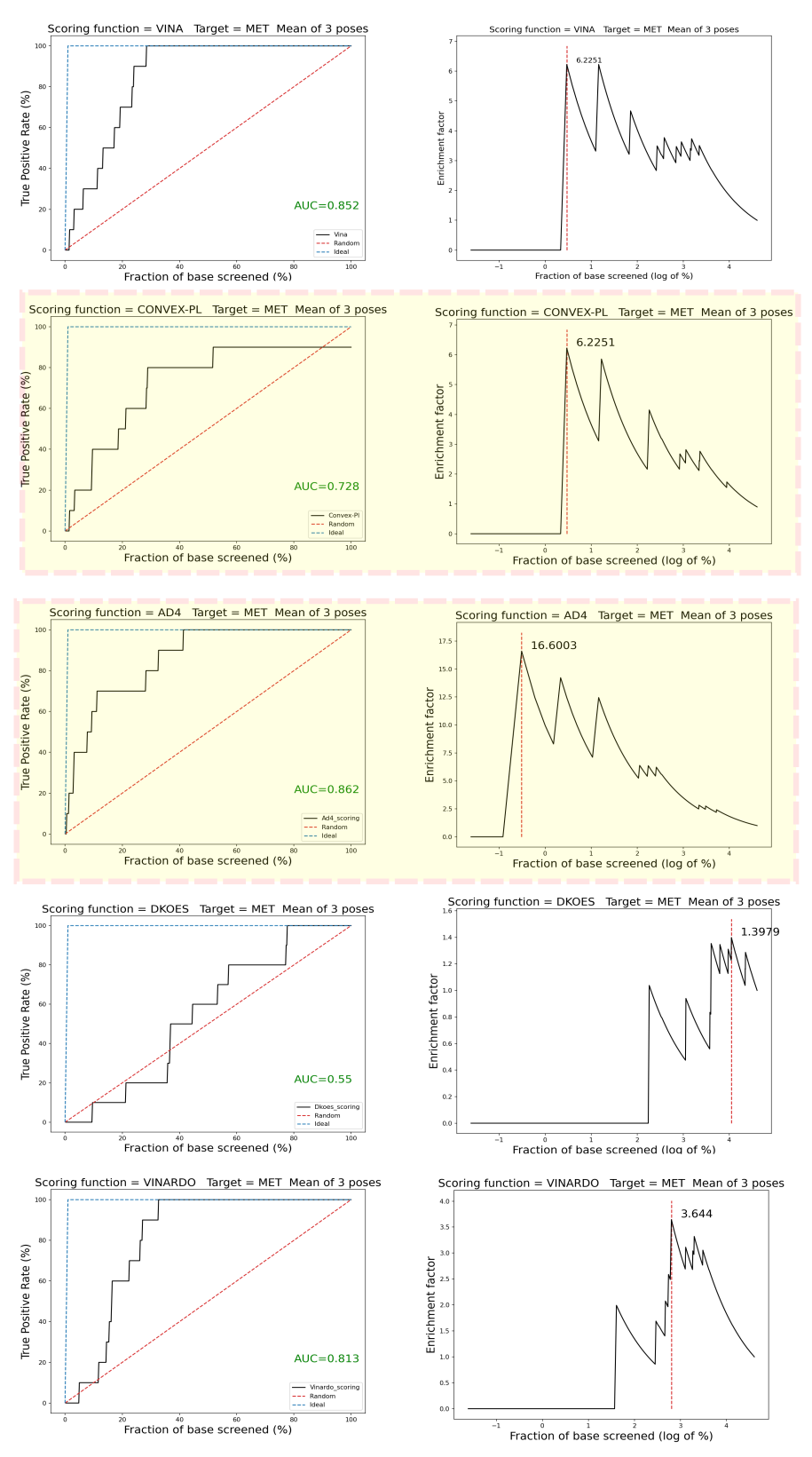


*Score function selected to perform forward virtual screening analysis is highlighted in yellow.*

**Figure S19. ROC and Enrichment curves considering true positive ligands and decoys ligands for the Agonist γ-Aminobutyric acid Receptor type A receptor (GABAaR). (A) Curve produced using only the highest affinity score for different affinity score functions. and (B) Curve produced using the three high-affinity scores for different affinity score functions. The area under the ROC curve is denoted in green by the initials AUC and the selected scoring functions for the screening analyses are highlighted in yellow.**

(A)


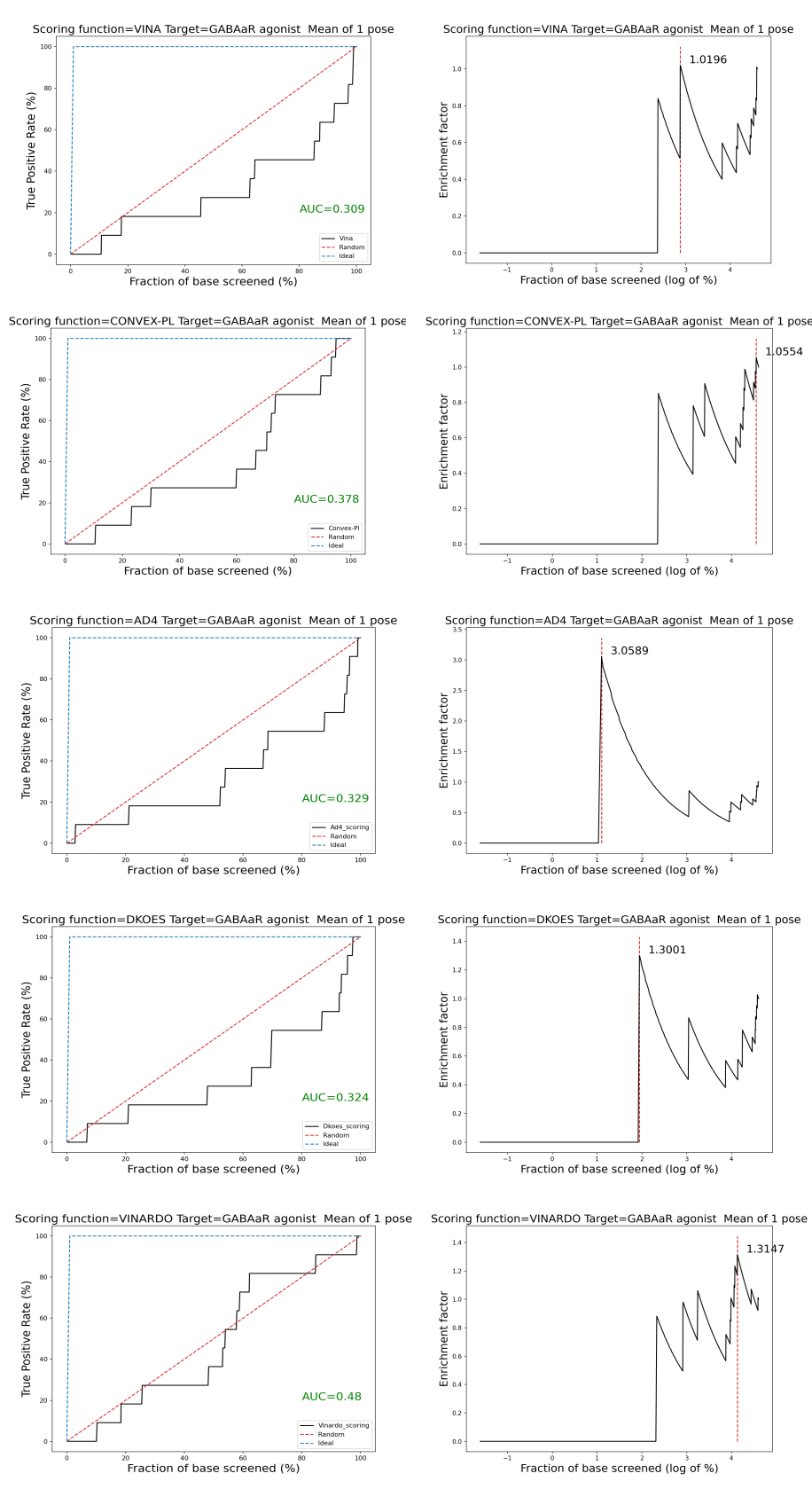


(B)


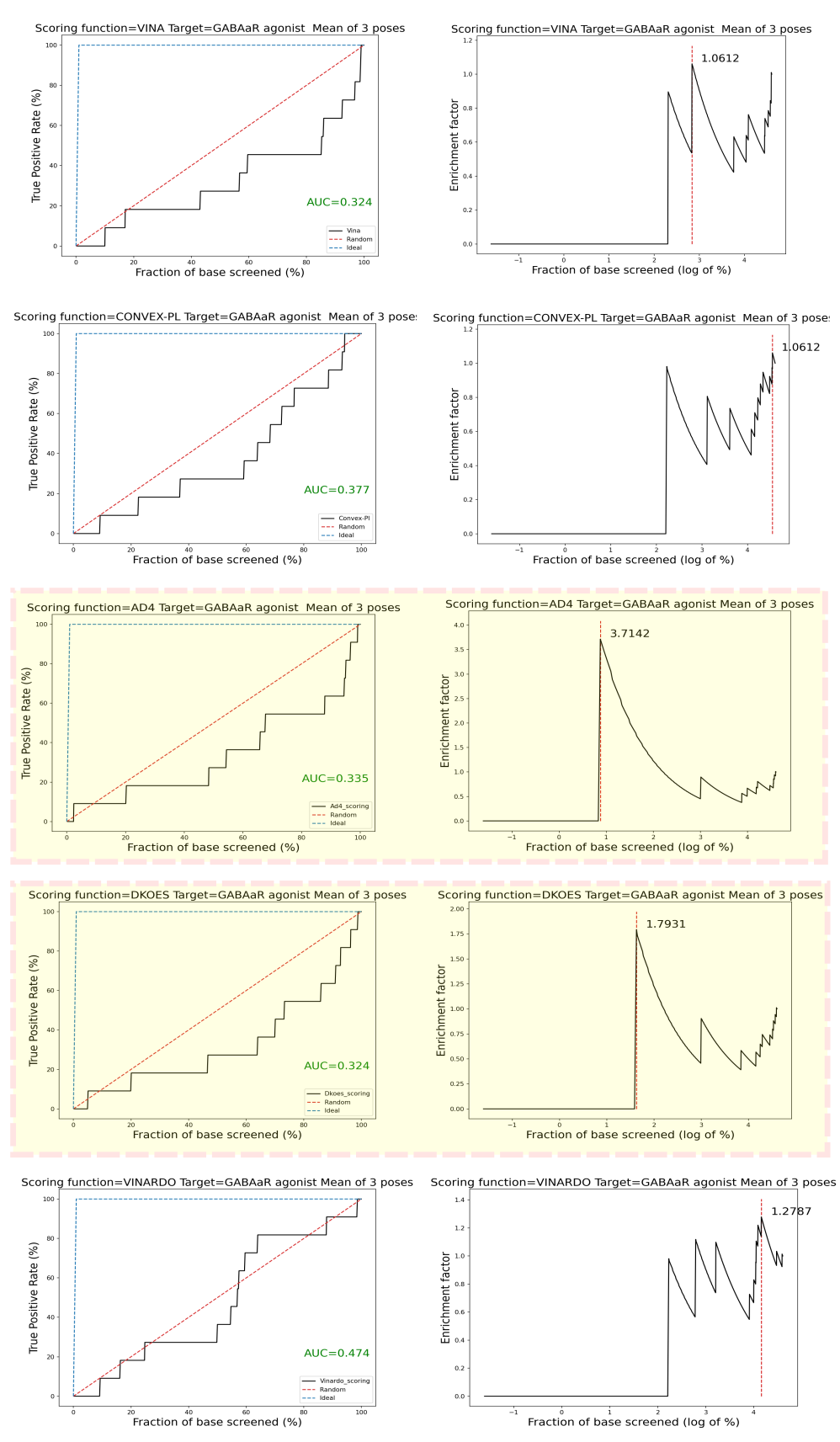
*Score function selected to perform forward virtual screening analysis is highlighted in yellow.*

**Figure S20. ROC and Enrichment curves considering true positive ligands and decoys ligands for the Antagonist γ-Aminobutyric acid Receptor type A receptor (GABBAa). (A) Curve produced using only the highest affinity score for different affinity score functions. and (B) Curve produced using the three high-affinity scores for different affinity score functions. The area under the ROC curve is denoted in green by the initials AUC and the selected scoring functions for the screening analyses are highlighted in yellow.**

(A)


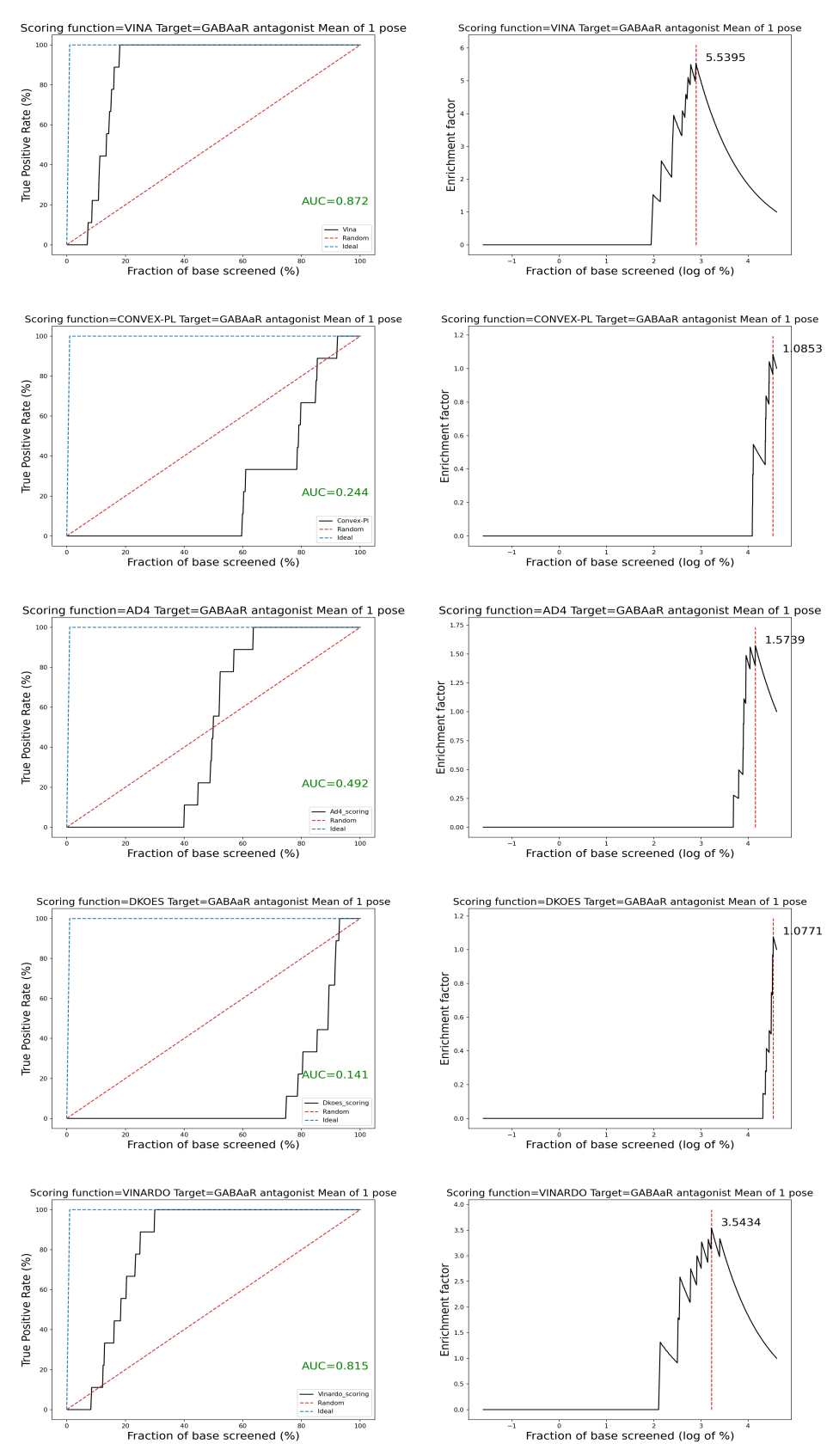


(B)


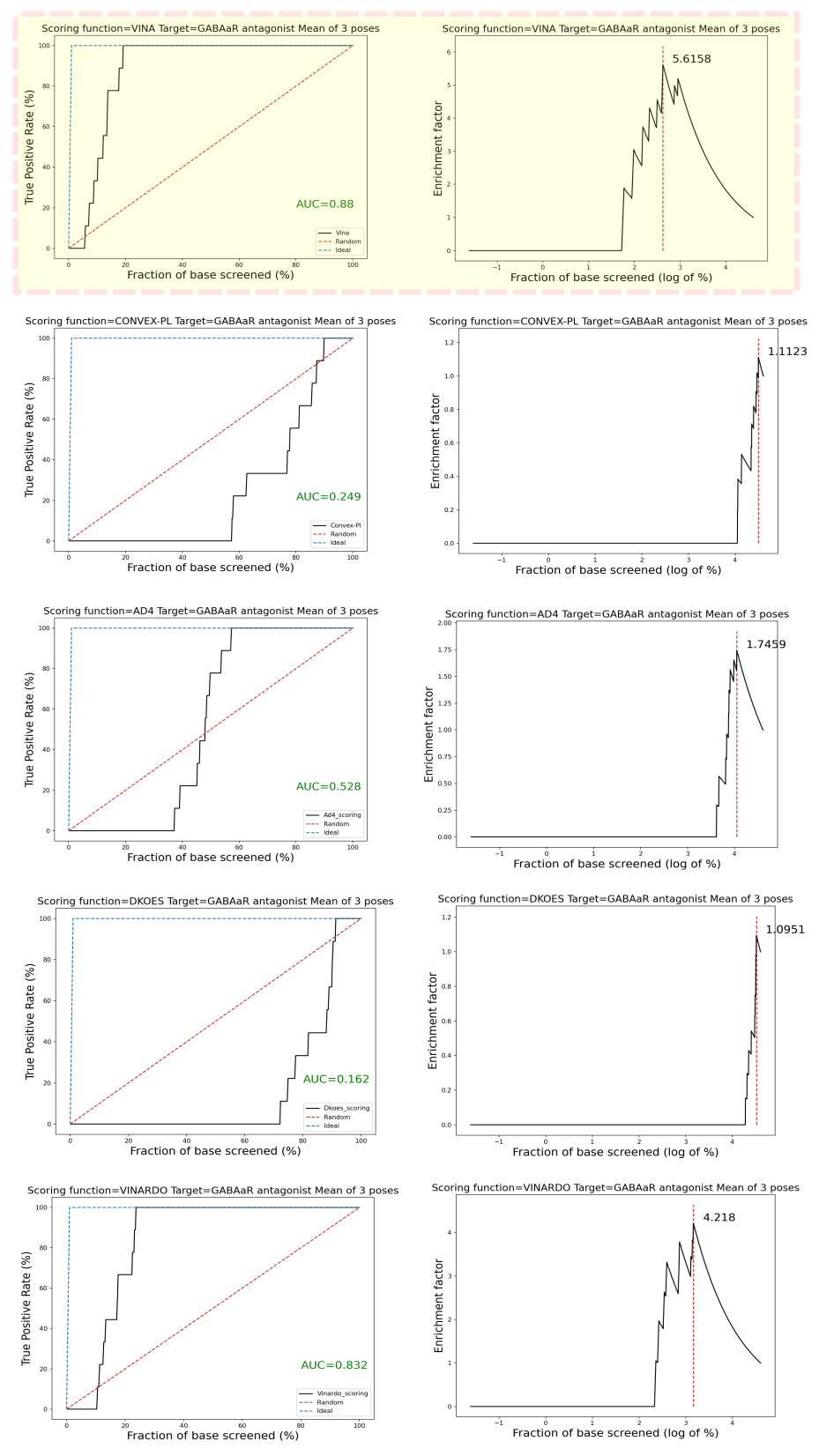


*Score function selected to perform forward virtual screening analysis is highlighted in yellow.*

**Figure S21. ROC and Enrichment curves considering true positive ligands and decoys ligands for the Agonist Octopamine Receptor (OctpR). (A) Curve produced using only the highest affinity score for different affinity score functions. and (B) Curve produced using the three high-affinity scores for different affinity score functions. The area under the ROC curve is denoted in green by the initials AUC and the selected scoring functions for the screening analyses are highlighted in yellow.**

(A)


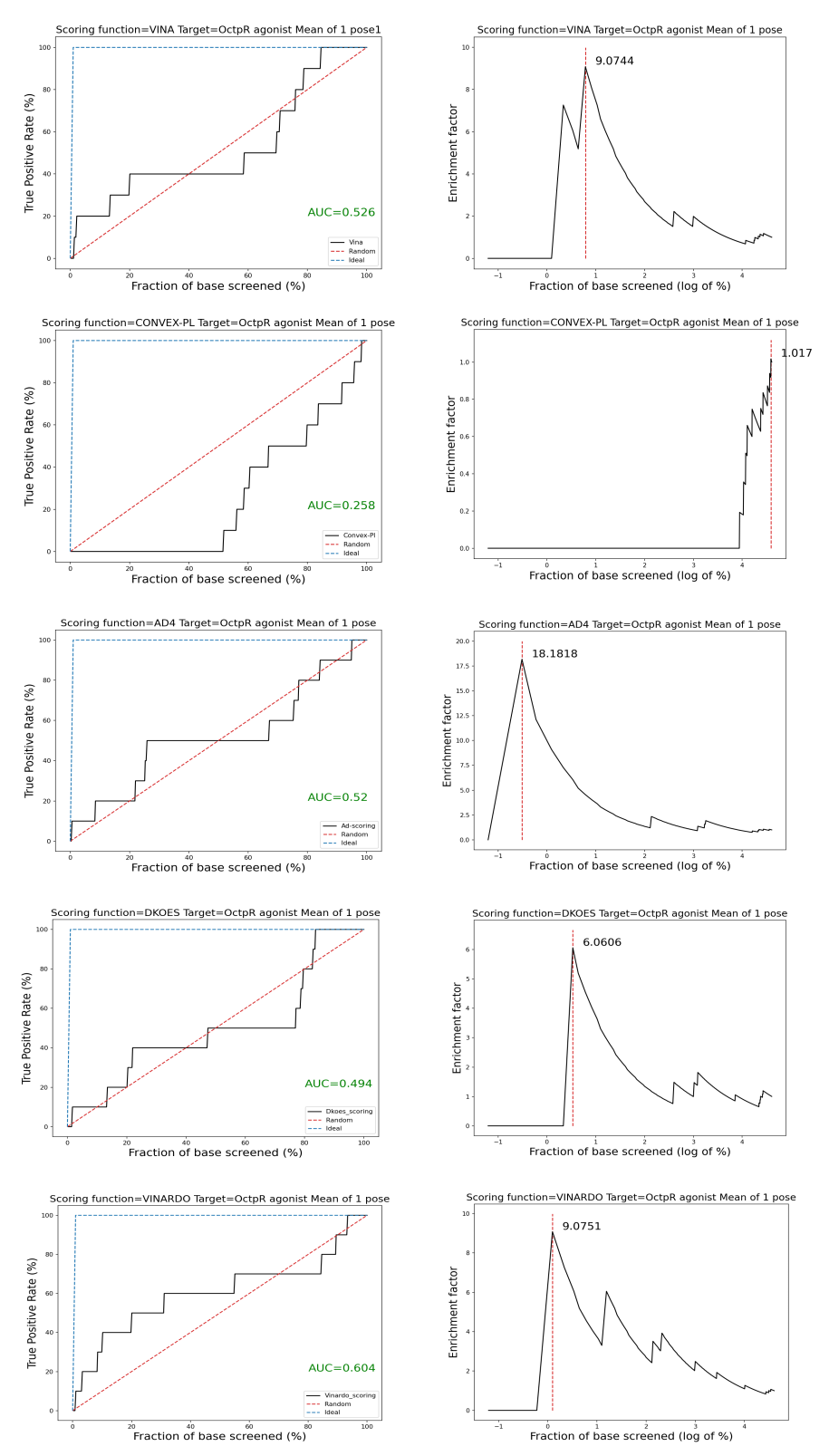


(B)


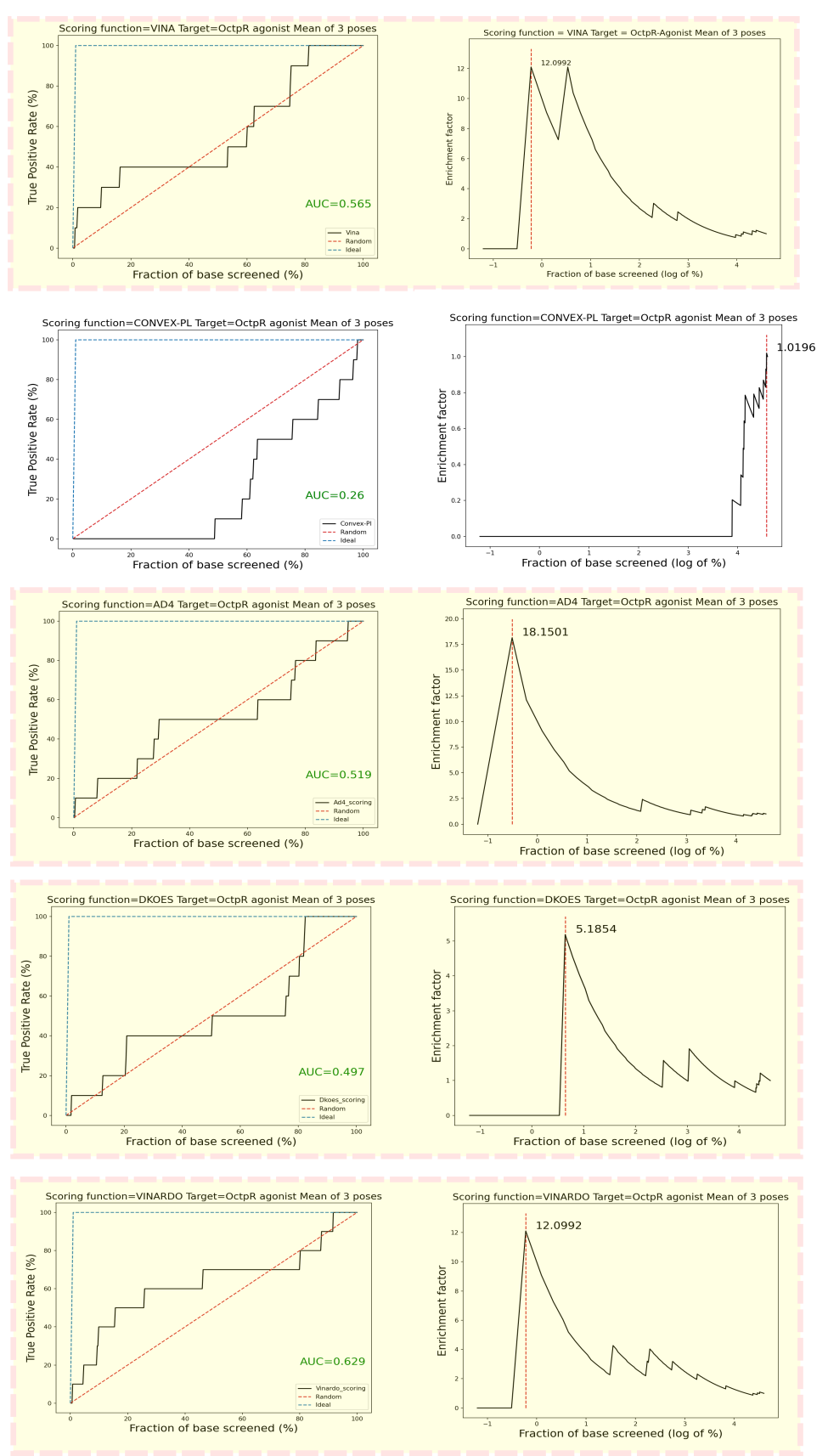


*Score function selected to perform forward virtual screening analysis is highlighted in yellow.*

**Figure S22. ROC and Enrichment curves considering true positive ligands and decoys ligands for the Antagonist Octopamine Receptor (OctpR). (A) Curve produced using only the highest affinity score for different affinity score functions. and (B) Curve produced using the three high-affinity scores for different affinity score functions. The area under the ROC curve is denoted in green by the initials AUC and the selected scoring functions for the screening analyses are highlighted in yellow.**

(A)


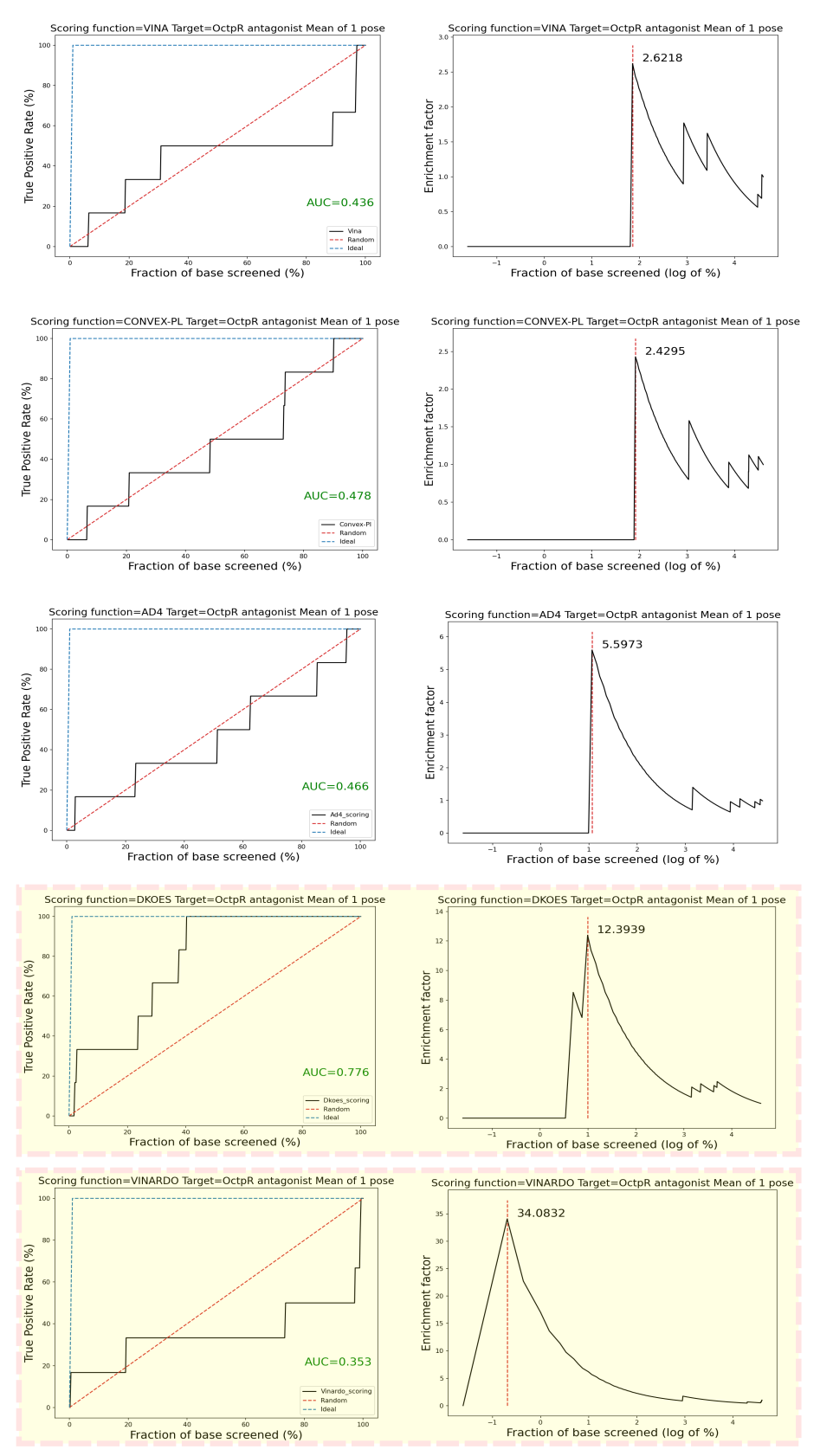


*Score function selected to perform forward virtual screening analysis is highlighted in yellow.*

(B)


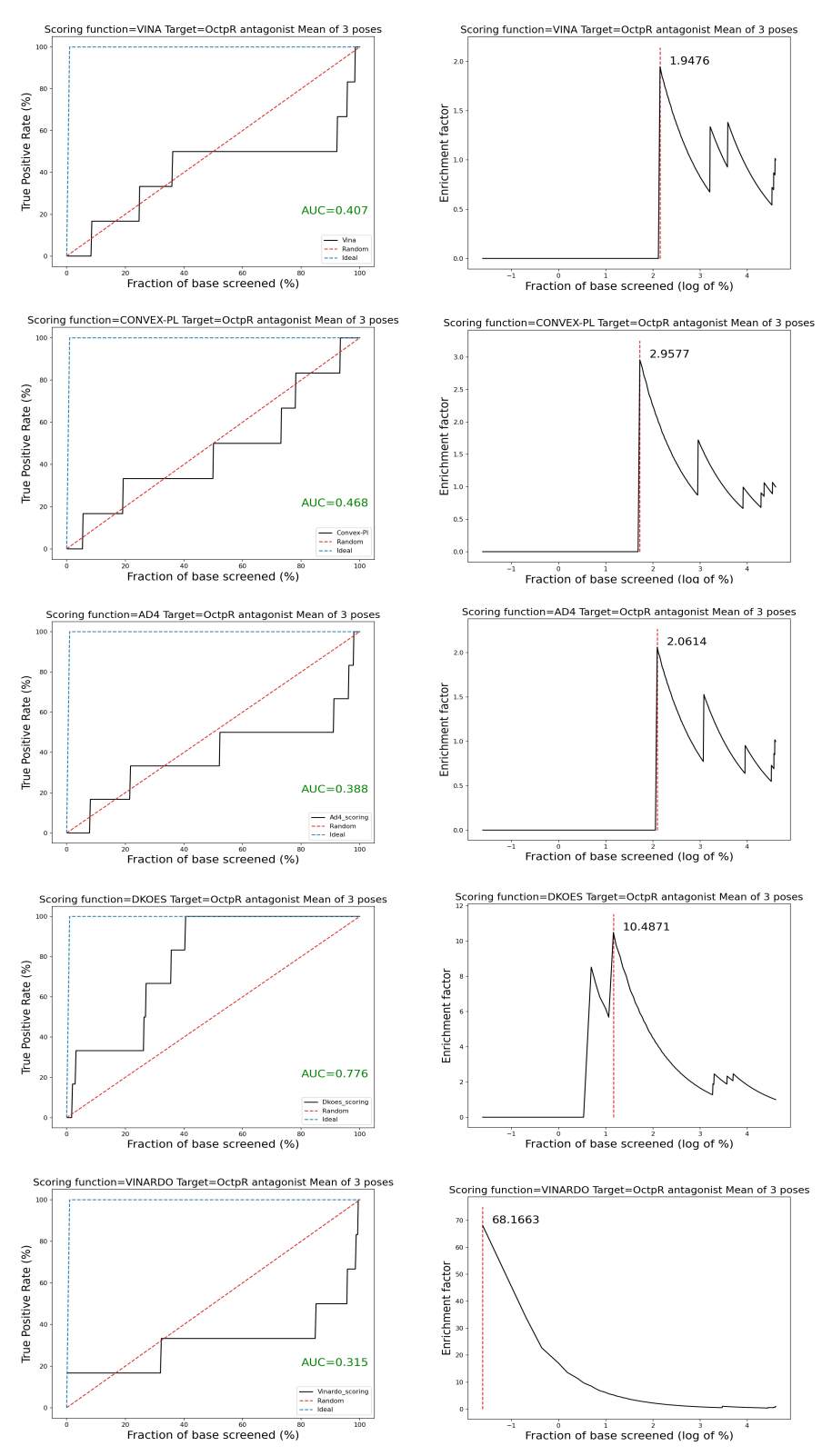


**Figure S23. The plot of the Number of Clusters generates hierarchical clustering and the Tanimoto Cutoff input. The cutoff value of the first plateau generates the 11 Clusters chosen.**


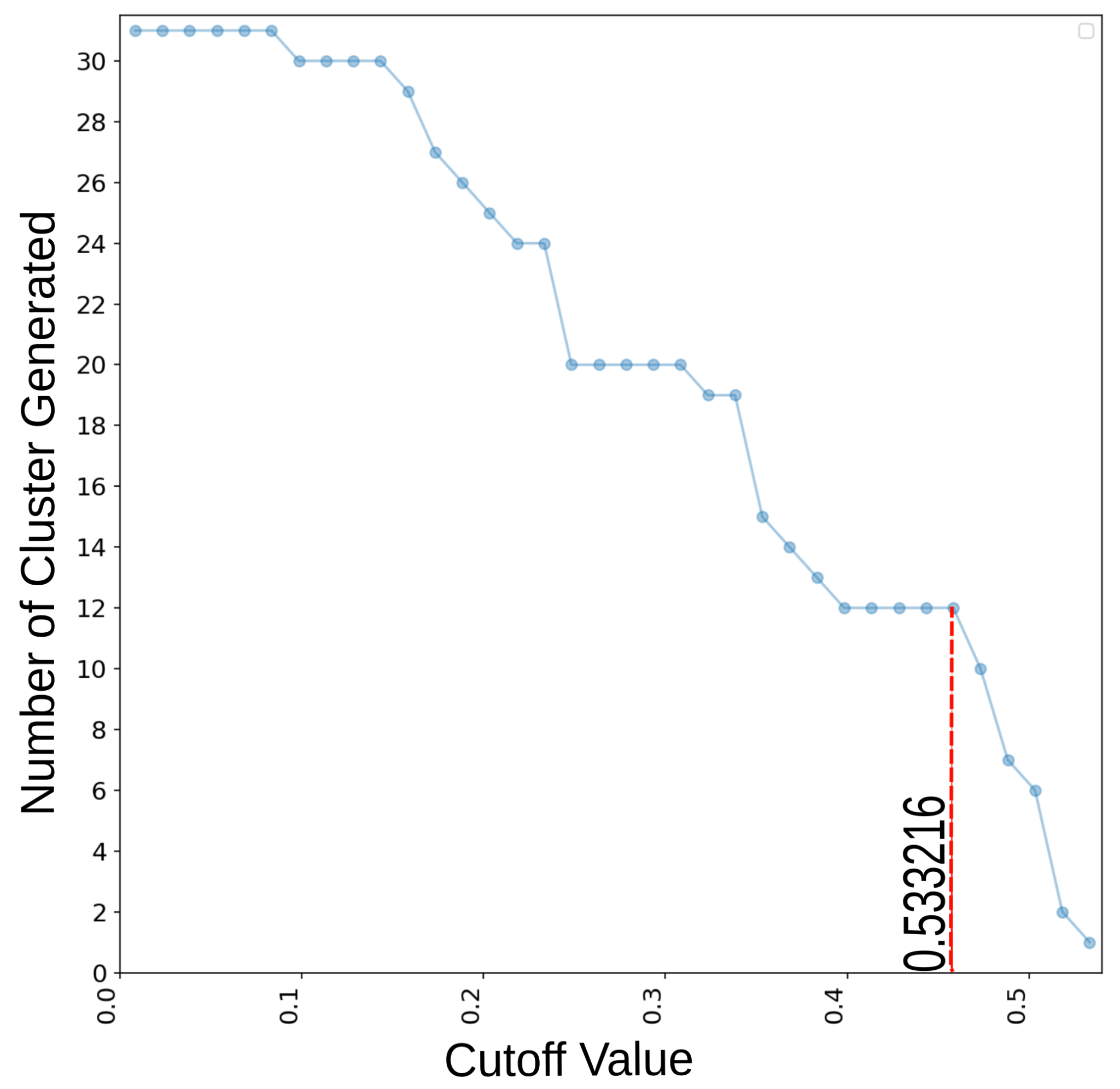


##

##

**Figure S24. Correlation among Smina re-score functions. It is an experimental docking test to evaluate the similarity and dissimilarity among score functions. The Dk_scoring was eliminated because it is the same as Autodock Vina.**


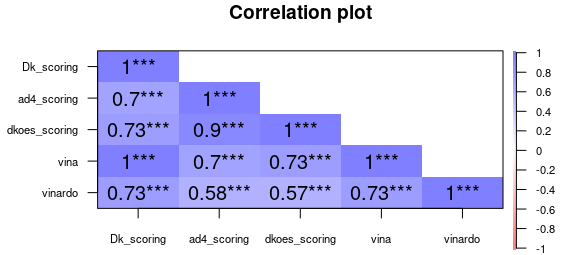


##

## **Bioassay Larval Video Analysis**

**Videos S1. Larval Motility presented by** [Videos](https://drive.google.com/drive/folders/1pA4VEjYhS-YKwWuGkoVoS-tmX-kTxWIr?usp=sharing)

**Table S11. Database ID and SMILE notation of plant natural database compounds used in method calibration.**

| **ID in Database** | **SMILE notation** |
| --- | --- |
| ZINC2034639 | CCC[C@H](C)CC |
| ZINC1531148 | CCC/C=C/C=O |
| ZINC33943706 | C=CCOC(=O)C(=O)OCCCCC |
| ZINC2242633 | CC1=C[C@H]2[C@H](CC1)C2(C)C |
| ZINC14590585 | CC[C@@H](C)C(=O)OCCC(C)C |
| ZINC967579 | CC1=CC[C@@H]2C[C@H]1C2(C)C |
| ZINC100191743 | C=C1C[C@H]2CC[C@H]1C2(C)C |
| ZINC967582 | C=C1CC[C@@H]2C[C@H]1C2(C)C |
| ZINC141880133 | CC(C)[C@@]1(O)C=C[C@@H](C)CC1 |
| ZINC967595 | CC1=CC[C@@H](C(C)(C)O)CC1 |
| ZINC967566 | CC12CCC(CC1)C(C)(C)O2 |
| ZINC14588411 | C=C1CC[C@@H](C(=C)C)C[C@@H]1O |
| ZINC5227717 | C/C=C/C(C)=O |
| ZINC34117700 | C=CCc1ccc(OCC(=O)OC)c(OC)c1 |
| ZINC1063075 | CC1=CC[C@H](CC=O)C1(C)C |
| ZINC59585923 | CC1=CC2C(CC1)C(=C)CCC2C(C)C |
| ZINC1529210 | CC(C)=CCC/C(C)=C/CO |
| ZINC1849759 | CC(C)=CCC[C@](C)(O)[C@@H]1CC=C(C)CC1 |
| ZINC59587341 | C=C(C)[C@@H]1CC[C@@]2(C)CCC=C(C)[C@@H]2C1 |
| ZINC8234293 | C=C(C)[C@@H]1CC[C@@]2(C)CCCC(=C)[C@@H]2C1 |
| ZINC59778407 | CC1=C2C=C(C(C)C)CC[C@]2(C)CCC1 |
| ZINC70450851 | C=C1CCC[C@]2(C)CCC(=C(C)C)C[C@H]12 |
| ZINC2123310 | C=C1CC[C@H]2[C@H]([C@@H]3[C@@H](C)CC[C@@H]13)C2(C)C |
| ZINC49538669 | C=C(C)[C@@H]1CCC2=CCC[C@@H](C)[C@@]2(C)C1 |
| ZINC70451257 | CC1=C[C@@H]2[C@@H](CC1)[C@H](C)C=CCC2(C)C |
| ZINC104883029 | CC1=C[C@@H]2[C@H](C(C)C)CC[C@@H](C)[C@]2(O)CC1 |
| ZINC13373002 | C=C1CC[C@H]2[C@@H]([C@@H]3[C@H]1CC[C@@]3(C)O)C2(C)C |
| ZINC104876494 | C=C(C)[C@@H]1CCC(C)=C2CC[C@H](C)[C@H]2C1 |
| ZINC2510193 | CC1(C)CCC[C@H](CC=O)C1 |
| ZINC143826668 | CC1=C(C)C[C@H]2[C@@H](CC[C@H]2C)C1 |
| ZINC139054025 | CC(=O)[C@@H]1CC[C@]2(C)[C@H](O)CC[C@@H](C)[C@H]2C1 |
| ZINC5735752 | CC(C)=C1CC[C@@H](C)CC1=O |
| ZINC8418983 | CC1=CC[C@@H](C(C)C)C=C1 |
| ZINC967513 | C=C(C)[C@H]1CC=C(C)CC1 |
| ZINC1531550 | C=C[C@@](C)(O)CC/C=C(\C)CCC=C(C)C |
| ZINC8234282 | C=C1CC/C=C(\C)CC[C@@H]2[C@@H]1CC2(C)C |
| ZINC30730221 | C=C1/C=C/[C@H](C(C)C)CC/C(C)=C/CC1 |
| ZINC100086523 | C/C1=C\[C@H]2[C@@H](CC/C(C)=C/CC1)C2(C)C |
| ZINC2019619 | CC1=CC[C@@H]2[C@@H](C1)C2(C)C |
| ZINC968230 | C=C1[C@@H]2CC[C@@H](C2)C1(C)C |
| ZINC85644544 | C=C1[C@H]2C[C@H](C[C@H]2O)C1(C)C |
| ZINC86034080 | C=C(C)[C@H]1CCC=C(C)C1 |
| ZINC968225 | CC1=CCC(=C(C)C)CC1 |
| ZINC59778864 | C=C1CC[C@@]23C[C@@H]1C(C)(C)[C@@H]2CC[C@H]3C |
| ZINC62237753 | C=C[C@@]1(C)CC[C@@H](C(=C)C)C[C@H]1C(=C)C |
| ZINC968468 | C=C1CCCC(C)(C)[C@@]12CC=C(C)CC2 |
| ZINC70454368 | C=C(C)[C@@H]1CC[C@H](C)C2=C(C1)[C@@H](C)CC2 |
| ZINC100780735 | C[C@@H]1CCC2=C1C[C@@H]1CC[C@@]2(C)C1(C)C |
| ZINC59778570 | CC1=C[C@@H]2[C@H](CC1)C(C)=CCCC2(C)C |
| ZINC64624831 | C=C1CCCC(C)(C)[C@@H]2C=C(C)CC[C@@H]12 |
| ZINC1648304 | CCCCCC(=O)OCC |
| ZINC15121415 | CC(=O)CC/C=C(/C)CCC=C(C)C |
| ZINC409176 | COC(=O)c1ccccc1OC |
| ZINC490 | COC(=O)c1ccccc1O |
| ZINC175245225 | CCCCCC(=O)O[C@@H](C)[C@@H]1CCOC1 |
| ZINC2169363 | CC(C)=C1CC[C@H](C)C2=C(C1)[C@H](C)CC2 |
| ZINC1530331 | C=CC(=C)CCC=C(C)C |
| ZINC57988166 | CC1=CC[C@@H]2[C@@H]3[C@H]1[C@@]2(C)CC[C@@H]3C(C)C |
| ZINC59200506 | CC1=C2[C@H]3[C@@H](CC[C@@H](C)[C@H]2CC1)C3(C)C |
| ZINC38139375 | C/C/1=C\CC(/C=C/C/C(=C/CC1)/C)(C)C |
| ZINC13380906 | C=C[C@@]1(C)C[C@H]2C=C(C)CC[C@H]2C(C)(C)O1 |
| ZINC57989172 | CC1=C2CC[C@@H](C)[C@H]2[C@H]2[C@@H](CC1)C2(C)C |
| ZINC8220462 | CC1=C[C@H]2[C@@H](CCC(=C2CC1)C)C(C)C |
| ZINC4081998 | C[C@@H]1CC[C@@H]2[C@@H]1[C@@H]1[C@H](CC[C@@]2(C)O)C1(C)C |
| ZINC5767672 | CC1=C[C@H]2[C@H](C(C)C)CC[C@](C)(O)[C@@H]2CC1 |
| ZINC141973482 | OC1[C@H]2[C@H]3C=C[C@H]3[C@H]1[C@@H]1CC[C@@H]2C1 |
| ZINC85599405 | CC1=CCC[C@@]2(C)CCC(=C(C)C)C[C@H]12 |
| ZINC1851022 | CC1=C(C(CC=C1)(C)C)C=O |
| ZINC967583 | C=C1C(=O)C[C@H]2C[C@@H]1C2(C)C |
| ZINC3861087 | C/C(=C\CO)CCC[C@H](C)CCC[C@H](C)CCCC(C)C |
| ZINC1686990 | CCCCCCCCC=O |
| ZINC100782219 | C=C1CC[C@@H]2C(=C)CC[C@H](C(C)C)[C@@H]2C1 |
| ZINC388674 | C=CCc1ccc(OC)c(OC)c1 |
| ZINC13429400 | CC1=CC[C@](O)(C2CC2)CC1 |
| ZINC968099 | CC1(C)[C@@H]2CC[C@@]1(C)[C@H](O)C2 |
| ZINC59587780 | C[C@@H]1CC[C@@H]2[C@H]1[C@H]1[C@@H](C[C@@H]3O[C@@]32C)C1(C)C |
| ZINC15120743 | C=C[C@](C)(O)C/C=C\C(=C)C |
| ZINC1531610 | CC(=CCC/C(=C/COC(=O)C)/C)C |
| ZINC967594 | CC1=CC=C(C(C)C)CC1 |
| ZINC1411 | C=CCc1ccc(O)c(OC)c1 |
| ZINC70451264 | C=C(C)[C@@H]1CC[C@]2(C)C[C@H](O)CC(C)=C2C1 |
| ZINC85880213 | CC1=CC2=C(C(C)C)CC[C@@H](C)[C@@H]2CC1 |
| ZINC64634151 | CC1=CC[C@@H]2C(=C1)[C@@H](C(C)C)CC[C@H]2C |
| ZINC32142970 | C=C1C[C@@H](C#N)CC1(C)C |
| ZINC100199761 | CC(C)[C@@H]1CC[C@@]2(C)[C@H]3C[C@@H]4[C@H]([C@H]13)[C@]42C |
| ZINC90734947 | C=C[C@]1(C)CCCC(=C)C[C@@H]1C=C(C)C |
| ZINC201364957 | C12=C3[C@@H]4C1=C1[C@@H]2[C@H]3[C@H]14 |
| ZINC1531601 | C[C@@H](CCC=C(C)C)CCO |
| ZINC1529208 | CC(=CCC/C(=C/C=O)/C)C |
| ZINC100028042 | CC(=O)O[C@H]1C[C@H]2CC[C@@]1(C)C2(C)C |
| ZINC100155602 | CCC(=O)O[C@@H]1C[C@H]2CC[C@@]1(C)C2(C)C |
| ZINC142456176 | C=C(C)[C@@H]1CCC(=C)[C@@H]2CC[C@@H](C)[C@H]2C1 |
| ZINC1531621 | C=C1C=C[C@@H](C(C)C)CC1 |
| ZINC1531619 | CC(=CC/C=C(/C)\C=C)C |
| ZINC1529819 | C=C[C@@](C)(O)CCC=C(C)C |
| ZINC56874358 | C=C1CC[C@@H]2[C@H](C)CC[C@@H]3[C@H]([C@H]12)C3(C)C |
| ZINC59206468 | C=C1CC[C@H]2[C@H]3[C@@H]1[C@]2(C)CC[C@@H]3C(C)C |
| ZINC1846611 | C=C(CCC=C(C)C)[C@@H]1CC=C(C)CC1 |
| ZINC83260318 | CC1=C[C@@H]2[C@H](CC1)C(C)=CC[C@@H]2C(C)C |
| ZINC58257 | CCOC(=O)CCc1ccccc1 |
| ZINC8234296 | CC(C)=CCC[C@H](C)[C@@H]1C=CC(C)=CC1 |
| ZINC85664165 | C=C[C@@]1(C)CCC(=C(C)C)C=C1C(C)C |
| ZINC100232131 | CC1=CC[C@]23[C@@H](C)CC[C@H](C(C)C)[C@H]2[C@@H]13 |
| ZINC70454426 | C/C=C(C)\C=C/C=C(C)C |
| ZINC32166631 | C[C@H]1CC[C@@H]2[C@@H]1[C@@H]1[C@@H](CC[C@@]23CO3)C1(C)C |
| ZINC896628 | CC(=O)c1ccccc1 |
| ZINC2600024 | CCCCCCCCC#CCCCCCCCC |
| ZINC137919391 | CC(C)=C1CC=C2CC[C@@H](O)[C@](C)(CC1)C2 |
| ZINC111473060 | CC1=C[C@H]2CC[C@@]1(C)C2(C)C |
| ZINC195760538 | CCCC/C=C/C#CC(C)(C)C |
| ZINC165056935 | C=C(C)CCC/C(C)=C\C(C)=O |
| ZINC348140 | COc1ccc(C(C)(C)C)cc1OC |
| ZINC32176608 | C#C[C@H](C)OC(=O)c1ccc(CC)cc1 |
| ZINC33841709 | CC(=O)OCC[C@@](C)(O)CC/C=C(\C)CCC=C(C)C |
| ZINC2010172 | CC/C=C/CCOC=O |
| ZINC96334604 | C/C=C/[C@@H](C)[C@@H](C)/C=C/C |
| ZINC85867164 | C=C1C=C2[C@H](CC1)[C@@H](C)CCCC2(C)C |
| ZINC5158152 | C=CC(=C)CCC(=O)C(=C)C |
| ZINC85644689 | C=C1C(C)=CCCC1(C)C |
| ZINC230878335 | Cc1cccc2c1C=CCC2(C)C |
| ZINC32217531 | CC(C)=CC[C@H]1CC=C(C)[C@@H](C=O)C1(C)C |
| ZINC143807786 | C=C[C@]1(C)CCCC(C)=C[C@H]1C=C(C)C |
| ZINC1850881 | COC(=O)C[C@@H](C)CCC=C(C)C |
| ZINC1531600 | CC(C)=CCC[C@@H](C)CC=O |
| ZINC4102279 | C=C1[C@H](O)C[C@H]2C[C@@H]1C2(C)C |
| ZINC136696735 | CC1=CC[C@]23CCCC(C)(C)[C@H]2CC[C@]13C |
| ZINC968030 | CC1(C)[C@@H]2CC=C(C=O)[C@H]1C2 |
| ZINC59200507 | C[C@@H]1CC[C@H](C=C2[C@@H]1CC[C@H]2C)C(=C)C |
| ZINC59778978 | CC(C)[C@@H]1CC[C@@]2([C@H]1[C@@H]3[C@H]2CCC3=C)C |
| ZINC2035755 | CC1=CC[C@@]2(C(C)C)C[C@@H]12 |
| ZINC59587245 | CC1CC2C(C=C1)C2(C)C |
| ZINC1699438 | Cc1cccc(C(C)C)c1 |
| ZINC1699439 | Cc1ccccc1C(C)C |
| ZINC968246 | Cc1ccc(C(C)C)cc1 |
| ZINC968250 | Cc1ccc(C(C)C)cc1 |
| ZINC967593 | CC1=CC=C(C(C)C)CC1 |
| ZINC967520 | CC1(C)[C@@H]2CC[C@@]1(C)C(=O)C2 |
| ZINC967800 | CC1=CC[C@@H](C(C)(C)O)CC1 |
| ZINC14588455 | C=C(C)[C@H]1CC=C(C)C(=O)C1 |
| ZINC4098372 | CC(=C)[C@@H]1C[C@@H]2[C@@](O2)(C(=O)C1)C |
| ZINC12153091 | CC1=CC[C@@H](CC1=O)C(C)C |
| ZINC2508248 | C[C@H](CCCC(=C)C)CC=O |
| ZINC968287 | CC(=C)[C@@H]1CCC2(C(C1)O2)C |
| ZINC967511 | CC(C)[C@H]1CC[C@H](C)C[C@@H]1O |
| ZINC2559334 | CC1([C@H]2CC[C@H]([C@@H]1C2)CO)C |
| ZINC968028 | CC1([C@H]2CC=C([C@@H]1C2)CO)C |
| ZINC967597 | Cc1ccc(C(C)C)c(O)c1 |
| ZINC4262096 | CC1=CC[C@](CC1)(C(C)C)O |
| ZINC967599 | CC1=CC(=O)[C@@]2([C@H]1C2)C(C)C |
| ZINC967601 | CC1=CC(=O)[C@H]2C[C@@H]1C2(C)C |
| ZINC4410593 | CC1=C[C@@H]([C@H]2C[C@@H]1C2(C)C)O |
| ZINC967816 | C=C(C)[C@@H]1CC[C@@H](C)C(=O)C1 |
| ZINC100075761 | C[C@H]1CC[C@@H](C(=O)C1)C(=C)C |
| ZINC59586886 | CC(=CCC/C(=C/CCC(=C)C=C)/C)C |
| ZINC30731544 | CC1=CC/C(=C(/C)\CCC=C(C)C)/CC1 |
| ZINC238399989 | C[C@@H]1CCC=C2CC[C@H]3[C@@H](C3(C)C)[C@@]21C |
| ZINC968471 | CC(C)=CCC[C@@H](C)c1ccc(C)cc1 |
| ZINC2018831 | CC1=CC[C@](CC1)([C@@H](C)CCC=C(C)C)O |
| ZINC2083320 | C[C@@]12CC[C@@H]3[C@H](CC3(C)C)C(=C)CC[C@H]1O2 |
| ZINC4098262 | C[C@]12CCCC(=C)[C@@H]1C[C@@H](CC2)C(C)(C)O |
| ZINC1996067 | C[C@H]1CC[C@H](CC2=C1CC[C@@H]2C)C(C)(C)O |
| ZINC6071066 | CC1=CC=C(C=C1)[C@@H](C)CC(=O)C=C(C)C |
| ZINC12358735 | C/C=C/C1=CC=C(C=C1)OC |
| ZINC12358780 | COC(=O)/C=C/C1=CC=CC=C1 |
| ZINC967635 | C=CCc1ccc(OC)cc1 |
| ZINC1676040 | C[C@@]12CCCC([C@H]1CCC(=C)[C@H]2CC[C@](C)(C=C)O)(C)C |
| ZINC5158074 | CC#CC#C/C=C/1\C=CC2(O1)CCCO2 |
| ZINC1529247 | CCCCCCCCCCO |
| ZINC2512204 | CCCCc1ccc2c(c1)OCO2 |
| ZINC1653216 | CCCCCCCC(C)=O |
| 3059 | CC(C)[C@H]1CC[C@H](C)C[C@@H]1O |
| 3066 | CC(C)[C@@H]1CCC(=C)[C@H](O)[C@H]1O |
| 3067 | CC(C)[C@@]1(O)CCC(C)=CC1 |
| 3069 | C[C@@H]1CC[C@H](CC1=O)C(C)=C |
| 3070 | CC(C)[C@H]1CC[C@@]2(C)O[C@H]2C1=O |
| 3087 | C[C@H]1CC[C@@H]([C@H](O)C1)C(C)(C)O |
| 3089 | CC1=CC[C@H](CC1)C(C)(C)O |
| 3186 | CC1(C)[C@H]2CC[C@](C)(C2)C1=O |
| 3189 | CC(C)=CCC[C@](C)(OC(C)=O)C=C |
| 3190 | CC(C)[C@H]1CCC(C)=CC1=O |
| 3191 | CC(C)=C1CCC(C)=CC1=O |
| 3193 | C[C@H](CCC=C(C)C)CC=O |
| 3195 | CC(C)=CCC\C(C)=C\CO |
| 3197 | CC(C)=CCC\C(C)=C\C=O |
| 3205 | C[C@@H](CCO)CCC=C(C)C |
| 3206 | C[C@@H](CCOC(C)=O)CCC=C(C)C |
| 3208 | CC(C)=CCC\C(C)=C\COC(C)=O |
| 3212 | CC1=C(C=O)C(C)(C)CCC1 |
| 3215 | CC(=O)OCC1=CC[C@H]2C[C@@H]1C2(C)C |
| 3217 | CC1(C)[C@@H]2C[C@H]1[C@@H](CO)CC2 |
| 3220 | CCO[C@@H]1OC[C@@H]2CC(=O)CC(C)(C)[C@H]12 |
| 3227 | CC1=C2CCC(C)=C[C@@H]2OC1 |
| 3233 | CC(C)(O)[C@H]1CC[C@@](C)(O1)C=C |
| 3324 | C\C(Cl)=C/C[C@H](O)C(\C)=C\Cl |
| 3372 | COC1=CC(\C(CO)=C\Cl)=C(O)C=C1C |
| 3408 | CC(C)=CC[C@@H](CO)C(C)=C |
| 3409 | CC(C)=CC[C@@H](COC(C)=O)C(C)=C |
| 3410 | CC(=O)O[C@H]1C[C@H]2CC[C@]1(C)C2(C)C |
| 3415 | CC1(C)[C@H]2CC[C@]1(C)[C@](C)(O)C2 |
| 3416 | CC(C)=C[C@H](OC(C)=O)C(C)(C)C=C |
| 3417 | CC(=O)OC(C)(C)[C@H](C=C)C(=O)C(C)=C |
| 3444 | CC(=C)[C@@H]1CCC(CO)=CC1=O |
| 3493 | CC(=O)C1=C(O)C=C(C)C=C1 |
| 3494 | CC(=C)[C@@H](C=C)\C=C(/C)CO |
| 3495 | CC(=O)OC\C(C)=C\[C@@H](C=C)C(C)=C |
| 3496 | CC(C)=C[C@H](O)C(C)(C)C=C |
| 3555 | CC(C)[C@@]12C[C@@H]1C(C)=CC2=O |
| 3561 | CC(C)[C@]12C[C@H]1[C@@H](C)[C@H](O)C2 |
| 3629 | CC(C)[C@H]1CC[C@@H](C)CC(=O)O1 |
| 3630 | CC(C)=C[C@H](C=C)C(C)(C)O |
| 3731 | CC1(C)[C@H]2C[C@@H]1C(=C)[C@H](O)C2 |
| 3761 | CC1(C)[C@@H]2C[C@H]1[C@H](CC2)C=O |
| 3779 | CC(=O)O[C@H]1C=C(C)[C@@H]2C[C@H]1C2(C)C |
| 3844 | C[C@H]1CC[C@@H](C[C@@H]1OC(C)=O)C(C)=C |
| 4034 | CC(=C)[C@H]1CC[C@@](C)(O1)C=C |
| 4229 | C[C@H](O)[C@@H]1O[C@H]1[C@@H]1C(C)=CCCC1(C)C |
| 4394 | C[C@@H]1[C@H](C=C)[C@@H](OC1=O)C(C)=C |
| 4400 | CC(C)=CCC[C@](O)(CO)C=C |
| 4420 | CC(C)=CC[C@]1(O)OCC=C1C |
| 4468 | C\C=C\C(=O)C1=C(C)[C@H](O)CCC1(C)C |
| 4470 | CC(C)(C=C)C(=O)[C@H]1OC1(C)C |
| 4471 | CC(=C)[C@@H](O)[C@@H](O)C(C)(C)C=C |
| 4472 | CC(C)=C[C@@H](C=C)C(=C)COC(C)=O |
| 4483 | CC(=C)[C@@H]1CC=C(C)[C@H](O)C1 |
| 4485 | CC(=O)OC(C)(C)[C@H](C=C)[C@H](O)C(C)=C |
| 4498 | CC(=O)O[C@H]1C[C@@H]2C[C@H](C1=C)C2(C)C |
| 4558 | CC1=CC(O)=C(C=C1)C(=C)CO |
| 4561 | CC(C)=C\C=C\C1=CC(=O)OC1 |
| 4589 | CC(=O)O[C@@](C)(CC\C=C(/C)C=O)C=C |
| 4590 | CC(C)CC(=O)C[C@H](C)C=C |
| 4591 | CC(C)CC(=O)\C=C(/C)C=C |
| 4611 | CC(=O)OC(C)(C)[C@@H]1CC[C@@](C)(O1)C=C |
| 4612 | C[C@@H](CO)[C@H]1CC[C@@](C)(O1)C=C |
| 4641 | CC(=C)[C@H]1CCC(=C)[C@@H](O)C1 |
| 4642 | CC(=O)O[C@H]1C[C@@H](CC=C1C)C(C)=C |
| 4654 | CC(C)[C@H]1C[C@H](O)C(C)=C[C@@H]1O |
| 4660 | CC(C)C(=O)OC1=CC(C)=CC=C1[C@]1(C)CO1 |
| 4676 | CC(C)C1=CC(O)=C(C=O)C=C1 |
| 4730 | CC(C)=CC(=O)\C=C(/C)C=C |
| 4757 | CC1=CC=C2C(=C)[C@H](O)OC2=C1 |
| 5000 | C\C(CO)=C/CCC(=C)C=C |
| 5043 | C[C@]12C[C@@H](O)CC(C)(C)C1=CC(=O)O2 |
| 5050 | CC1(C)O[C@@](C)(CCC1=O)C=C |
| 5150 | CC(C)[C@H]1CC[C@](O)(CO)C=C1 |
| 15528 | O=C1CC2CCCCC2O1 |
| 15849 | [H][C@]12CCC=C(CO)[C@]1(C)CC[C@@H](C)[C@]2(C)CCC(C)=O |
| 16622 | C[C@@H]1C[C@H]2OC(=O)[C@@]3(C)CCC[C@@](C)(C23)[C@@]11CCC(=O)O1 |
| 17210 | C[C@H]1COC2=C1C(=O)OC1=C2C=CC2=C(C)C=CC=C12 |
| 17289 | C[C@@H]1COC2=C1C(=O)C1=C(C2=O)C2=C(C=C1)C(C)=CC=C2 |
| 17294 | C[C@H]1COC2=C1C(=O)C(=O)C1=C2C=CC2=C(C)C=CC=C12 |
| 17296 | C[C@@H]1COC2=C1C(=O)C(=O)C1=C2C2=C(C=C1)C(C)=CC=C2 |
| 17451 | CC1COC2=C1C(=O)C(=O)C1=C2C=CC2=C1CCC=C2C |
| 17456 | C[C@@H]1COC2=C1C(=O)C1=C(C2=O)C2=C(C=C1)C(C)(C)CCC2 |
| 17464 | [H][C@@]12C[C@@H]3OC[C@@]1(CCCC2(C)C)C1=CC(=O)C(C)=C[C@@]31O |
| 17474 | [H][C@@]1(OC(=O)C2=C1C=CC1=C(C)C=CC=C21)[C@@]1([H])[C@H](C)COC1=O |
| 17498 | CC1COC2=C1C(=O)C1=C(OC2=O)C2=C(C=C1)C(C)=CC=C2 |
| 17554 | CC1(C)C[C@H](O)C[C@@]2(C)C1CC[C@@]1(C)OC(=O)CCC21 |
| 17619 | [H][C@@]1(OC(=O)C2=C1C=CC1=C(C)C=CC=C21)[C@]1([H])[C@@H](C)COC1=O |
| 17622 | [H]C([H])=C1C2=C(C3=C(C([H])=C2[H])C2=C(C(=O)C3=O)[C@@]([H])(C([H])([H])[H])C([H])([H])O2)C([H])([H])C([H])([H])C1([H])[H] |
| 17760 | [H][C@@]12[C@@H](O)C[C@@]3(C)OC(=O)CC[C@@H]3[C@@]1(C)CCCC2(C)C |
| 17761 | [H][C@@]12[C@@H](O)C[C@@]3(C)OC(=O)C[C@@H]3[C@@]1(C)CCCC2(C)C |
| 17767 | CC(CO)C1=CC2=C(OC1=O)C1=CC=CC(C)=C1C=C2 |
| 18443 | [H]O[C@]1([H])C([H])([H])C([H])([H])[C@]2(C([H])([H])[H])[C@]3([H])C(C([H])=C([H])[C@@]2([H])C1(C([H])([H])[H])C([H])([H])[H])=C([H])C(=O)C([H])([H])C3([H])[H] |
| 18444 | [H]O[C@]1([H])C([H])([H])C([H])([H])[C@]2(C([H])([H])[H])[C@]3([H])C(=C([H])C(=O)C([H])([H])C3([H])[H])C([H])([H])C([H])([H])[C@@]2([H])C1(C([H])([H])[H])C([H])([H])[H] |
| 19330 | [H][C@]12CC[C@](C)(C=C)C=C1[C@H](O)C[C@]1([H])C2CCC[C@@]1(C)CO |
| 19836 | CC(C)=CCCC(C)(O)C=C |
| 19837 | CC1(C)C2CC1C(CO)=CC2 |
| 19848 | CC(C)=CCC\C(C)=C\C(O)=O |
| 19898 | CC1=CC2=C(C=C1)C(C)(O)CCC2=O |
| 19902 | C[C@@H]1CC[C@@H]2CC3=C(C)C(=O)C[C@]13C2(C)C |
| 19915 | CC1(C)C2CCC(=C)C3CCC(C)(O)C3C12 |
| 19961 | CC12CCC(CC1)C(C)(C)O2 |
| 19981 | CC1=CCC(CC1)C(C)(C)O |
| 19985 | CC1=CC=C(C=C1)[C@]1(C)CC[C@@H](OO1)C(C)(C)O |
| 19986 | [H]\C(=C(\[H])C1=CC=CC=C1)[C@H]1CC=CC(=O)O1 |
| 20005 | CC1(C)C2CCC1(C)C(O)C2 |
| 20006 | CC(=O)O[C@@H]1C[C@@H]2CC[C@@]1(C)C2(C)C |
| 20008 | CC1(C)C2CCC1(C)C(=O)C2 |
| 20010 | CC(=C)C1CC=C(C)C(=O)C1 |
| 20012 | CC(C)=CCCC(C)(OC(C)=O)C=C |
| 20013 | CC(C)[C@@H]1CC[C@@H](C)CC1=O |
| 20072 | CC(=O)OC1CC2CCC1(C)C2(C)C |
| 20075 | CC1(C)C2CC1C(C=O)=CC2 |
| 20077 | CC1(C)C2CC1C(=C)C(=O)C2 |
| 20097 | C[C@@H]1CC[C@@H]2[C@@H]1[C@H]1[C@@H](CC[C@]2(C)O)C1(C)C |
| 20098 | CC1CC[C@@H]2[C@@H]1[C@H]1[C@@H](CC[C@@]2(C)O)C1(C)C |
| 20103 | [H]C12C3C(CC[C@](C)(O)C1([H])CCC2C)C3(C)C |
| 20111 | CC(CCC=C(C)C)CC=O |
| 20112 | CC(CCO)CCC=C(C)C |
| 20113 | CC(CCOC(C)=O)CCC=C(C)C |
| 20120 | C[C@@H]1CC[C@H]([C@H](O)C1)C(C)=C |
| 20121 | C[C@@H]1CC[C@H]2[C@@H]1[C@H]1[C@@H](CC[C@@]2(C)O)C1(C)C |
| 20123 | C[C@H]1CC[C@@H]([C@@H](C1)OC(C)=O)C(C)=C |
| 20125 | CC1CCC2(O)C1C1C(CCC2C)C1(C)C |
| 20132 | CC(C)C1(O)CCC(C)=CC1 |
| 20133 | CC(C)[C@@]12C[C@@H]1[C@@H](C)C(=O)C2 |
| 20134 | CC1=C[C@H](O)[C@@H]2C[C@H]1C2(C)C |
| 20261 | [H][C@]12C[C@]3([H])C(=C)CCC(O)C(C)(C)[C@]3([H])[C@@]1([H])[C@@H]2C |
| 20273 | CC1(O)CCC2OC1CCC(=C)C(O)CC2(C)C |
| 20359 | CC(=O)O[C@@H]1C[C@@H]2CC[C@]1(C)C2(C)C |
| 20361 | CC(=C)[C@@H]1CC=C(C)[C@@H](O)C1 |
| 20362 | CC(C)C1=CC=C(C=O)C=C1 |
| 20363 | CC1(C)C2CCC(C)(C2)C1=O |
| 20364 | CC(=C)C1(O)CC=C(C)C=C1 |
| 20365 | CC(C)=CCC\C(C)=C/COC(C)=O |
| 20366 | CC1(C)[C@@H]2C[C@H]1C(=C)[C@@H](O)C2 |
| 20367 | CC(C)[C@@]12C[C@@H]1C(C)C(=O)C2 |
| 20369 | CC1=CC(=O)[C@@H]2C[C@H]1C2(C)C |
| 20376 | CC(C)C12CC1C(=C)[C@H](O)C2 |
| 20388 | CC=CC=CC=O |
| 20403 | [H][C@@]12CC[C@@H](C)C3([H])CC[C@@](O)(CO)C3([H])[C@]1([H])C2(C)C |
| 20404 | CC1CCC2C1C1C(CC=C2CO)C1(C)C |
| 20405 | CC1CCC2C1C1C(CCC22CO2)C1(C)C |
| 20406 | [H][C@]12C3C(CC[C@@](O)(CO)[C@@]1([H])CCC2C)C3(C)C |
| 20407 | [H]C12C3C(CCC(C)(O)C1([H])CCC2C)C3(C)C |
| 20408 | CC1CCC2C1C1C(CCC2=O)C1(C)C |
| 20412 | CC(C)C1CC[C@]2(C)[C@@H](O)CCC(=C)C2[C@@H]1O |
| 20414 | CC1(C)C2CC[C@](C)(O)C3CC[C@@](C)(O)C3C12 |
| 20436 | CC(C)[C@H]1CC[C@@]2(C)[C@@H]1C(O)C(=C)CC[C@H]2O |
| 20455 | CC(C)C1CCC(C)=C2CC(O)C(C)=CC12 |
| 20460 | CC(C)(O)[C@H]1CC[C@](C)(O1)C=C |
| 20461 | [H][C@@]1(CC[C@](C)(O1)C=C)C(C)(C)O |
| 20462 | [H][C@@]1(CCC(C)(O1)C=C)C(C)(C)O |
| 20463 | CC(C)=CCC\C(C)=C/C=O |
| 20464 | CC(C)=CCC\C(C)=C/CO |
| 20465 | CC(C)C1CCC(=O)C=C1 |
| 20466 | CC(C)C1=CC=C(CO)C=C1 |
| 20467 | [H]C(=O)C1=CC=C(C=C1)C(C)C |
| 20482 | CC(C)C1CC[C@](C)(O)C=C1O |
| 20497 | COC(=O)C\C=C(/C)CCC1C(C)=CCC(O)C1(C)C |
| 20499 | COC(=O)C\C=C(/C)CCC1C(=C)CCC(O)C1(C)C |
| 20500 | COC(=O)C\C=C(/C)CCC1=C(C)CCC(O)C1(C)C |
| 20509 | C[C@@]1(O)CCC[C@]2(C)[C@H]3CC[C@H]4C[C@]3(CC[C@@H]12)C=C4O |
| 20525 | CC(C)[C@H]1CC[C@H](C)C23CC[C@](C)(O)[C@H]2[C@H]13 |
| 20546 | CC(=O)CCC1=CC=CC=C1 |
| 20547 | OCC1=CC=CC=C1 |
| 20550 | CC1CCC23C1C2C(CCC3C)C(C)(C)O |
| 20551 | COC1=CC(CC=C)=CC(OC)=C1OC |
| 20556 | COC1=C(OC)C=C(CC=C)C=C1 |
| 20557 | CC(=C)C1CCC(C)=C(O)C1 |
| 20558 | CC(=C)C1CCC(CO)=CC1 |
| 20559 | CC12OC1CC1CC2C1(C)C |
| 20560 | CC1(C)C2CC1C1(CO1)CC2 |
| 20563 | COC1=C(O)C=CC(CC=C)=C1 |
| 20565 | CC(=O)O[C@H]1C[C@H]2C[C@@H](C1=C)C2(C)C |
| 20567 | CC(C)[C@@H]1C[C@H](O)[C@@H](C)C(C1)OC(C)=O |
| 20568 | C[C@@H]1CCC2C(C)(C)C3C[C@@]12CCC3(C)O |
| 20570 | CC(C)=CC\C=C(\C)CCO |
| 20571 | CC(C)C1C=CC(C)CC1=O |
| 20572 | CC1CCC(CC1=O)=C(C)C |
| 20573 | CC(C)C1=CC=C(C)C(O)C1 |
| 20574 | CC(C)C1=CC=C(CO)CC1 |
| 20575 | C[C@H]1[C@H]2C[C@H](CC1=O)C2(C)C |
| 20576 | C[C@@H]1[C@@H]2C[C@H](CC1=O)C2(C)C |
| 20578 | CC(=O)OC(C)(C)C1CCC(C)=CC1 |
| 20579 | CC(C)C12CC1C(C)C(=O)C2 |
| 20580 | CC(C)[C@@]12C[C@@H]1[C@H](C)C(=O)C2 |
| 20653 | COC1=C(C(C)C)C(OC)=C(O)C(C)=C1 |
| 20654 | COC1=C(C(C)C)C(OC)=C(OC)C(C)=C1 |
| 20680 | CC(C)[C@@H]1CC[C@@](C)(O)C=C1 |
| 20681 | CC(C)[C@@H]1CC[C@@H](C)[C@@]23CC[C@](C)(O)[C@@H]2[C@@H]13 |
| 20683 | CC1=CC=C(C=C1)C(C)(C)O |
| 20684 | CC(C)[C@@]12CC1[C@](C)(O)CC2 |
| 21070 | [H][C@@]12CC[C@@H]3C[C@]1(C[C@@H]3CO)CC[C@]1([H])[C@](C)(O)CCC[C@@]21C |
| 21123 | COC(C)C1=CC(OC)=C(OC)C=C1OC |
| 21148 | C[C@]1(O)CCCC2(C)C1CCC13CCC(CC21)[C@](C)(O)C3 |
| 21187 | CC(C)[C@@H]1C[C@@H](O)C(C)=C[C@H]1O |
| 21188 | [H][C@@]12CC[C@@](C)(O)[C@]3([H])CC[C@@](C)(O)[C@@]3([H])[C@]1([H])C2(C)C |
| 21189 | [H][C@@]12CC[C@](C)(O)[C@@]3([H])CC[C@@](C)(O)[C@@]3([H])[C@]1([H])C2(C)C |
| 21190 | [H][C@@]12CC[C@](C)(O)[C@]3([H])CC[C@@](C)(O)[C@@]3([H])[C@]1([H])C2(C)C |
| 21192 | [H][C@@]12CC[C@@](C)(O)[C@@]3([H])CC[C@](C)(O)[C@@]3([H])[C@]1([H])C2(C)C |
| 21193 | [H][C@@]12CC[C@@](C)(O)[C@]3([H])CC[C@](C)(O)[C@@]3([H])[C@]1([H])C2(C)C |
| 21195 | [H][C@]12C[C@H](CC[C@]1(C)[C@@H]1CC[C@@]2(O)COO1)C(C)C |
| 21197 | CC(C)C1=C[C@@H]2[C@H](CC[C@@]2(C)O)C(=C)CC1 |
| 21229 | COC(=O)C1=CC(OC)=C(OC)C=C1 |
| 21255 | [H]C(=O)C1=C[C@@]2([H])[C@@H](CC[C@@]2(C)C(=O)CC1)C(C)C |
| 21257 | CC(C)[C@@H]1CC[C@@](C)(O)[C@@H]2CCC(C=O)=C[C@@H]12 |
| 21262 | COC1=CC2=C3C(=CC=C4C(=O)C=CC(C=C2)=C34)C1=O |
| 21263 | COC1=C(O)C=CC(\C=C\C=O)=C1 |
| 21266 | CC(C)[C@H](CCC(C)=O)[C@H]1[C@@H]2CCC(=O)[C@H]12 |
| 21283 | CC(=O)\C=C\C1=C(C)CCCC1(C)C |
| 21319 | [H][C@]12[C@H]3[C@@H](C[C@@](C)(O)[C@]1([H])CC[C@@]2(C)O)C3(C)C |
| 21320 | [H][C@]12[C@H]3[C@@H](C[C@@](C)(O)[C@]1([H])CC[C@]2(C)O)C3(C)C |
| 21321 | [H][C@]12[C@H]3[C@@H](C[C@](C)(O)[C@]1([H])CC[C@@]2(C)O)C3(C)C |
| 21322 | [H][C@@]1(C[C@H](O)[C@@](C)(O)C=C1)[C@@H](C)CCC=C(C)C |
| 21323 | [H][C@@]12[C@H](CC[C@@]1([H])[C@@](C)(O)CC[C@H]2C(C)=C)C(C)=O |
| 21326 | [H][C@@]12CC[C@](C)(O)[C@@]1([H])[C@H]1[C@@H](CC2=C)C1(C)C |
| 21331 | [H][C@@]12CCC(C)=C1C=C(CC[C@@]2([H])C)C(C)(C)O |
| 21332 | [H][C@@]12CCC(C)=C1C=C(CC[C@]2([H])C)C(C)(C)O |
| 21337 | [H][C@@]1(C)CCC(=CC2=C(C)CCC12)C(C)(C)O |
| 21357 | CC1(C)CC2C1CCC(=C)[C@@H](O)CCC2=C |
| 21368 | CC1=CC[C@@H](O)[C@]2(C)CC[C@H](C[C@@H]12)C(C)(C)O |
| 21369 | CC1=C2C[C@@H](CC[C@@]2(C)[C@@H](O)CC1)C(C)(C)O |
| 21372 | CC(C)C1CC(O)C(C)(O)C=C1 |
| 21374 | CC1(C)C[C@H]2[C@H]1CC[C@]1(O)CCC[C@@]2(C)C1 |
| 21379 | [H]C([H])=C([H])C1=C(OC)C=C(OC)C(OC)=C1 |
| 21430 | CC1(C)[C@@H]2CC[C@@](C)(C2)[C@@H]1O |
| 21445 | CC1(C)[C@H]2CC[C@]1(C)[C@H](O)C2 |
| 21446 | CC1(C)[C@H]2CC[C@]1(C)C(=O)C2 |
| 21487 | CC1=CC[C@H](CC=O)C1(C)C |
| 21489 | CC(C)[C@@]12C[C@@H]1C(=C)[C@@H](O)C2 |
| 21490 | C[C@H]1[C@@H]2C[C@H](CC1=O)C2(C)C |
| 21492 | CC(C)=CCCC1=COC=C1 |
| 21505 | C[C@@H]1CC[C@H]2C(C)(C)[C@H]3C[C@@]12CC[C@@]3(C)O |
| 21511 | COC1=C(O)C=CC(C=O)=C1 |
| 21518 | CC(C)C1C[C@@H](O)C(C)=C[C@H]1O |
| 21527 | CC\C=C/CC1=C(C)CCC1=O |
| 21541 | CC(C=C)=CC=CC(C)(C)O |
| 21560 | CCCCCCCCC=O |
| 21565 | CC1=CCC(C=C1)C(C)(C)O |
| 21569 | OC1=CC=C(CC=C)C=C1 |
| 21571 | C[C@@H]1CCC(=C(C)C)C(=O)C1 |
| 21575 | COC1=CC(OC)=C2C(=O)CC(OC2=C1)C1=CC=CC=C1 |
| 21607 | C[C@@]1(O)CCCC2(C)C1CCC13CCC(CC21)[C@](C)(O)C3 |
| 21615 | CC(C)=CCC\C(C)=C\COC=O |
| 21633 | C[C@H]1CCC2C1[C@@H]1[C@H](CCC22CO2)C1(C)C |
| 21635 | CC1CC[C@@H]2[C@@H]1[C@@H]1[C@H](CCC22CO2)C1(C)C |
| 21636 | CC1(C)[C@H]2CCC(=C)[C@H]3CC[C@@](C)(O)[C@@H]3[C@@H]12 |
| 21637 | CC1CCC2C1C1C(CC3OC23C)C1(C)C |
| 21638 | CCC(C)(O)CCCC(C)=C |
| 21639 | CC1(CO1)C1CCC2(C)OC2C1 |
| 21648 | [H][C@@]12C[C@@](O)(CC[C@@]1(C)CCCC2=C)C(C)(C)O |
| 21833 | COC1=C(OC)C=C2C=CC(=O)OC2=C1 |
| 21912 | CC(C)C1=CC2=C(C)C(=O)CC[C@@]2(C)C[C@H]1O |
| 21935 | [H][C@@]1(OCC2=C1C(C)=CC=C2)[C@]1([H])CC(=O)OC1(C)C |
| 21999 | COC1=CC=C2C=CC(=O)OC2=C1 |
| 22271 | C[C@@H]1CC(=O)C=C(C)[C@]11CC[C@](O)(C1)C(C)=C |
| 22272 | CC1=CC=CC(C=O)=C1C[C@@H]1CC(=O)OC1(C)C |
| 22273 | CC1=CC=CC(CO)=C1C[C@@H]1CC(=O)OC1(C)C |
| 5406 | CC(=C)[C@]1(O)CC[C@]2(C)CC(=O)C=C(C)[C@H]2C1 |
| 5467 | C[C@]1(O)CCC[C@@]2(C)C=CC(=O)C[C@@H]12 |
| 5469 | C[C@@H]1CC[C@@H]2[C@@H]1[C@@H]1[C@H](CC[C@@]2(C)O)C1(C)C |
| 5470 | C[C@H]1CC[C@@H]2[C@@H]1[C@H]1[C@@H](CC[C@]2(C)O)[C@@]1(C)CO |
| 5471 | COC(=O)[C@@]1(C)[C@@H]2CC[C@@](C)(O)[C@@H]3CC[C@@H](C)[C@H]3[C@H]12 |
| 5571 | CC1=COC2=C1[C@@](C)(O)C1=C(C)C=CC=C1C2=O |
| 5646 | C[C@@H]1CC[C@@](C)(O)[C@H]2CC[C@@H]3[C@H]([C@H]12)C3(C)C |
| 5649 | C[C@@H](CCC(C)=O)[C@H]1C[C@H](CCC1=O)C(C)=C |
| 5650 | CO[C@@H]1C[C@]2(C)CC[C@H](C[C@H]2C(C)=C1)C(=C)C(O)=O |
| 5651 | COC(=O)C(=C)[C@H]1CC[C@]2(C)CC=C[C@@](C)(O)[C@H]2C1 |
| 5664 | C[C@@H]1[C@@H]2CCC(C)(C)[C@@H]2C2=C(C)C(=O)C[C@]12O |
| 5681 | COC1=C2C=CC=C(C)C2=C(C)C2=C1OC=C2CO |
| 5763 | C[C@H]1[C@@H](O)C[C@H](O)C2=CC[C@@H](C[C@@]12C)C(C)=C |
| 5764 | C[C@@H]1CC[C@@H]2O[C@@]22CC3=C(C[C@@]12C)C(C)=CO3 |
| 6315 | CC(=O)CC[C@@H](CC1=C(C)CCC1=O)C(C)=C |
| 1033 | [H]C1CC(=C)[C@]2(O)C[C@]3([H])C(=C)C(=O)O[C@]3([H])C[C@@]2(C)C1 |
| 12003 | [H]\C1=C(C)/CC\C=C(C)\C[C@H](O)[C@@]2([H])C(=C)C(=O)O[C@]12[H] |
| 12007 | [H]\C1=C(C)/CC\C=C(C)\C[C@@H](OC(C)=O)[C@@]2([H])C(=C)C(=O)O[C@]12[H] |
| 12022 | [H]\C1=C(C)/[C@@H](O)C\C=C(C)\CC[C@@]2([H])C(=C)C(=O)O[C@]12[H] |
| 12024 | [H]\C1=C(C)/[C@H](C\C=C(C)\CC[C@@]2([H])C(=C)C(=O)O[C@]12[H])OC(C)=O |
| 12032 | [H]\C1=C(C)/CC\C=C(C)\[C@@H](O)C[C@@]2([H])C(=C)C(=O)O[C@]12[H] |
| 12055 | [H]\C1=C(CO)/CC\C=C(C)\CC[C@@]2([H])C(=C)C(=O)O[C@]12[H] |
| 12104 | [H][C@]12O[C@]1(C)CC\C=C(C)\CC[C@@]1([H])C(=C)C(=O)O[C@]21[H] |
| 12130 | [H]\C1=C(C)/[C@@H](O)C\C=C(C)\CC[C@@]2([H])[C@@H](C)C(=O)O[C@]12[H] |
| 12133 | [H]\C1=C(C)/CC\C=C(C)\C[C@H](O)[C@@]2([H])[C@H](C)C(=O)O[C@]12[H] |
| 12134 | [H]\C1=C(C)/CC\C=C(C)\C[C@H](OC(C)=O)[C@@]2([H])[C@H](C)C(=O)O[C@]12[H] |
| 12137 | [H]\C1=C(C)/CC\C=C(C)\[C@H](C[C@@]2([H])[C@H](C)C(=O)O[C@]12[H])OC(C)=O |
| 12138 | [H]\C1=C(CO)/CC\C=C(C)\CC[C@@]2([H])[C@H](C)C(=O)O[C@]12[H] |
| 12141 | [H][C@]12O[C@]1(C)CC\C=C(C)\CC[C@@]1([H])[C@H](C)C(=O)O[C@]21[H] |
| 12157 | [H]C1C\C=C(C)\C[C@]2([H])OC(=O)C(=C)[C@@]2([H])[C@H](O)\C([H])=C1/C |
| 12170 | [H]C1=C2CC\C=C(C)\C[C@]3([H])OC(=O)C(=C)[C@@]3([H])[C@@H]1OC2=O |
| 12182 | [H]\C1=C(C)/CC\C=C(C)\C[C@]2([H])OC(=O)[C@H](C)[C@@]2([H])[C@@H]1O |
| 12189 | [H]\C1=C(C)/CC[C@@]2([H])O[C@]2(C)C[C@@]2([H])OC(=O)C(=C)[C@@]2([H])C1 |
| 12190 | [H][C@]12C[C@]3([H])C(=C)C(=O)O[C@]3([H])C\C(C)=C\CC[C@]1(C)O2 |
| 12213 | [H][C@@]12CC[C@@]3(C)CCC=C(C)[C@]3([H])[C@@]1([H])OC(=O)C2=C |
| 12214 | [H][C@@]12CC[C@@]3(C)[C@@H](O)CC=C(C)[C@]3([H])[C@@]1([H])OC(=O)C2=C |
| 12230 | [H]C1CCC(C)=C2[C@@]3([H])OC(=O)C(=C)[C@]3([H])CC[C@@]12C |
| 12232 | [H]C1CCC(C)=C2[C@@]3([H])OC(=O)C(=C)[C@]3([H])[C@@H](O)C[C@@]12C |
| 12238 | [H]C1C[C@@]2(C)C([H])CCC(=C)[C@]2(O)[C@@]2([H])OC(=O)C(=C)[C@]12[H] |
| 12241 | [H][C@@]12CC[C@@]3(C)[C@H](O)CCC(=C)[C@]3([H])[C@@]1([H])OC(=O)C2=C |
| 12253 | [H]C1C[C@@]2(C)C=CC=C(C)[C@]2([H])[C@@]2([H])OC(=O)C(=C)[C@]12[H] |
| 12255 | [H][C@@]12CC[C@@]3(C)C=CC(=O)[C@@H](C)[C@]3([H])[C@@]1([H])OC(=O)C2=C |
| 12260 | [H]C1CC[C@@](C)(O)[C@]2([H])[C@@]3([H])OC(=O)C(=C)[C@]3([H])CC[C@@]12C |
| 12265 | [H]C1C=C(C)[C@]2([H])[C@@]3([H])OC(=O)[C@@]([H])(C)[C@]3([H])CC[C@@]2(C)[C@@H]1O |
| 12268 | [H][C@]1(C)C(=O)O[C@]2([H])C3=C(C)CC[C@H](O)[C@]3(C)CC[C@@]12[H] |
| 12270 | [H][C@@]12CC[C@@]3(C)C(=O)CCC(C)=C3[C@@]1([H])OC(=O)[C@@H]2C |
| 12272 | [H][C@@]12CC[C@@]3(C)CCC(=O)C(C)=C3[C@@]1([H])OC(=O)[C@H]2C |
| 12273 | [H]C1C[C@@]2(C)C=CC(=O)C(C)=C2[C@@]2([H])OC(=O)[C@@]([H])(C)[C@]12[H] |
| 12280 | [H][C@@]1(C)C(=O)O[C@@]2([H])[C@@]1([H])CC[C@@]1(C)CCCC(=C)[C@]21[H] |
| 12281 | [H][C@@]1(C)C(=O)O[C@@]2([H])[C@@]1([H])CC[C@@]1(C)[C@H](O)CCC(=C)[C@]21[H] |
| 12288 | [H][C@@]12CC[C@@]3(C)CCC[C@@](C)(O)[C@]3([H])[C@@]1([H])OC(=O)[C@H]2C |
| 12300 | [H][C@]12CC[C@@]3(C)O[C@@]13[C@H]1OC(=O)C(=C)[C@@H]1CC[C@H]2C |
| 12320 | [H][C@@]12[C@@H]3OC(=O)[C@H](C)C3=CC[C@@]1(C)CCC(=O)[C@@H]2C |
| 12322 | [H][C@]12C[C@@]3(C)[C@H](O)CC=C(C)[C@]3([H])C[C@]1([H])C(=C)C(=O)O2 |
| 12325 | [H][C@]12C[C@@]3(C)[C@H](O)CCC(C)=C3C[C@]1([H])C(=C)C(=O)O2 |
| 12330 | [H]C1C(=O)C=C(C)[C@]2([H])C[C@]3([H])C(=C)C(=O)O[C@]3([H])C[C@@]12C |
| 12334 | [H]C1[C@@H](O)C=C(C)[C@]2([H])C[C@]3([H])C(=C)C(=O)O[C@]3([H])C[C@@]12C |
| 12338 | [H][C@@]12C[C@@]3(C)C=CC(=O)C(C)=C3C[C@]1([H])C(=C)C(=O)O2 |
| 12339 | [H]C1CC(=C)[C@]2([H])C[C@]3([H])C(=C)C(=O)O[C@]3([H])C[C@@]2(C)C1 |
| 12340 | [H]C1CC(=C)[C@]2([H])C[C@]3([H])C(=C)C(=O)O[C@]3([H])C[C@@]2(C)[C@@H]1O |
| 12343 | [H][C@@]12C[C@@]3(C)C[C@@H](O)CC(=C)[C@]3([H])C[C@]1([H])C(=C)C(=O)O2 |
| 12344 | [H][C@@]12C[C@@]3(C)C[C@H](CC(=C)[C@]3([H])C[C@]1([H])C(=C)C(=O)O2)OC(C)=O |
| 12350 | [H]C1C[C@]2(C)C[C@@]3([H])OC(=O)C(=C)[C@@]3([H])C[C@@]2([H])C(=C)[C@@H]1O |
| 12352 | [H][C@@]12C[C@@]3(C)C=CC(=O)C(=C)[C@]3([H])C[C@]1([H])C(=C)C(=O)O2 |
| 12353 | [H][C@@]12C[C@@]3(C)CCC(=O)C(=C)[C@]3([H])C[C@]1([H])C(=C)C(=O)O2 |
| 12354 | [H][C@@]12C[C@@]3(C)C=C[C@H](O)C(=C)[C@]3([H])C[C@]1([H])C(=C)C(=O)O2 |
| 12355 | [H][C@@]12C[C@@]3(C)C=C[C@H](OC(C)=O)C(=C)[C@]3([H])C[C@]1([H])C(=C)C(=O)O2 |
| 12357 | [H]C1C[C@H](C)C2=C[C@]3([H])C(=C)C(=O)O[C@]3([H])C[C@@]2(C)C1[H] |
| 12358 | [H]C1C[C@H](C)C2=C[C@]3([H])C(=C)C(=O)O[C@]3([H])C[C@@]2(C)[C@@H]1O |
| 12359 | [H]C1[C@@H](O)C[C@H](C)C2=C[C@]3([H])C(=C)C(=O)O[C@]3([H])C[C@@]12C |
| 12360 | [H][C@]12C[C@@]3(C)CCCC(C)=C3C[C@]1([H])C(=C)C(=O)O2 |
| 12361 | [H][C@@]12C[C@@]3(C)CC(=O)C[C@H](C)C3=C[C@]1([H])C(=C)C(=O)O2 |
| 12363 | [H][C@@]12C[C@@]3(C)C=C[C@H]4O[C@@]4(C)[C@]3([H])C[C@]1([H])C(=C)C(=O)O2 |
| 12372 | [H][C@]12C[C@@]3([H])[C@H](C)C(=O)CC[C@]3(C)C[C@@H]1OC(=O)C2=C |
| 12373 | [H][C@@]1(C)C(=O)O[C@]2([H])C[C@@]3(C)C=CC(=O)C(=C)[C@]3([H])C[C@]12[H] |
| 12375 | [H][C@@]12C[C@@]3(C)CCC[C@H](C)C3=C[C@]1([H])[C@H](C)C(=O)O2 |
| 12377 | [H][C@@]1(C)C(=O)O[C@]2([H])C[C@@]3(C)CCCC(=C)[C@]3([H])C[C@]12[H] |
| 12381 | [H][C@]12C[C@@]3(C)CCCC(=C)[C@]3([H])CC1=C(C)C(=O)O2 |
| 12382 | [H][C@@]12CC3=C(C)C(=O)O[C@@]3(O)C[C@@]1(C)CCCC2=C |
| 12384 | [H][C@@]12C[C@@]3(C)CCC[C@H](C)C3=CC1=C(C)C(=O)O2 |
| 12385 | [H][C@@]12C[C@@]3(C)CCC[C@H](C)C3=CC1=C(CO)C(=O)O2 |
| 12386 | [H][C@@]12CC3=C(C)C(=O)OC3=C[C@@]1(C)CCCC2=C |
| 12387 | [H][C@@]12CC3=C(C[C@@]1(C)CCCC2=C)OC(=O)[C@@H]3C |
| 12389 | [H][C@@]12CC[C@]3(C)[C@@]4(C)C(=O)C=C(C)[C@@]34[C@@]1([H])OC(=O)[C@H]2C |
| 12392 | [H]C1C([C@@H](C)CCC(=O)OC)=C(C)C[C@@]2([H])OC(=O)C(=C)[C@@]12[H] |
| 12436 | [H]C1C([H])C(=C)[C@]2([H])CC(=O)C(=C)[C@]2([H])[C@H]2OC(=O)C(=C)[C@H]12 |
| 12457 | [H][C@]12OC(=O)C(=C)[C@]1([H])[C@H](CC(C)=C1CC=C(C)[C@H]21)OC(C)=O |
| 12459 | [H][C@]12OC(=O)C(=C)[C@]1([H])[C@@H](O)CC(C)=C1CC=C(C)[C@H]21 |
| 12473 | [H]C1CC(C)=C2C(=O)C=C(C)[C@]2([H])[C@@]2([H])OC(=O)C(=C)[C@]12[H] |
| 12479 | [H]C1[C@H](C)C(C)=C2C[C@H]3O[C@@]3(C)[C@]2([H])[C@@]2([H])OC(=O)C(=C)[C@]12[H] |
| 12485 | [H][C@@]12CC[C@]3(C)O[C@@]33C[C@@H]4O[C@]4(C)[C@]3([H])[C@@]1([H])OC(=O)C2=C |
| 12491 | C\C1=C/C[C@H]2[C@@H](C\C(C=O)=C/CC1)OC(=O)C2=C |
| 12501 | [H][C@@]12CC=C(C)[C@]3([H])CC(=O)[C@@H](C)[C@]3([H])[C@@]1([H])OC(=O)C2=C |
| 12503 | [H][C@]12OC(=O)C(=C)[C@]1([H])[C@H](O)CC(=C)[C@@H]1CC=C(C)[C@]21[H] |
| 12508 | [H]C1[C@H]2O[C@@]2(C)[C@]2([H])[C@@]3([H])OC(=O)C(=C)[C@]3([H])C([H])CC(=C)[C@]12[H] |
| 12518 | [H]C1CC(C)=C2C(=O)C=C(C)[C@]2([H])[C@@]2([H])OC(=O)[C@@]([H])(C)[C@]12[H] |
| 12530 | [H][C@]1(C)C(=O)O[C@@]2([H])[C@@]1([H])[C@H](O)C=C(C)[C@]1([H])CCC(=C)[C@]21[H] |
| 12532 | [H][C@@]12CC=C(C)[C@]3([H])CCC(=C)[C@]3([H])[C@@]1([H])OC(=O)[C@H]2C |
| 12548 | [H]C1[C@H]2O[C@@]2(C)[C@@]2([H])C1=C(C)CC([H])[C@@]1([H])[C@@H](C)C(=O)O[C@]21[H] |
| 12552 | [H][C@@]12CC[C@@]3(C)O[C@]33CC=C(C)[C@]3([H])[C@@]1([H])OC(=O)[C@H]2C |
| 12561 | [H][C@@]12CC=C(C)[C@]3([H])CC(=O)[C@@H](C)[C@]3([H])[C@@]1([H])OC(=O)[C@H]2C |
| 12562 | [H][C@@]1(C)C(=O)O[C@@]2([H])[C@@]1([H])[C@H](O)CC(=C)[C@@]1([H])CC=C(C)[C@]21[H] |
| 12578 | [H][C@@]1(C)C(=O)O[C@@]2([H])[C@@]1([H])CCC(=C)[C@]1([H])CCC(=C)[C@]21[H] |
| 12581 | [H]C1C[C@@]2([H])C(=C)CC[C@@]3(O)[C@@]([H])(C)C(=O)O[C@]3([H])[C@@]2([H])C1=C |
| 12584 | [H][C@@]12CCC(=C)[C@]3([H])CC(=O)[C@@H](C)[C@]3([H])[C@@]1([H])OC(=O)[C@H]2C |
| 12592 | [H][C@@]12CC[C@](C)(O)C3=CCC(C)=C3[C@@]1([H])OC(=O)[C@H]2C |
| 12593 | [H][C@@]12CC[C@@H](C)C3=CC(=O)[C@@H](C)[C@]3([H])[C@@]1([H])OC(=O)[C@H]2C |
| 12594 | [H][C@]12CCC(C)=C1[C@@]1([H])OC(=O)C(=C)[C@]1([H])CC[C@H]2C |
| 12596 | [H][C@]12C[C@@H](C)[C@]3(CCC(C)=C3[C@@]3([H])OC(=O)C(=C)[C@]13[H])O2 |
| 12598 | [H][C@@]12CC(=O)C(C)=C1[C@]1([H])OC(=O)C(=C)[C@]1([H])[C@H]1C[C@@]21C |
| 12661 | [H][C@@]12CCC(=C)[C@]1([H])[C@@H](OC(C)=O)[C@H]1[C@H](CC2=C)OC(=O)C1=C |
| 12663 | [H][C@@]12CC[C@@](C)(O)[C@]1([H])C[C@H]1[C@H](CC2=C)OC(=O)C1=C |
| 12664 | [H][C@@]12CC[C@@]3(C)O[C@@]13C[C@H]1[C@H](C[C@@H]2C)OC(=O)C1=C |
| 12666 | [H][C@@]12C[C@H]3[C@H](C[C@H](C)C1=CC(=O)[C@@H]2C)OC(=O)C3=C |
| 12667 | [H]C1[C@H]2[C@H](C[C@H](C)[C@]3([H])CC(=O)C(C)=C13)OC(=O)C2=C |
| 12669 | C[C@@H]1[C@H]2CC3=C(C)CCC3=C(C)C[C@@H]2OC1=O |
| 12672 | [H]C1C[C@@]2([H])C(=C)C[C@@]3([H])OC(=O)C(=C)[C@@]3([H])C[C@@]2([H])C1=C |
| 12674 | [H][C@@]12CCC(=C)[C@]1([H])[C@@]1([H])OC(=O)C(=C)[C@]1([H])CCC2=C |
| 12676 | [H]C1CC(=C)[C@]2([H])C[C@@H](O)C(=C)[C@]2([H])[C@H]2OC(=O)C(=C)[C@]12[H] |
| 12678 | [H]C1CC(=C)[C@]2([H])C[C@H](OC(C)=O)C(=C)[C@]2([H])[C@H]2OC(=O)C(=C)[C@]12[H] |
| 12681 | [H]C1C[C@@]2([H])C(=C)CC([H])[C@]3(O)[C@H](OC(=O)C3=C)[C@@]2([H])C1=C |
| 12712 | [H]C1C[C@@]2([H])C(C)=CC([H])[C@@]3([H])C(=C)C(=O)O[C@]3([H])[C@@]2([H])C1=C |
| 12738 | [H][C@@]12CC(=C)[C@]3([H])CC=C(C)[C@]3([H])C[C@]1([H])C(=C)C(=O)O2 |
| 12739 | [H][C@@]12C[C@]3([H])C(=C)C(=O)O[C@]3([H])CC(C)=C1C(=O)C=C2C |
| 12740 | [H][C@@]12CC(C)=C3CC[C@@](C)(O)[C@]3([H])C[C@]1([H])C(=C)C(=O)O2 |
| 12742 | [H][C@@]12CC(C)=C3CC[C@@](C)(OC(C)=O)[C@]3([H])C[C@]1([H])C(=C)C(=O)O2 |
| 12751 | [H]C1CC2=C(C)C(=C)[C@@]3([H])OC(=O)[C@@H](C)[C@@]3([H])C[C@@]2([H])[C@]1(C)O |
| 12767 | [H][C@@]12CC[C@@](C)(O)[C@]1([H])C[C@]1([H])C(=C)C(=O)O[C@]1([H])C=C2C |
| 12778 | [H]C(=O)C1=C/C[C@]2([H])C(=C)C(=O)O[C@@]2([H])C\C(C)=C\CC\1 |
| 12787 | [H]C1C\C(C)=C/[C@@H](O)C\C(C)=C/[C@@]2([H])OC(=O)C(=C)[C@]12[H] |
| 12803 | [H]\C1=C(C)/CC[C@@H](O)C(=C)CC[C@@H]2[C@@H]1OC(=O)C2=C |
| 12807 | [H]\C1=C(C)/CCC(=O)C(=C)CC[C@@H]2[C@@H]1OC(=O)C2=C |
| 12810 | C[C@H]1[C@@H]2CCC(=C)[C@H](O)CC\C(C)=C\[C@H]2OC1=O |
| 12821 | [H][C@@]12C\C(C)=C\CCC(=C)[C@]([H])(O)C[C@]1([H])C(=C)C(=O)O2 |
| 12902 | [H][C@@]12C[C@H](C)[C@H](O)C\C=C(C)\CC[C@@]1([H])[C@@H](C)C(=O)O2 |
| 12903 | [H][C@@]12C[C@H](C)C(=O)C\C=C(C)\CC[C@@]1([H])[C@@H](C)C(=O)O2 |
| 12937 | [H]C1CC(=C)[C@]2([H])CC(=O)[C@@H](C)[C@]2([H])[C@H]2OC(=O)C(=C)[C@H]12 |
| 12944 | [H]C1C=C(C)[C@]2([H])[C@H]3OC(=O)C(=C)[C@@H]3C([H])C[C@@](C)(O)[C@]12[H] |
| 12953 | [H][C@@]12[C@H]3OC(=O)C(=C)[C@@H]3CC[C@H](C)[C@]1([H])C(=O)C=C2C |
| 12964 | [H][C@]12CCC(=C)[C@]1([H])[C@H]1OC(=O)C(=C)[C@@H]1CC[C@@]2(C)O |
| 12965 | [H][C@@]12CCC(=C)[C@]1([H])[C@H]1OC(=O)C(C)=C1CCC2=C |
| 12966 | [H][C@@]12CCC(=C)[C@]1([H])[C@H]1OC(=O)C(CO)=C1CCC2=C |
| 12974 | C[C@H](O)CCC1=CC[C@H]2[C@H](C[C@@H]1C)OC(=O)C2=C |
| 12976 | C[C@H]1C[C@@H]2OC(=O)C(=C)[C@H]2CC=C1CCC(C)=O |
| 12980 | C[C@H]1C[C@@H]2OC(=O)C(=C)[C@H]2CC=C1\C=C/C(C)=O |
| 12983 | [H][C@@]12C[C@H]3[C@@H](C[C@]1(C)[C@H]2CCC(C)=O)OC(=O)C3=C |
| 12997 | [H][C@]1([C@H]2OC(=O)[C@@H](C)[C@@H]2[C@@H](O)C[C@@]1(C)C=C)C(C)=C |
| 13026 | [H][C@@]12CCC(=O)[C@@]1(C)[C@@H]1OC(=O)C(=C)[C@@H]1CC[C@@H]2C |
| 13040 | [H][C@@]12C=CC(=O)[C@@]1(C)[C@@H]1OC(=O)C(=C)[C@@H]1CC[C@@H]2C |
| 13046 | C[C@H]1CC[C@@H]2[C@@H](OC(=O)C2=C)[C@]2(C)C(=O)CC=C12 |
| 13058 | [H][C@@]12CCC(=O)[C@@]1(C)[C@@H]1OC(=O)[C@H](C)[C@@H]1CC[C@@H]2C |
| 13064 | [H][C@@]12CCC(=O)[C@@]1(C)C[C@H]1[C@@H](C[C@@H]2C)OC(=O)C1=C |
| 13071 | [H][C@@]12CC[C@@H](O)[C@@]1(C)C[C@H]1[C@@H](C[C@@H]2C)OC(=O)C1=C |
| 13078 | [H][C@@]12CCC(=O)O[C@@]1(C)[C@@H]1OC(=O)C(=C)[C@@H]1CC[C@@H]2C |
| 13079 | CC1=C2CCC(=O)O[C@@]2(C)[C@@H]2OC(=O)C(=C)[C@@H]2CC1 |
| 13082 | [H][C@]12CC(=O)O[C@]11[C@@H](C)CC[C@@H]3[C@@H](OC(=O)C3=C)[C@]21C |
| 13085 | [H][C@@]12C=CC(=O)[C@@]1(C)C[C@H]1[C@H](C[C@H]2C)OC(=O)C1=C |
| 13117 | [H][C@@]12[C@H]3CC(=O)[C@@]1(CO3)C[C@H]1[C@@H](C[C@H]2C)OC(=O)C1=C |
| 13149 | [H][C@@]12CC(=O)OC[C@@]1(C)C[C@H]1[C@H](C[C@H]2C)OC(=O)C1=C |
| 13157 | [H][C@@]12CCOC(=O)[C@@]1(C)C[C@H]1[C@@H](C[C@H]2C)OC(=O)C1=C |
| 13161 | C[C@@H]1C[C@H]2OC(=O)C(=C)[C@H]2C[C@H]2CC(=O)C(C)=C12 |
| 13165 | [H][C@@]12C[C@H]3[C@@H](C[C@@H](C)[C@]1([H])C=CC2=O)OC(=O)C3=C |
| 13166 | C[C@H]1CCC=C2C[C@H]3OC(=O)C(=C)[C@H]3C[C@]12C |
| 13167 | C[C@@H]1[C@H]2C[C@]3(C)[C@@H](C)CCC=C3C[C@H]2OC1=O |
| 13168 | [H][C@]12C[C@H]3OC(=O)C(C)=C3C[C@]1(C)[C@@H](C)C(=O)C=C2 |
| 13170 | [H][C@]12C[C@@]3(OC)OC(=O)C(C)=C3C[C@]1(C)[C@@H](C)C(=O)C=C2 |
| 13171 | [H][C@]12C[C@@]3(OCC)OC(=O)C(C)=C3C[C@]1(C)[C@@H](C)C(=O)C=C2 |
| 13172 | C[C@H]1CCC=C2C=C3OC(=O)C(C)=C3C[C@]12C |
| 13174 | C[C@H]1CCC=C2C=C3OC(=O)C(C)=C3[C@@H](OC(C)=O)[C@]12C |
| 13181 | [H][C@]12CCC[C@H](C)[C@@]1(C)[C@H](O)C1=C(C)C(=O)O[C@H]1C2 |
| 13186 | [H][C@@]12CCC[C@@H]3C(=O)O[C@@H](C4=C(C)C(=O)O[C@H]4C1)[C@@]23C |
| 13189 | [H][C@@]12CCC[C@@H]3C(=O)O[C@@H](C4=C(C1)OC=C4C)[C@@]23C |
| 13193 | [H]C1[C@@]2([H])CC([H])C([H])[C@H](C)[C@@]2(C)C[C@@]11C(=C)COC1=O |
| 13202 | [H]C1C[C@]2([H])[C@@H](OC(C)=O)[C@@]3(C[C@]2(C)[C@@H](C)C1[H])C(=C)COC3=O |
| 13212 | [H][C@@]12[C@H]3OC(=O)[C@@H](C)[C@@H]3CC[C@@H](C)[C@]1([H])C(=O)C=C2C |
| 13220 | [H][C@@]12CC[C@@]3(C)O[C@H]3[C@@]11OC(=O)C(=C)[C@@H]1CC[C@H]2C |
| 13244 | [H][C@@]12CC3=C(C[C@@]1(C)CC[C@H](OC(C)=O)C2=C)OC=C3C |
| 13245 | [H][C@@]12CC3=C(C[C@@]1(C)CC[C@H](O)C2=C)OC=C3C |
| 13248 | [H]C1[C@H]2[C@H](C)C(=O)O[C@@H]2[C@@]2([H])C(=C)CC[C@@]2([H])C(=C)[C@H]1O |
| 13350 | C[C@@]12CC[C@@H]3[C@H](OC(=O)C3=C)[C@@H]3O[C@]3(C)CC[C@H]1O2 |
| 13369 | CC(C)=CC(=O)C\C(C)=C\C[C@H]1COC(=O)C1=C |
| 13949 | [H][C@@]12C[C@H]3[C@@H](C[C@H](C)C1=CC(=O)O2)OC(=O)C3=C |
| 13950 | [H][C@]12C[C@H](C)[C@]34C=C[C@]5(C)C[C@@]3(C(=O)O1)[C@@]2([H])C[C@@]4([H])O5 |
| 14283 | [H]O[C@H]1C\C(C)=C\[C@H]2OC(=O)[C@@]([H])(C)[C@H]2CC\C(C)=C\1 |
| 14285 | [H][C@]1(C)[C@H]2CC[C@@]3(C)C[C@@H](O)CC(=C)[C@@H]3[C@H]2OC1=O |
| 14336 | C[C@H]1[C@H]2C=C3C(C)=CC(=O)C3=C(C)C[C@H]2OC1=O |
| 14374 | [H][C@@]12[C@H]3OC(=O)C(=C)[C@@H]3CC[C@@]1(C)[C@H](O)CC=C2C |
| 14377 | CC1CC2OC(=O)C(=C)C2(C)CC2C1=CC[C@]2(C)O |
| 14378 | CC(=O)O[C@H]1C[C@@H]2[C@H](OC(=O)C2=C)\C=C(C)\CC\C=C1/C |
| 14383 | [H]C([H])=C1[C@@H]2CC[C@H](C)CC(=O)C[C@H](C)C[C@H]2OC1=O |
| 14425 | C\C1=C/CC\C(C)=C\[C@H]2OC(=O)C(=C)[C@@H]2[C@H](O)C1 |
| 14434 | [H]C(=O)C1=C2C=CC(C)=C3CCC(=C)C3=C2OC1=O |
| 14435 | COC(=O)C1=C2C=CC(C)=C3CCC(=C)C3=C2OC1=O |
| 14436 | [H]C(=O)C1=C2C=CC(C)=C3CCC(C)C3=C2OC1=O |
| 14437 | COC(=O)C1=C2C=CC(C)=C3CCC(C)C3=C2OC1=O |
| 14442 | [H][C@]1(O)CCC(=C)[C@]2([H])[C@H]3OC(=O)C(=C)[C@@H]3CC[C@@]12C |
| 14443 | [H][C@@]1(CCC(=C)[C@]2([H])[C@H]3OC(=O)C(=C)[C@@H]3CC[C@@]12C)OC(C)=O |
| 14444 | [H][C@@]1(CC=C(C)[C@]2([H])[C@H]3OC(=O)C(=C)[C@@H]3CC[C@@]12C)OC(C)=O |
| 14447 | [H][C@@]12[C@H]3OC(=O)C(=C)[C@@H]3CCC(C)=C1C(=O)C(Cl)=C2C |
| 14486 | C[C@H]1[C@@H]2CC[C@@]3(C)[C@H](O)CCC(C)=C3[C@H]2OC1=O |
| 14492 | [H][C@@]12[C@H]3OC(=O)C(=C)[C@@H]3CCC(=C)[C@]1([H])C(=O)C=C2C |
| 14494 | [H][C@@]12[C@H]3OC(=O)C(=C)[C@@H]3CC=C(C)[C@]1(O)CC=C2C |
| 14495 | [H][C@@]12[C@H]3OC(=O)C(=C)[C@@H]3CCC(=C)[C@]1(O)CC=C2C |
| 14504 | [H][C@@]12CC=C(C)[C@]1([H])[C@H]1OC(=O)[C@@H](C)[C@@H]1CCC2=C |
| 14506 | [H]C1C=C(C)[C@]2([H])[C@H]3OC(=O)[C@@H](C)[C@@H]3CC[C@@]3(C)O[C@]123 |
| 14509 | CC1C=C(C)C2[C@H]3OC(=O)[C@@H](C)[C@@H]3CC[C@]3(C)O[C@@]123 |
| 14527 | C[C@@H]1[C@@H]2CCC(=C)C3C[C@H](O)C(=C)C3[C@H]2OC1=O |
| 14531 | [H]C([H])=C1C(=O)O[C@@]2([H])C[C@H](C)[C@]3([H])CC[C@@](C)(O)[C@]3([H])C[C@]12[H] |
| 14554 | [H][C@@]12CC[C@@](C)(O)[C@]1([H])C1OC(=O)C(=C)[C@@H]1CCC2=C |
| 15205 | C\C1=C\CC[C@@]2(C)O[C@H]2[C@H]2OC(=O)C(=C)[C@@H]2CC1 |
| 15338 | [H]C(CC(C)=C)C1[C@H](OC(=O)C1=C)C1=C(C)[C@@H](O)CC1C |
| 15339 | [H]C1CC(C)C([C@H]2OC(=O)C(=C)C2[C@@H](O)CC(C)=C)=C1C |
| 15529 | [H]\C1=C(C([H])([H])[H])/C([H])([H])C(=O)C2=C(C(=O)O[C@@]2([H])\C([H])=C(C([H])([H])[H])\C([H])([H])C1([H])[H])C([H])([H])[H] |
| 15532 | [H][C@@]12C[C@@]3(C)[C@@H]4C[C@@H]4C(=C)C3C(O)C1=C(C)C(=O)O2 |
| 15533 | CC1C(=O)OC2=C1[C@H](OC(C)=O)C1C(=C)[C@H]3C[C@H]3[C@]1(C)C2 |
| 15535 | [H][C@@]12C=C3CC(=O)O[C@@]3(O)C[C@@]1(C)C=CCC2=C |
| 15536 | [H][C@]12C[C@@]3(C)C=CCC(=C)[C@]3([H])CC1=CC(=O)O2 |
| 15537 | [H][C@@]12CC3=CC(=O)O[C@@]3(O)C[C@@]1(C)C=CCC2=C |
| 15538 | C[C@@H]1C2CC3=C(C)C(=O)O[C@H]3C[C@@]2(C)C=CC1=O |
| 15549 | CC1=C2[C@@H]3OC(=O)C(CC\C=C(/C)\C=C2\OC1=O)=C3 |
| 15551 | [H][C@]12C[C@@](C)(C=C)[C@@]([H])(CC1=C(C)C(=O)O2)C(=C)CO |
| 15554 | C[C@H]1[C@@H]2CC[C@@]3(C)[C@@H](CCC(C)=C3[C@H]2OC1=O)OC(C)=O |

## **References**

1. da Silva NM, de Carvalho RA, de Azeredo-Espin AM. Acetylcholinesterase cDNA sequencing and identification of mutations associated with organophosphate resistance in *Cochliomyia hominivorax* (Diptera: Calliphoridae). Vet Parasitol. 2011 177(1-2): 190-5. doi: <https://doi.org/10.1016/j.vetpar.2010.11.017>

2. Harel M, Kryger G, Rosenberry TL, Mallender WD, Lewis T, Fletcher RJ, Guss J, Silman I, Sussman JL. (2000), Three-dimensional structures of *Drosophila melanogaster* acetylcholinesterase and of its complexes with two potent inhibitors. Protein Science 2000 9: 1063-1072. <https://doi.org/10.1110/ps.9.6.1063>

3. Anstead CA, Korhonen PK, Young ND, Hall RS, Jex AR, Murali SC, Hughes DS, Lee SF, Perry T, Stroehlein AJ, Ansell BR, Breugelmans B, Hofmann A, Qu J, Dugan S, Lee SL, Chao H, Dinh H, Han Y, Doddapaneni HV, Worley KC, Muzny DM, Ioannidis P, Waterhouse RM, Zdobnov EM, James PJ, Bagnall NH, Kotze AC, Gibbs RA, Richards S, Batterham P, Gasser RB. *Lucilia cuprina* genome unlocks parasitic fly biology to underpin future interventions. Nat Commun. 2015 6:7344. doi: <https://doi.org/10.1038/ncomms8344>

4. Ren B, Peat TS, Streltsov VA, Pollard M, Fernley R, Grusovin J, Seabrook S, Pilling P, Phan T, Lu L, Lovrecz GO, Graham LD, Hill RJ. Unprecedented conformational flexibility revealed in the ligand-binding domains of the Bovicola ovis ecdysone receptor (EcR) and ultraspiracle (USP) subunits. Acta Crystallogr D Biol Crystallogr. 2014 70(7): 1954-64. doi: <https://doi.org/10.1107/S1399004714009626>

5. Kim IH, Pham V, Jablonka W, Goodman WG, Ribeiro JMC, Andersen JF. A mosquito hemolymph odorant-binding protein family member specifically binds juvenile hormone. J Biol Chem. 2017 292(37):15329-15339. doi: <https://doi.org/10.1074/jbc.M117.802009>

6. Scheuermann TH, Tomchick DR, Machius M, Guo Y, Bruick RK, Gardner KH. Artificial ligand binding within the HIF2alpha PAS-B domain of the HIF2 transcription factor. Proceedings of the National Academy of Sciences of the United States of America 2009 106(2): 450–455. doi: <https://doi.org/10.1073/pnas.0808092106>

7. Miller P, Aricescu A. Crystal structure of a human GABAA receptor. Nature 2014 512, 270–275. doi: <https://doi.org/10.1038/nature13293>

8. Yuan, D., Liu, Z., Kaindl, J. et al. Activation of the α2B adrenoceptor by the sedative sympatholytic dexmedetomidine. Nat Chem Biol 2020 16: 507–512. <https://doi.org/10.1038/s41589-020-0492-2>

9. Fan Yang, Shenglong Ling, Yingxin Zhou, Yanan Zhang, Pei Lv, Sanling Liu, Wei Fang, Wenjing Sun, Liaoyuan A Hu, Longhua Zhang, Pan Shi, Changlin Tian, Different conformational responses of the β2-adrenergic receptor-Gs complex upon binding of the partial agonist salbutamol or the full agonist isoprenaline, National Science Review 2021 8(9): nwaa284, <https://doi.org/10.1093/nsr/nwaa284>

11. Ma P, Weichert D, Aleksandrov L, Jensen TJ, Riordan JR, Liu X, Kobilka BK, Caffrey M. The cubicon method for concentrating membrane proteins in the cubic mesophase. Nat Protoc 2017 12: 1745–1762. <https://doi.org/10.1038/nprot.2017.057>

12. Ishchenko A, Stauch B, Han GW, Batyuk A, Shiriaeva A, Li C, Zatsepin N, Weierstall U, Liu W, Nango E, Nakane T, Tanaka R, Tono K, Joti Y, Iwata S, Moraes I, Gati C, Cherezov V. Toward G protein-coupled receptor structure-based drug design using X-ray lasers. IUCrJ. 2019 6(Pt 6):1106-1119. doi: <https://doi.org/10.1107/S2052252519013137>

13. Melo F, Sali A. Fold assessment for comparative protein structure modeling. Protein Sci. 2007 16(11): 2412-26. doi: <https://doi.org/10.1110/ps.072895107>

14. Shen MY, Sali A. Statistical potential for assessment and prediction of protein structures. Protein Sci. 2006 15(11): 2507-24. doi: <https://doi.org/10.1110/ps.062416606>

15. Modeller Documentation. model.assess_dope() -- assess a model with the DOPE method. <https://salilab.org/modeller/8v1/manual/node155.html> (2005)

16. Modeller Documentation. Model.assess_normalized_dope() — assess a model with the normalized DOPE method. <https://salilab.org/modeller/10.0/manual/node206.html#CMD:Model.assessnormalizeddope> (2022)

17. Modeller Documentation. Modeling with cryo-EM. <https://salilab.org/modeller/tutorial/cryoem/assess.html> (2021)

18. Ray A, Lindahl E, Wallner B. Improved model quality assessment using ProQ2. BMC Bioinformatics. 2012 13: 224. doi: <https://doi.org/10.1186/1471-2105-13-224>

19. Swiss-Model Documentation. Help SWISS-MODEL. [https://swissmodel.expasy.org](https://swissmodel.expasy.org/docs/help#:~:text=GMQE%20(Global%20Model%20Quality%20Estimate,score%20of%20the%20resulting%20model) (2022)

20. Biasini M, Bienert S, Waterhouse A, Arnold K, Studer G, Schmidt T, Kiefer F, Gallo Cassarino T, Bertoni M, Bordoli L, Schwede T. SWISS-MODEL: modelling protein tertiary and quaternary structure using evolutionary information. Nucleic Acids Res. 2014 42(Web Server issue): W252-8. doi: <https://doi.org/10.1093/nar/gku340>

21. Benkert P, Biasini M, Schwede T. Toward the estimation of the absolute quality of individual protein structure models. Bioinformatics. 2011 27(3): 343-50. doi: <https://doi.org/10.1093/bioinformatics/btq662>

22. Laskowski RA, Furnham N, Thornton JM. The Ramachandran Plot an Protein Structure Validation. Biomolecular Forms and Functions 2013: 62–75. doi: <https://doi.org/10.1142/9789814449144_0005>

23. Wiederstein M, Sippl MJ. ProSA-web: interactive web service for the recognition of errors in three-dimensional structures of proteins, Nucleic Acids Research 2007 35(2): W407–W410. doi: <https://doi.org/10.1093/nar/gkm290>

24. Saha C, Polash AH, Islam MT, Shafrin F. In silico prediction of structure and functions for some proteins of male-specific region of the human Y chromosome. Interdisciplinary Sciences: Computational Life Sciences 2013 5(4): 258–269. doi: https://doi.org/10.1007/s12539-013-0178-5

25. Cseke, L.J., Kirakosyan, A., Kaufman, P.B., Warber, S., Duke, J.A., Brielmann, H.L. Natural Products from Plants. 2 ed. Florida:Taylor & Francis Group, LLC; 2006.

26. Gao Y, Zhang Y, Wu F, Pei J, Luo X, Ju X, Zhao C, Liu G. Exploring the Interaction Mechanism of Desmethyl-broflanilide in Insect GABA Receptors and Screening Potential Antagonists by In Silico Simulations. Journal of Agricultural and Food Chemistry 2020 68(50): 14768-14780. doi: <https://doi.org/10.1021/acs.jafc.0c05728>

27. Rakonczay Z. 2003. Potencies and Selectivities of inhibitors of Acetylcholinesterase and its molecular forms in normal and Alzheimer’s disease brain. Acta Biologica Hungarica. 2003 54(2): 183-189. doi: <https://doi.org/10.1556/abiol.54.2003.2.7>

28. Jackisch R. Förster S. Kammerer M. Rothmaier AK. Ehret A. Zentner J. Feuerstein TJ. Inhibitory Potency of Choline Esterase Inhibitors on Acetylcholine Release and Choline Esterase Activity in Fresh Specimens of Human and Rat Neocortex. Journal of Alzheimer’s Disease 2009 16: 635–647. doi: <https://doi.org/10.3233/JAD-2009-1008>

29. Vecchio I. Sorrentino L. Paoletti A. Marra R. Arbitrio M. The State of The Art on Acetylcholinesterase Inhibitors in the Treatment of Alzheimer’s Disease. Journal of Central Nervous System Disease 2021 13: 1-13. doi: <https://doi.org/10.1177/11795735211029113>

30. Skibinski R. Czarnecka K. Girek M. Bilichowski I. Chufarova N. Mikiciuk-Olasik E. Szymanski P. 2017. Novel Tetrahydroacridine derivates with iodobenzoic acid moiety as multifunctional acetylcholinesterase inhibitors. Chem Biol Drug Des. 2017 91: 505-518. doi: <https://doi.org/10.1111/cbdd.13111>

31. Minakuchi C. Ogura T. Miyagawa H. Nakagawa Y. Effects of the structures of ecdysone receptor (EcR) and ultraspiracle (USP) on the ligand-binding activity of the EcR/USP heterodimer. Journal of Pesticide Science 2007 32(4): 379-384. doi: <https://doi.org/10.1584/jpestics.G07-19>

32. Watanabe B. Nakagawa Y. Ogura T. Miyagawa H. Stereoselective synthesis of (22R)- and (22S)-castasterone/ponasterone A hybrid compounds and evaluation of their molting hormone activity. Steroids. 2004 Jul;69(7):483-93. doi: <https://doi.org/10.1016/j.steroids.2004.04.005>

33. Trowell S. Hines ER. Herlt AJ. Rickards RW. Characterization of a juvenile hormone binding lipophorin from the blowfly *Lucilia cuprina*. Comparative biochemistry and physiology Part B. Biochemistry & molecular biology 1994 109(2-3): 339-57. [doi: https://doi.org/10.1016/0305-0491(94)90018-3](https://doi.org/10.1016/0305-0491(94)90018-3)

34. Kim IH. Pham V. Jablonka W. Goodman WG. Ribeiro JMC. Andersen JF. A mosquito hemolymph odorant-binding protein family member specifically binds juvenile hormone. J Biol Chem. 2017 Sep 15;292(37):15329-15339. doi: <https://doi.org/10.1074/jbc.M117.802009>

35. Yokoi T. Nabe T. Ishizuka C. Hayashi K. Ito-Harashima S. Yagi T. Nakagawa Y. Miyagawa H. Transcription-inducing activity of natural and synthetic juvenile hormone agonists through the *Drosophila* Methoprene-tolerant protein. Pest Manag Sci 2020 76: 2316-2323. doi: <https://doi.org/10.1002/ps.5766>

36. γ-Aminobutyric acid. In Wikipedia. Retrieved June 10. 2022. from <https://en.wikipedia.org/wiki/%CE%93-Aminobutyric_acid>

37. McGonigle I. Lummis SC. Molecular characterization of agonists that bind to an insect GABA receptor. Biochemistry 2010 49(13): 2897-2902. doi: <https://doi.org/10.1021/bi901698c>

38. Nakao T. Banba S. Nomura M. Hirase K. Meta-diamide insecticides acting on distinct sites of RDL GABA receptor from those for conventional noncompetitive antagonists. Insect Biochem Mol Biol. 2013 43(4): 366-75. doi: <https://doi.org/10.1016/j.ibmb.2013.02.002>

39. Ozoe Y. Kita T. Ozoe F. Nakao T. Sato K. Hirase K. Insecticidal 3-benzamido-N-phenylbenzamides speciﬁcally bind with high aﬃnity to a novel allosteric site in houseﬂy GABA receptors. Pestic Biochem Physiol. 2013 107(3): 285-92. doi:

<https://doi.org/10.1016/j.pestbp.2013.09.005>

40. Orr. GL. Hollingworth RM. Agonist-induced desensitization of an octopamine receptor. Insect Biochemistry 1990 20(3): 239-244. doi: <https://doi.org/10.1016/0020-1790(90)90040-2>

41. Dalwadi DA. Schetz JA. Comparative Exploration of the Structure-Activity Space of Cloned α-Like Octopamine Receptors from a Marine and a Terrestrial Arthropod. Mol Pharmacol. 2017 92(3): 297-309. doi: <https://doi.org/10.1124/mol.117.108456>

42. Beggs KT. Tyndall JD. Mercer AR. Honeybee dopamine and octopamine receptors linked to intracellular calcium signaling have a close phylogenetic and pharmacological relationship. PLoS One. 2011 6(11): e26809. doi: <https://doi.org/10.1371/journal.pone.0026809>

43. Roeder T. A new octopamine receptor class in locust nervous tissue. the octopamine 3 (OA3) receptor. Life Sciences 1992 50(1): 21-28
